# Graphene Chainmail Shelled Dilute Ni─Cu Alloy for Selective and Robust Aqueous Phase Catalytic Hydrogenation

**DOI:** 10.1002/advs.202304349

**Published:** 2024-01-19

**Authors:** Haifeng Yuan, Mei Hong, Xianzhen Huang, Weitao Qiu, Feng Dong, Yu Zhou, Yanpeng Chen, Jinqiang Gao, Shihe Yang

**Affiliations:** ^1^ Guangdong Provincial Key Lab of Nano‐Micro Materials Research, School of Advanced Materials, Shenzhen Graduate School Peking University Shenzhen Shenzhen Guangdong 518055 China; ^2^ Shenzhen Key Laboratory of Organic Pollution Prevention and Control, Environmental Science and Engineering Research Center Harbin Institute of Technology (Shenzhen) Shenzhen Guangdong 518055 China; ^3^ Insitute of Biomedical Engineering Shenzhen Bay Laboratory Shenzhen Guangdong 518055 China

**Keywords:** aqueous phase catalysis, dilute Cu–Ni alloys, Mott–Schottky heterojunction, oxygen‐doped carbon shell, selective hydrogenation

## Abstract

Cost‐effective non‐noble metal‐based catalysts for selective hydrogenation with excellent activity, selectivity, and durability are still the holy grail. Herein, an oxygen‐doped carbon (OC) chainmail encapsulated dilute Cu–Ni alloy is developed by simple pyrolysis of Cu/Ni‐metal–organic framework. The CuNi_0.05_@OC catalyst displays superior performance for atmospheric pressure transfer hydrogenation of *p*‐chloronitrobenzene and *p*‐nitrophenol, and for hydrogenation of furfural, all in water and with exceptional durability. Comprehensive characterizations confirm the close interactions between the diluted Ni sites, the base Cu, and optimized three‐layered graphene chainmail. Theoretical calculations demonstrate that the properly tuned lattice strain and Schottky junction can adjust electron density to facilitate specific adsorption on the active centers, thus enhancing the catalytic activity and selectivity, while the OC shell also offers robust protection. This work provides a simple and environmentally friendly strategy for developing practical heterogeneous catalysts that bring the synergistic effect into play between dilute alloy and functional carbon wrapping.

## Graphical abstract

Dilute alloy and chainmail synergy profoundly enhances catalytic activity. Trace Ni‐doping in cost‐effective CuNi_0.05_ induces properly tuned lattice strain and assembles three‐layered oxygen‐doped graphene chainmail rich in defects with enhanced Schottky junction. The CuNi_0.05_@OC displays superior efficiency and exceptional stability for mild hydrogenation of *p*‐chloronitrobenzene, *p*‐nitrophenol, and furfural in water.

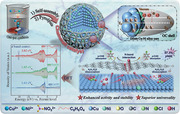

## 1 Introduction

Catalytic hydrogenation has wide applications ranging from petrochemistry to fine chemical industries. Heterogeneous catalysts with high activity, selectivity, and stability for aqueous phase hydrogenation are highly desirable owing to the environmentally friendliness and excellent applicability to biomass conversion involving high water content. Hydrogenation is one of the biggest challenges in catalysis field. To reach high activity, promoting and leveraging active sites have achieved some success but still remain difficult.^[^ ^1^ ^]^ For high selectivity, the ingenious strategy of “active site isolation” has been proven effective, such as tuning metal–support interactions, confining active metal nanoparticles (NPs) in porous supports, forming bimetallic alloys, and constructing single atom catalysts.^[^ ^2^ ^]^ For boosting stability, nanospace, and interfacial confinements, especially encapsulation with a protective carbon layer, prevent leaching and sintering of metal NPs.^[^ ^3^ ^]^

Recently, studies have revealed that carbon encapsulation not only acts as armor to improve the metal catalyst stability, but also works as chainmail to tune their activity.^[^ ^4^ ^]^ Electron transfer from the active metal core to the wrapping carbon layer helps deliver catalytic activity to the outer surface allowing synchronized cascading. The carbon chainmail approach enables non‐noble transition metals to replace noble metal counterparts, overcoming their limitations of low work function and high vulnerability to harsh conditions.^[^ ^5^ ^]^ The carbon chainmail strategy has been utilized to develop metal@C hybrid catalysts. Gutiérrez–Tarriño et al. prepared N‐doped carbon layer covered cobalt nanoclusters (Co@NC‐800) by pyrolysis of cobalt complex impregnated on Vulcan carbon, which achieved chemoselective nitroarene hydrogenation in water.^[^ ^6^ ^]^ Yan et al. developed Ni nanoparticles encapsulated by multilayer graphene‐like shells doped with oxygen‐containing functional groups via calcination of Ni‐based metal–organic framework (MOF), which delivered excellent activity and robust stability for nitrobenzene hydrogenation.^[^ ^7^ ^]^ Zhao et al. synthesized Pd single‐atom catalyst anchored to the shell of magnetic core–shell particles that consist of a Ni‐nanoparticle core and a graphene sheet shell (Pd/Ni@G) by a thermal decomposition method followed by deposition‐precipitation, which efficiently catalyzed phenylacetylene semi‐hydrogenation.^[^ ^8^ ^]^

Although controllable synthesis of chainmail catalyst is highly desirable to balance metal protection from harsh environment and reaction site activation, realizing such control is a huge challenge, as a proper choice of three layers have been proposed.^[^ ^4^ ^]^ With the cost‐effective copper catalyst, highly selective in hydrogenation reactions such as carbon dioxide reduction and furfural conversion,^[^ ^9^, ^10^ ^]^ instability during operation remains a major drawback.^[^ ^11^ ^]^ Because of the virtually zero equilibrium solubility of carbon in copper,^[^ ^12^ ^]^ direct pyrolysis of copper containing MOFs only resulted in Cu on carbon composite without chainmail structure.^[^ ^13^ ^]^ To achieve carbon wrapping of copper, additional supports such as silica have been employed. Lan et al. fabricated ultra‐dispersed Cu cluster encapsulated in N‐doped carbon‐coated mesoporous silica sphere (Cu/NC@NMSSs) by an incipient‐wetness impregnation method which demonstrated excellent performances in the hydrogenation of biomass‐derived levulinic acid into γ‐valerolactone.^[^ ^14^ ^]^ We hypothesized that adding another metal with high carbon solubility could tune the chainmail property such as shell thickness. The non‐noble metal nickel shows its superiority in dissolving carbon, displaying ≈25% carbon solubility in normal Ni NPs, comparable to noble metals.^[^ ^15^, ^16^ ^]^

Herein, we report the first chainmail graphene shelled dilute Ni─Cu alloy catalysts, CuNi*_x_* @OC, demonstrating not only controllable shell thickness and metal–support interactions, but also synergistic bimetallic alloy effect. The CuNi_0.05_@OC catalyst exhibited excellent activity, selectivity, and durability in various hydrogenation reactions encompassing commonly used hydrogen sources, including model reactions of *p*‐chloronitrobenzene (*p*‐CNB) conversion to *p*‐chloroaniline (*p*‐CAN) with hydrazine hydrate and *p*‐nitrophenol (*p*‐NP) reduction to *p*‐aminophenol (*p*‐AP) using sodium borohydride, and industrially important valorization of furfural (FF) toward furfuryl alcohol (FAOL) with hydrogen gas.

## 2 Results and Discussion

### 2.1 Structural Characterization of the CuNi_0.05_@OC Catalyst

**Figure 1:**
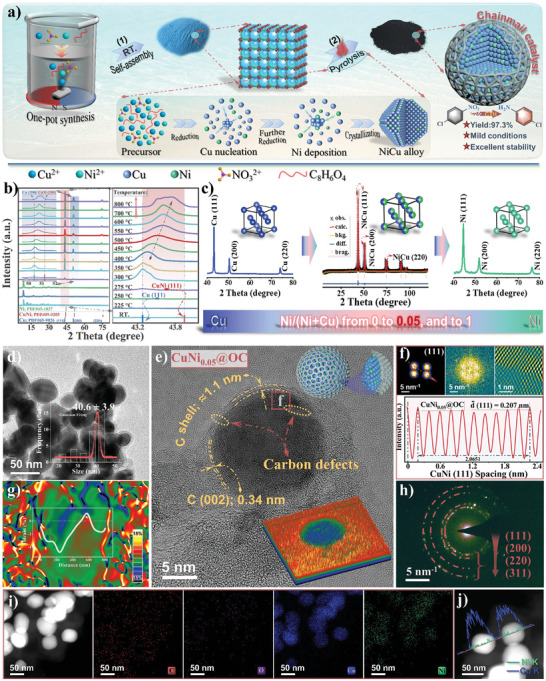
Synthesis and characterizations of CuNi_0.05_@OC catalysts: a) Illustration for the fabrication process. b) In situ XRD patterns at different reduction temperatures. c) Rietveld refinement and structure models. d) TEM image, inset is the NP size distribution. e) HR‐TEM image of a single NP, inset in the top right corner is the 3D illustrations and in bottom right is the corresponding 3D pseudo‐color surface plot. f) Inverse FFT pattern and integrated pixel intensity analysis. g) The strain map determined via GPA. h) SAED pattern. i) HAADF‐STEM image with elemental mappings, and j) the corresponding elemental line‐scanning profiles of the representative NPs.

The dilute Cu–Ni bimetallic catalyst encapsulated in three‐layer graphene sheets, CuNi_0.05_@OC, was prepared by a simple one‐pot method via Cu–Ni MOF pyrolysis (**Figure** **1**a). Because the Cu–Ni binary phase diagram shows a large miscibility gap (Figure S1, Supporting Information) and Cu has a higher reduction potential than Ni, the reduction of Cu^2+^ occurred first establishing Cu base,^[^ ^17^ ^]^ followed by trace Ni deposition forming dilute Cu–Ni alloy. At the same time, pyrolysis of the organic ligand yielded oxygen‐doped carbon network, wrapping the Cu–Ni alloy. To follow the formation process of dilute alloy in CuNi_0.05_@OC, the change of diffraction peaks during thermal reduction was monitored by in situ XRD (Figure 1b). Heating from 25 to 275 °C released physically adsorbed water and decomposed part of organics, and copper peak at 43.3° began to appear. Further increasing temperature gradually moves this diffraction peak to higher value, indicating Cu–Ni alloy formation (Figure 1c).^[^ ^18^ ^]^ The peak position remained constant at 43.5° between 550 to 700 °C with slight peak boarding thus 550 °C was chosen as the pyrolysis temperature. Typical weight percent of metal elements were determined by ICP‐AES (Table S1, Supporting Information) showing an extremely low molar ratio of 0.05 for Ni/Cu. The FE‐SEM images of the CuNi_0.05_@OC sample (Figure S2, Supporting Information) revealed coralloid‐like micro–nano multi‐dimensional morphology. The TEM images and pseudo‐color surface map (Figure 1d,e) confirmed that the metal NP core with an average size of ≈40 nm was tightly encapsulated by graphene. The shell shows an average thickness of 1.1 nm, that is ≈3 layers of carbon (002) planes with a spacing of 0.34 nm. The lattice fringes of CuNi_0.05_ NPs show a spacing of 0.207 nm from inverse FFT (Figure 1f), corresponding to the (111) planes of the CuNi_0.05_ alloy. Geometric phase analysis (GPA) in Figure 1g shows the strain diagram of representative particles. The significant color contrast revealed the strong compressive stress on the alloy particles coated with carbon shell. The strain effect could lead to a shift in the d‐band center and optimize binding energies of reaction intermediates in a catalytic process.^[^ ^19^ ^]^ The selected area electron diffraction (SAED) patterns (Figure 1h) also show the (111) plane of the CuNi_0.05_. HAADF‐STEM element mapping (Figure 1i) shows that CuNi_0.05_@OC was composed of host Cu and trace Ni, covered by oxygen‐doped graphene. The oxygen doping might result from terephthalic acid pyrolysis, which can alter work function of carbon and enhance catalytic activity of the metal‐carbon composite through electronic metal–support interactions.^[^ ^20^ ^]^ The EDX line profile of representative NP (Figure 1j) further confirmed the highly‐dispersed Ni on the pseudo core–shell CuNi_0.05_ dilute alloy.

### 2.2 Structural Regulation by Dilute Nickel Alloying

**Figure 2:**
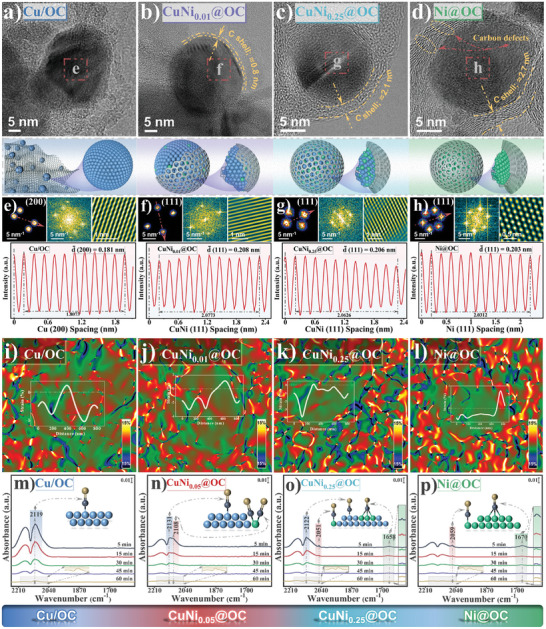
The comparative structural characterizations of Cu/OC, CuNi_0.01_@OC, CuNi_0.25_@OC, and Ni@OC: a–d) HR‐TEM images of single NP and the corresponding 3D structure illustrations. e–h) Inverse FFT patterns and the corresponding integrated pixel intensity analysis. i–l) The strain maps determined via GPA. m–p) In situ DRIFTS of CO adsorption. Cu: blue; Ni: green; C: gray; O: yellow.

Thickness of the oxygen‐doped graphene shell and compressive strain of the CuNi alloy core could be precisely tuned by the nickel fraction added to the composite. The XRD diffraction patterns in Figure S3a, Supporting Information, display a positive shift of (111) peak which value correlated with Ni content in the CuNi*_x_* @OC samples, confirming the successful alloying of Ni and Cu.^[^ ^21^ ^]^ All diffraction peaks can be attributed to elemental metals, and those ascribed to metal oxide are barely visible. The carbon shell probably inhibits air infiltration and deep oxidation of alloy particles. No diffraction peak of Ni could be detected for the dilute alloy CuNi*_x_* @OC (x ≤ 0.25), contrary to the high Ni‐content CuNi_0.65_@OC sample that displayed a tiny Ni metal peak related to phase separation causing Ni nanoparticles (Figure S3b, Supporting Information). The addition of Ni obviously altered morphology of the CuNi*_x_* @OC series (**Figure** **2**a–d). A positive correlation between Ni content and the number of graphene layers in the CuNi*_x_* @OC samples could be found (Figure S4, Supporting Information). Without Ni, the Cu NPs in Cu/OC were not uniform and anchored on carbon plate instead of being encapsulated. For the ultra‐dilute CuNi_0.01_@OC, thin but discontinuous graphene layer covered the CuNi alloy with a thickness <1 nm. The CuNi_0.05_@OC possesses the optimal 1.1 nm graphene shell of approximately three layers wrapping the entire inner metal core (Figure 1e), whereas increasing the Ni content as for the CuNi_0.25_@OC and Ni@OC samples, the graphene layer thickness increased to 2.1 and 2.7 nm, respectively. Accompanying the shell thickness modulation, the NP size decreased monotonically with the increasing Ni content, from 62.9 nm in Cu/OC to 40.6 nm in CuNi_0.05_@OC, 32.3 nm in CuNi_0.25_@OC, and 20.1 nm in Ni@OC. As shown in Figure S5, Supporting Information, the HAADF‐STEM images and EDX mappings of Cu/OC and Ni@OC shows sharp difference, which confirms their different metal‐carbon contacts. The corresponding lattice spacings of the samples obtained from the inverse FFT are all smaller than that of pure Cu (Figures 2e–h and 1f), revealing the rich compressive stress upon carbon encapsulation, consistent with XRD peak shifts. The strain maps with GPA analysis (Figure 2i–l and 1g) exhibit more compression in the inner part of the alloy nanoparticles especially with dilute Ni addition.

The dispersion of Ni in dilute alloy can be clarified by in situ DRIFTS of CO. For Cu/OC, the peak near 2119 cm^−1^ (Figure 2m) can be assigned to the atop binding of CO on Cu atoms.^[^ ^22^ ^]^ For Ni@OC, the spectra (Figure 2p) display both linear adsorption of CO at 2053 cm^−1^ and multi‐coordination adsorption at 1653 cm^−1^.^[^ ^23^ ^]^ These three peaks all exist in the spectra of CuNi_0.25_@OC (Figure 2o) displaying multiple adsorption modes. For the CuNi_0.05_@OC sample with trace amount of Ni, the multi‐coordination adsorption peak of CO below 2000 cm^−1^ becomes invisible indicating lack of Ni aggregates. Its spectra show a main peak at 2131 cm^−1^ and a shoulder peak at 2108 cm^−1^ (Figure 2n). The red‐shift of the former peak relative to Cu@OC may be due to the Cu–Ni bimetallic effect that decreases the electronic density of Cu weakening the bond between CO and Cu,^[^ ^23^, ^24^ ^]^ and the latter may be attributed to Ni(CO)*_x_* subcarbonyl species (*x* = 2 or 3) suggesting Ni atoms are atomically dispersed in the dilute alloy system.^[^ ^22^ ^]^ N_2_ adsorption–desorption isotherms (Figure S6 and Table S2, Supporting Information) give direct evidence of the carbon network structure. The highly porous nature of the catalysts could contribute to the atomic scale Ni dispersion and guest molecule adsorption.

**Figure 3:**
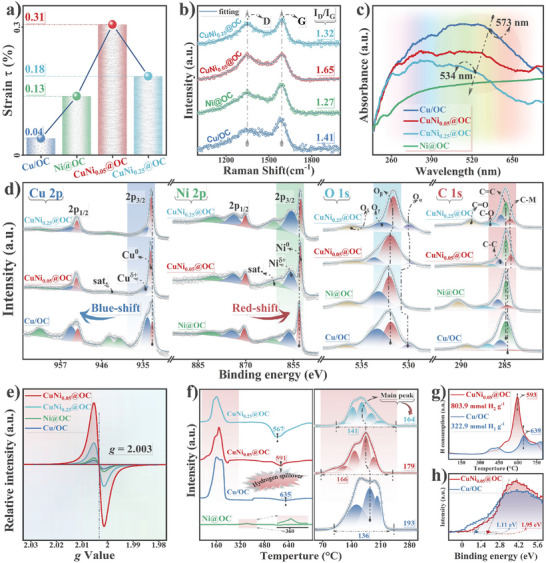
Structural analysis of Cu/OC, CuNi_0.05_@OC, CuNi_0.25_@OC, and Ni@OC: a) Variation of lattice strain (*τ*). b) Raman spectra, which displays the corresponding *I*_D_/*I*_G_ ratio values. c) UV–vis DRS patterns. d) HR‐XPS spectra. e) ESR spectra. f) H_2_‐TPR profile. g) H_2_‐TPD profile. h) Valence band spectra.

Based on the theoretical lattice constants of Cu, Ni, and bimetallic CuNi alloys, the compressive strains in the CuNi*_x_* @OC were obtained,^[^ ^25^ ^]^ as shown in **Figure** **3**a. Although the monometallic Cu/OC and Ni@OC endured 0.04% and 0.13% compressive strain, it increased to 0.31% and 0.18% for CuNi_0.05_@OC and CuNi_0.25_@OC, respectively. The volcano‐shaped strain profile with the largest strain on CuNi_0.05_@OC indicates that dilute Ni addition induced the strongest interaction between metal nanoparticles and the shell.^[^ ^26^ ^]^ The Raman spectra of the samples revealed rich structural defects in the carbon coating (Figure 3b) manifested by the *I*_D_/*I*_G_ area ratio. The D band and G band peaks at about 1360 and 1590 cm^−1^ belong to the vibration of disordered carbon defects and graphite structure with E2g symmetry.^[^ ^27^ ^]^ Among the monometallic Cu/OC or Ni@OC and the bimetallic CuNi*_x_* @OC catalysts, CuNi_0.05_@OC had the highest *I*_D_/*I*_G_ area ratio. Thus, the thin and continuous graphene shell contains the most abundant structural defects likely induced by the local lattice strain in the sample.^[^ ^25^ ^]^ For the metallic core, the optical UV–vis DRS spectra (Figure 3c) confirmed the confined NP size change. The localized surface plasmon resonances peak of copper gradually shifted from 573 nm for Cu/OC to lower wavelength of 534 nm for CuNi_0.25_@OC, reflecting decreased alloy size that alters the absorption by the nanoparticles.^[^ ^28^ ^]^

The high‐resolution X‐ray photoelectron spectroscopy (HR‐XPS) was used to determine the surface composition and electronic states in different catalysts. Figure S7, Supporting Information, displays the survey spectra confirming the presence of Cu, Ni, C, and O. Figure 3d shows the HR‐XPS of the corresponding elements, and Table S3, Supporting Information, compares the binding energy (BE) and peak area ratio. The surface Ni/Cu molar ratios were much higher than the bulk molar ratios in all samples, indicating that Ni atoms tend to distribute on the surface or subsurface of the Cu host. The obvious Cu 2p_3/2_ peaks can be deconvolved into Cu^δ+^ at ≈934 eV and Cu^0^ at ≈933 eV,^[^ ^29^ ^]^ accompanied by satellite peak of Cu^δ+^ around 940 eV.^[^ ^30^, ^31^ ^]^ Introduction of Ni in the dilute CuNi*_x_* @OC catalysts obviously blue‐shifted the Cu^δ+^ peak and lowered its intensity, along with almost disappearance of the satellite peak. Thus, Ni promoted Cu reduction and strengthened the metallicity of Cu, so that the proportion of Cu^0^ species increased significantly from 29% in Cu/OC to 62% in CuNi_0.05_@OC. In the Ni 2p spectrum of Ni@OC, a clear peak around 853 eV can be attributed to metallic Ni^0^, which position red‐shifted to lower BE upon alloying with Cu. The blue‐shift of Cu peaks and the red‐shift of Ni peaks confirm the strong electronic interaction between Cu and Ni. For the CuNi_0.01_@OC sample with Ni content <0.4 wt%, signal of Ni is almost unmeasurable in the Ni 2p spectrum (Figure S8, Supporting Information), while in the Cu 2p spectrum, the blue‐shift phenomenon relative to Cu/OC was still seen. Besides intermetallic interaction, the graphene chainmail also facilitates free electron transfer, demonstrated in the O 1s and C 1s spectra. The O 1s spectra can be subdivided into four contributing peaks: lattice oxygen (≈529.8 eV, O_α_), oxygen vacancy (≈531.7 eV, O_β_), surface adsorbed oxygen (≈533.3 eV, O_γ_), and adsorbed water (≈536.5 eV, O_δ_).^[^ ^32^ ^]^ Compared with Cu/OC, O_α_ and O_β_ in Cu–Ni bimetallic catalysts showed a red shift which may be attributed to the electronic interaction between the dilute CuNi alloy and the carbon shell. CuNi_0.05_@OC possessed the highest proportion of O_β_, conducive to increasing catalytic activity by modulating surface and electronic structure of the catalysts,^[^ ^33^ ^]^ which was further confirmed by ESR analysis (Figure 3e). The clear ESR signal at *g* = 2.003, allocated to oxygen vacancies,^[^ ^34^ ^]^ showed the strongest signal intensity for CuNi_0.05_@OC, suggesting the presence of more unpaired electrons.^[^ ^35^ ^]^ In the C 1s spectra, the major peak can be deconvolved into five sub‐peaks of C═O, C─O, C─C, C═C, and C─M with descending binding energies from 289 to 284 eV.^[^ ^36^ ^]^ The CuNi_0.05_@OC sample displayed the highest ratio of C─M area with the most red‐shift of this peak, indicating the strongest interaction between confined dilute Ni alloy and graphene shell.^[^ ^37^ ^]^ Similarly, the peak at 289 eV belonging to the C═O bonding obviously shifts to lower BE in bimetallic CuNi*_x_* @OC compared with the single metal counterparts.^[^ ^38^ ^]^

H_2_‐TPR analysis (Figure 3f) was used to study interaction of the samples. Addition of Ni gradually decreased the onset reduction peak temperature from 192.5 °C of Cu/OC to 179.1 °C for CuNi_0.05_@OC and 164.4 °C for CuNi_0.25_@OC, owning to hydrogen spilling from Ni over Cu*_x_* O*_y_*, which are also evidenced by the temperature decrease of the inverted peaks.^[^ ^39^, ^40^ ^]^ CuNi_0.05_@OC showed the widest asymmetric reduction peak, likely attributed to the dilute alloying effect that promotes dissociation and chemisorption of hydrogen.^[^ ^41^, ^42^ ^]^ The high Ni content CuNi_0.65_@OC sample showed a peak around 350 °C related to Ni oxide in a separated phase (Figure S9, Supporting Information), consistent with its asymmetric shoulder peak of (111) facet detected by XRD. This implies that Ni content shows an optimal value with regard to the most desirable interaction. The response of the dilute alloy to the dissociation ability of H species can be better known from H_2_‐TPD and XPS valence band (VB) spectra. As shown in Figure 3g, the ≈600 °C desorption peak can be attributed to the hydrogen overflow effect,^[^ ^35^ ^]^ and the main peak positions are the same as those from H_2_‐TPR. The onset and peak hydrogen desorption temperatures of CuNi_0.05_@OC catalyst were obviously lower than those of Cu/OC, indicating that trace Ni significantly enhances hydrogen desorption, and promotes hydrogenation kinetics. Ni doping gives CuNi_0.05_@OC an estimated VB maximum value of ≈1.93 eV, much higher than that of ≈1.11 eV for Cu/OC (Figure 3h). The dilute Ni alloying noticeably pushed the valence band, and thus the d‐band center away from Fermi level, which further implies that CuNi_0.05_@OC has stronger hydrogen desorption capability thanks to the depressed antibonding energy state.^[^ ^43^ ^]^

**Figure 4:**
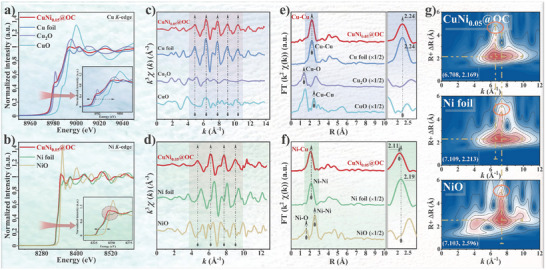
XAS spectra analysis of CuNi_0.05_@OC: a,b) Cu or Ni K‐edge XANES spectra; c,d) Corresponding k‐spaced EXAFS spectra; e,f) Cu or Ni K‐edge FT‐EXAFS spectra; g) The EXAFS wavelet transform spectra of CuNi_0.05_@OC, Ni foil, and NiO, respectively.

The atomic and electronic structure of CuNi_0.05_@OC was further probed with X‐ray absorption spectroscopy (XAS). As shown in **Figure** **4**a,b, the normalized X‐ray absorption near‐edge structure (XANES) spectra of Cu K‐edge and Ni K‐edge indicated the near metallic nature of Cu and Ni in CuNi_0.05_@OC.^[^ ^44^ ^]^ The average metal valences in the dilute CuNi_0.05_ alloy are both estimated to be ≈0, obtained from the first derivative of the XANES spectra and the corresponding fitted curves (Figures S10 and S11, Supporting Information), which was further confirmed by the Cu L_3_VV Auger spectra (Figure S12 and Table S5, Supporting Information). The significantly higher Cu° content in CuNi_0.05_@OC versus Cu@OC further evidences that the doped Ni atoms stabilize zero valence Cu species. The high intensity white line in CuNi_0.05_@OC may indicate singly dispersed Ni atoms in Cu host.^[^ ^45^ ^]^ The K_3_‐weighted Fourier‐transform extended X‐ray absorption fine structure (FT‐EXAFS) spectrum of CuNi_0.05_@OC is close to that of Cu foil, but distinct from Ni foil in k‐space (Figure 4c,d). In R‐space, CuNi_0.05_@OC displays a main peak at ≈2.24 Å attributable to the Cu─Cu bond with absence of Cu─O bond (Figure 4e), but a strong peak from the first coordination shell of Ni with slightly different position from Ni foil (Figure 4f). The corresponding wavelet transform (WT) analysis (Figure S13, Supporting Information) shows well crystallized Cu particles in CuNi_0.05_@OC in an ordered metallic Cu lattice, consistent with the XRD and HR‐TEM results. The fitted Cu─Cu coordination number for CuNi_0.05_@OC was ≈7, lower than that of 12 for Cu foil (Figure S14 and Table S4, Supporting Information), implying the presence of abundant coordination‐unsaturated Cu sites on the sample surface, which can act as the active phase during hydrogen overflow.^[^ ^46^ ^]^ Combining the apparent change in the k‐space representation of Ni and the high resolution deconvolution results of CO‐DRIFTS (Figure S15, Supporting Information), Ni atoms in the dilute alloy CuNi_0.05_ are isolated and only bond with Cu atoms to form Ni─Cu bond.^[^ ^47^ ^]^ Due to the close Ni─Cu, Ni─Ni, and Cu─Cu scattering paths, the WT analysis of CuNi_0.05_@OC (Figure 4g) exhibits an intensity distribution profile similar to those of metal‐foils, but with an intensity maximum at ≈6.7 Å^−1^ attributed to the Ni─Cu, distinct from that of ≈7.1 Å^−1^ assigned to Ni─Ni contribution. According to the EXAFS data fit (Figure S16 and Table S4, Supporting Information), the Ni─Cu coordination bond length of 2.41 Å was smaller than that of Ni─Ni bond (2.46 Å) or Cu─Cu bond (2.53 Å), and the Ni─Cu coordination number of ≈8.6 was below those in typical FCC body and surface (111) with coordination numbers of 12 and 9, confirming singularly dispersed Ni on the Cu host containing dangling bonds.^[^ ^45^, ^48^ ^]^ No Ni─Ni and Ni─O coordination could be detected.

### 2.3 Catalytic Performance

The catalytic transfer hydrogenation (CTH) of nitroaromatics to produce the corresponding amines are important in organic chemical production which turns waste to goods, and the reactions under mild conditions are highly desirable due to the environmental friendliness.^[^ ^49^ ^]^ In this study, CTH of *p*‐CNB to *p*‐CAN in aqueous phase was chosen as the first model reaction to evaluate the catalytic performance of the various catalysts. As listed in **Table** **1**, CuNi_0.05_@OC clearly stood out among bimetallic catalysts (entries 3–6), displaying the highest *p*‐CAN selectivity of 99.4% and excellent *p*‐CNB conversion of 97.9% (entry 4), which was kept the same even at scaled up reaction (entry 9), outperforming the monometallic Cu/OC or Ni@OC (entries 1–2), their physical mixture (entry 7), and the commercial Pd/C (entry 8), suggesting that Ni atoms in the dilute alloy provide the key catalytic active sites. Consistent with previous reports, Cu is a highly selective hydrogenation metal,^[^ ^32^ ^]^ but it has low activity for *p*‐CNB hydrogenation. Obviously, there was a close synergy between Cu and dilute Ni in promoting hydrogenation, so that CuNi_0.05_@OC catalyst gave a turnover frequency (TOF) of 45.19 h^−1^ based on Ni and 1.12 h^−1^ based on Cu, several times higher than those of 4.02 and 0.21 h^−1^ for single‐metal Ni@OC and Cu/OC, respectively (Figure S17, Supporting Information).

**Table 1:** CTH of *p*‐CNB over different catalysts.

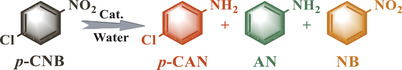					
Entry^a)^	Cat.^b)^	Conv. [%]^c)^	Sel. [%]^c)^		
			*p*‐CAN	AN	NB
1	Cu/OC	27.7	99.3	0.7	—
2	Ni@OC	40.2	87.1	1.8	11.1
3	CuNi_0.01_@OC	56.5	96.2	3.1	0.7
4	CuNi_0.05_@OC	97.9	99.4	0.5	0.1
5	CuNi_0.25_@OC	99.8	88.7	10.4	0.9
6	CuNi_0.65_@OC	73.2	81.3	15.6	3.1
7^d)^	Cu/OC + Ni@OC	53.8	73.9	17.1	9.0
8^e)^	Pd/C	[72.1; 69.1]	[10.1; 13.6]	[88.7; 83.6]	[1.2; 2.8]
9^f)^	CuNi_0.05_@OC	96.0	99.7	0.3	—
10^g)^	blank	n.d.^h)^	—	—	—

^a)^ Reaction conditions: hydrazine hydrate (1.5 mmol); catalyst (20 mg); *p*‐CNB (0.5 mmol); water (15 mL); *T* = 50 °C; 4 h; 1000 rpm

^b)^ The actual metal loadings were determined by ICP‐AES

^c)^ The conversion of *p*‐CNB and selectivity toward the products were determined by GC─MS

^d)^ Physical mixture of Cu/OC and Ni@OC with Cu/Ni ratio of 20

^e)^ Commercial Pd/C (the corresponding values are with respect to the use of 5.0 and 10.0 wt% of Pd, respectively)

^f)^ Scale‐up test with *p*‐CNB (1.5 mmol)

^g)^ No catalyst

^h)^ Not detected.

**Figure 5:**
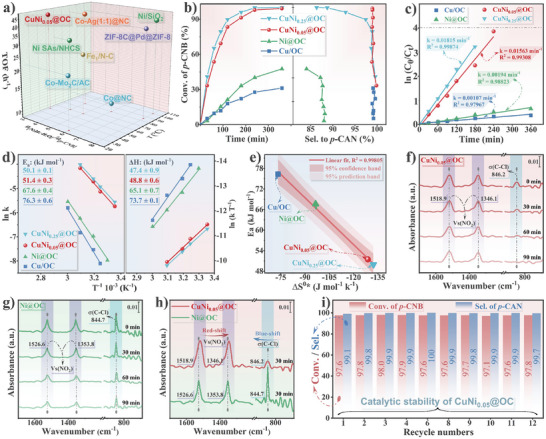
a) Comparison of CTH of *p*‐CNB over different catalysts (see Table S6, Supporting Information, for details). Catalytic performance of CuNi_0.05_@OC compared with Cu/OC, CuNi_0.25_@OC, and Ni@OC: b) Kinetic profiles. c) Plot of ln(*C*_0_/*C_t_*) versus time. d) Arrhenius plots and Eyring plots. e) Plot of activation energy (E_a_) against entropy change (ΔS^0*^). f–h) In situ DRIFT spectra concerning competitive adsorption of chlorobenzene and nitrobenzene. i) Reusability test with CuNi_0.05_@OC. Reaction conditions: hydrazine hydrate (1.5 mmol); *p*‐CNB (0.5 mmol); catalyst (20 mg); water (15 mL); T = 50 °C; 4 h; 1000 rpm.

Compared with literature reported catalysts, CuNi_0.05_@OC also has outstanding activity (**Figure** **5**a and Table S6, Supporting Information). Further kinetic analysis showed obviously higher reaction rate constant using CuNi_0.05_@OC than those of monometallic Cu/OC and Ni@OC catalysts (Figure 5b,c), which was similar to that using CuNi_0.25_@OC with fivefold nickel content showing poorer *p*‐CAN selectivity. The activation energy (*E*_a_) and activation enthalpy (Δ*H*) over CuNi_0.05_@OC, ≈50 kJ mol^−1^ based on effects of reaction temperature on the hydrogenation rate (Figure S18, Supporting Information), were close to those of CuNi_0.25_@OC, much lower than those >65 kJ mol^−1^ over Cu/OC and Ni@OC (Figure 5d and Table S7, Supporting Information), demonstrating the strong influence of surface Ni atoms on the H species adsorption, and in turn on the catalytic activity. The excellent linear relationship between *E*_a_ and activation entropy (Δ*S* ^0^*) (Figure 5e) confirms the compensation effect likely due to loosened bonding between surface atoms and adsorbates as the system energy increases.^[^ ^48^, ^50^ ^]^ To further explain the high chemoselectivity of CuNi_0.05_@OC, we implemented in situ DRIFT for competitive adsorption of chlorobenzene and nitrobenzene on CuNi_0.05_@OC and Ni@OC, respectively, as shown in Figure 5f,g. The DRIFTS for CuNi_0.05_@OC include characteristic bands located at 1518.9 and 1346.1 cm^−1^ assigned to the N═O bond of nitrobenzene, and at 846.2 cm^−1^ corresponding to the C─Cl bond of chlorobenzene in the vibrational band.^[^ ^51^ ^]^ When purged with Ar gas, the signal of nitrobenzene remained essentially constant, while the peak associated with C─Cl gradually fades away, indicating CuNi_0.05_@OC prefers N═O adsorption, which can be further confirmed by the relative shift of the peaks (Figure 5h).^[^ ^52^ ^]^ For Ni@OC, C─Cl adsorbs more strongly as the peak at 844.7 cm^−1^ is much stronger than the other two peaks associated with the N═O bond.

Easy separation and recoverability are the key factors for practical application of the catalysts. A heterogeneity experiment proves that the reaction proceeded in a non‐homogeneous manner (Figure S19, Supporting Information). Dispersed Ni addition endows the Cu─Ni alloy with magnetism, thus CuNi_0.05_@OC synthesized at room temperature showed saturation magnetization of 20.5 emu g^−1^ (Figure S20, Supporting Information), which makes catalyst recovery easier and more efficient as shown in Movie S1 and Figure S21, Supporting Information. For the repeatedly usage, the stability of CuNi_0.05_@OC (Figure 5i) was obviously better than that of Cu/OC (Figure S22, Supporting Information). The excellent yield toward *p*‐CAN >97% using CuNi_0.05_@OC can be maintained in the continuous hydrogenation even after 12 cycles, while using Cu/OC, the *p*‐CNB conversion decreased almost half from 27.6% to 14.8% after seven runs. Furthermore, we also conducted recycling tests under kinetically controlled conditions.^[^ ^53^ ^]^ Briefly, we comparatively assessed the TOF values in the recycle runs with CuNi_0.05_@OC and Cu/OC catalysts in the hydrogenation of *p*‐CNB in water. As can be seen from Table S8, Supporting Information, CuNi_0.05_@OC exhibited consistently high TOF values measured in six runs with magnetic recovery of the catalyst, which demonstrated satisfactory stability. The sharp difference in stability can be well explained by the structure changes during hydrogenation, as compared in XRD patterns (Figure S23, Supporting Information), nitrogen adsorption–desorption isotherms (Figure S24, Supporting Information), TEM images (Figure S25, Supporting Information), and HR‐XPS spectra (Figure S26, Supporting Information). The used CuNi_0.05_@OC exhibited almost identical phase and morphological features as fresh ones, but the used Cu/OC displayed obvious copper oxide peaks in XRD, a loss of specific surface area, and significantly coarsening of the Cu NPs. Further analysis with ICP‐AES (Table S1, Supporting Information) revealed obvious Cu leaching from Cu/OC during hydrogenation lowering the Cu content from the initial 58.6 to 41.9 wt%, while that of CuNi_0.05_@OC remained unchanged at 56.6–58.1 wt%. The trace Ni doping and the tightly wrapping graphene chainmail protected Cu against sintering and oxidation, which stabilize CuNi_0.05_@OC during consecutive hydrogenation cycles. Further, we evaluated the stability of CuNi_0.05_@OC in air. As shown in Figure S27, Supporting Information, even after exposure to air for 6 days, it still affords excellent catalytic performance.

### 2.4 Mechanistic Insights and Theoretical Calculations

The systematic analysis of the CuNi*_x_* @OC series including Cu/OC, CuNi_0.01_@OC, CuNi_0.05_@OC, CuNi_0.25_@OC, CuNi_0.65_@OC, and Ni@OC with different composition could provide insight into structure–activity relationship of the catalyst. When used for catalyzing CTH of *p*‐CNB, with the increasing Ni content in the series catalysts, the *p*‐CAN yield first increased and then decreased almost linearly. Among the series catalysts, CuNi_0.05_@OC displayed the highest *p*‐CAN yield, indicating an optimal Ni content for promoting performance (Figure S28a, Supporting Information). The volcano shaped catalytic performance correlated well with the trend of lattice strain (Figure 3a), C‐metal binding (Figure S28b, Supporting Information), surface oxygen vacancy (Figure S28c, Supporting Information), surface defect carbon (Figure S28d, Supporting Information), but different from the surface area (Figure S28e, Supporting Information), porosity (Figure S28f, Supporting Information), and the number of graphene layers of the samples (Figure S4, Supporting Information). Thus, for the catalytic activity, electronic effect including bimetallic synergy and Mott–Schottky heterojunction contributed significantly to the enhanced performance, while the porous structure was not the critical factor. On the other hand, from the performance of recycled catalysts CuNi_0.05_@OC and Cu/OC (Figure 5i and Figure S22, Supporting Information), it can be deduced that the porous carbon structure plays a vital role in preserving catalytic activity in consecutive reactions. Therefore, the pore structure positively promoted catalyst stability.

**Figure 6:**
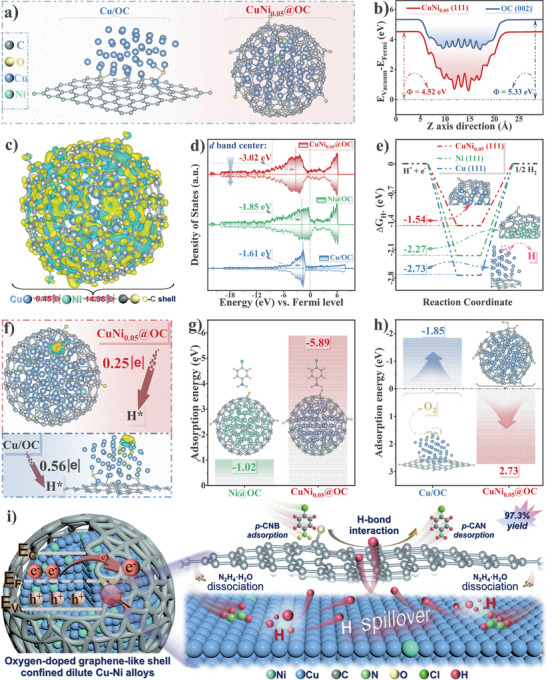
Theoretical calculations: a) Geometric configurations of Cu (111) and CuNi_0.05_ (111) surfaces. b) Calculated work function (φ) of CuNi_0.05_(111) and OC (002) shell. c) Charge density differential of CuNi_0.05_ (111) (the yellow and blue regions indicate electron increment and depletion, respectively). d) PDOS of d‐bands for Cu/OC, Ni@OC, and CuNi_0.05_@OC. e) Free energies of H adsorption on Cu (111), Ni (111), and CuNi_0.05_ (111). f) Side view of differential charge density diagrams of H‐adsorption state for Cu/OC and CuNi_0.05_@OC models. g) Adsorption energy of *p*‐CNB on the surface of Ni@OC and CuNi_0.05_@OC. h) Adsorption energy of oxygen on the surface of Cu/OC and CuNi_0.05_@OC. i) Proposed mechanism of *p*‐CNB hydrogenation over CuNi_0.05_@OC.

To clarify how the dilute Cu─Ni alloying and the oxygen‐doped carbon chainmail affect the hydrogenation reaction, DFT calculations were performed. Based on the experimentally measured structural features of CuNi_0.05_@OC, we selected the (111) plane of dilute CuNi alloy core and the (002) plane of OC shell, in comparison with Cu/OC modeled by Cu (111) on OC (002) (**Figure** **6**a) and Ni@OC containing Ni (111) in OC (002) (Figure S29, Supporting Information). Employing Ni (111) with moderate work function of *Φ* = 4.69 eV (Figure S30, Supporting Information) as pseudo shell of the CuNi_0.05_ alloy shifts its work function to lower value of 4.52 eV (Figure 6b). Therefore, the electron transfer from such CuNi_0.05_ alloy (111) to OC (002) with large *Φ* = 5.33 eV is facilitated, as assessed by Kelvin probe force microscopy.^[^ ^54^ ^]^ Figure S31, Supporting Information, shows that the surface potential of OC was higher than that of CuNi_0.05_, and the apparent potential difference (≈19 mV) indicates a built‐in electric field from OC to CuNi_0.05_ at their interface, that is, electrons can move from CuNi_0.05_ to OC. The differential charge density analysis of CuNi_0.05_@OC (Figure 6c) graphically shows electron migration tendency from the inner metallic sphere to outer carbon chainmail. Further Bader charge analysis of CuNi_0.05_@OC estimates that the amount of charge transferred from the internal Cu─Ni dilute alloy to the outer OC shell is 14.96 |e|, and the isolated Ni atom receives 0.45 |e| from sub‐surface Cu atoms (Figure S32, Supporting Information), in line with the XPS and in situ CO‐DRIFTS results. The density of states (DOS) around the Fermi level of the investigated model and their corresponding d‐band centers are plotted in Figure 6d. The d‐band centers of CuNi_0.05_@OC, −3.02 eV, is farther away from *E*_F_ compared to the single metallic cases, indicating a decrease in the antibonding energy state and weakening of the BE between catalyst and adsorbate,^[^ ^55^, ^56^ ^]^ which confirms the aforementioned “compensation effect” and VB results. Therefore, the close contact between the dilute CuNi*_x_* alloy and OC leads to metal–support interfaces analogue to Mott–Schottky heterojunction triggering electron redistribution until their *E*_F_ reach equilibrium and a depletion layer will be formed around the metal (Figure S33, Supporting Information).^[^ ^20^ ^]^

Calculated hydrogen dissociation free energies (Δ*G*_H_*) on CuNi_0.05_@OC of −1.54 eV is much lower in absolute value than those of −2.27 eV on Ni@OC and −2.73 eV on Cu/OC (Figure 6e), indicating easier hydrogen desorption from the (111) plane of the Cu─Ni alloy than from the pure Ni or Cu surfaces.^[^ ^26^, ^43^ ^]^ A rough linear negative correlation between the d band center and the Δ*G*_H_* value (Figure S34, Supporting Information) further confirms the depressed antibonding state.^[^ ^43^ ^]^ Correspondingly, a smaller electron cloud forms between the adsorbed H atom and CuNi_0.05_@OC (Figure 6f) which would facilitate desorption of H intermediates from the catalyst surface. The corresponding charge analysis shows decreased CuNi─H* binding by CuNi_0.05_@OC with smaller number of transferred electrons (0.25 |e|) between CuNi and H* intermediates compared to 0.56 |e| of Cu/OC (Figure S35, Supporting Information). For the substrate of *p*‐CNB, CuNi_0.05_@OC displays much higher adsorption energy (Figure 6g) and shorter adsorption distance (Figure S36, Supporting Information) than Ni@OC, indicating that *p*‐CNB molecules are more prone to access CuNi_0.05_@OC surface for enhanced enrichment.^[^ ^57^ ^]^ These synergistically all lead to excellent activation of *p*‐CNB. The excellent selectivity can be explained by preferred perpendicular activation of ─NO_2_ on the CuNi_0.05_@OC surface, reflected by the increased ─NO_2_ bond lengths from initial 1.236 to 1.237 and 1.418 Å for Ni and Cu─Ni activated *p*‐CNB, respectively, and the accompanied bond angle change (Figure S37, Supporting Information).^[^ ^58^ ^]^ Figure 6h compares the response to oxygen molecules in the corresponding models, with CuNi_0.05_@OC exhibiting remarkably lower adsorption energies than Cu/OC, confirming the protective role of the oxygen‐doped graphene shell against oxidative attack to the vulnerable transition metal core. This improves the long‐term stability of the CuNi_0.05_@OC catalysts.

From the above analysis, the mechanism for improved hydrogenation performance including activity, selectivity, and stability, with dilute alloy chainmail catalyst CuNi_0.05_@OC is proposed, as shown in Figure 6i for the CTH of *p*‐CNB to *p*‐CAN. Crucial factors determining the activity and selectivity including metal–support interaction, atomically isolated active sites, alloy effect, strain effect, and defects have been properly manipulated on CuNi_0.05_@OC catalyst which possesses dispersed Ni sites,^[^ ^59^ ^]^ the highest lattice compressive strain, abundant carbon defects, and the most desirable three layers of oxygen‐doped graphene shell. A positive correlation of TOF values with lattice compressive strain (*τ*) and the proportion of O_β_ (Figure S38, Supporting Information) suggest that the rich strain and oxygen vacancy in CuNi_0.05_@OC could increase active H species and promote hydrogen escape from the catalyst surface.^[^ ^35^ ^]^ Due to the higher bond energy of Ni─H than Cu─H, the electron‐rich hydrazine dissociates easily on the Ni sites.^[^ ^60^ ^]^ The generated active H species on Ni atom spill over to the nearby Cu surface which can be regarded as a reservoir for storing active H species, rendering the latter active for hydrogenation reactions. The carbon shell with higher surface area and rich pores has high adsorption capacity for *p*‐CNB.^[^ ^7^, ^61^, ^62^ ^]^ The close Schottky contact between the dilute alloy nanoparticles and few‐layer graphene shell increases the local charge density, and finally promotes the efficient electron flow from catalyst surface to the adsorbed *p*‐CNB molecules.^[^ ^63^ ^]^ The electron‐rich H tends to participate nucleophilic attack on ─NO_2_ of *p*‐CNB, rather than electrophilic attack on C─Cl bond.^[^ ^64^ ^]^ Due to the electron‐donating effect of ─NH_2_, the repulsion of electron‐rich carbon shell to C─Cl on *p*‐CAN promotes adsorption of nitro substrates and desorption of aniline products in a synergistic way, which inhibits C─Cl cracking,^[^ ^65^ ^]^ giving rise to high *p*‐CAN selectivity. Thus, the hydrogenation of *p*‐CNB over chainmail dilute alloy catalyst shows a synergistic division mechanism. Namely, the atomically dispersed Ni species dissociate hydrazine molecules and overflow activated H species to the nearby Cu site, while the OC shell is responsible for the preferential adsorption of *p*‐CNB. Meanwhile, the transfer of hydrogen atoms from metal site to the outer OC occurs successively to facilitate *p*‐CNB reduction. Insight into the response of OC to hydrogen overflow can be gained from controlled experiments using WO*_3_ as an indicator. As shown in Figure S39, Supporting Information, color of the catalyst and substrate mixture changes from light yellow to light blue with the involvement of WO_3_, indicating that hydrogen spillage has occurred, as the spilled hydrogen migrates and readily reacts with the yellow WO_3_ to form blue H*_x_* WO_3_.^[^ ^66^ ^]^

### 2.5 Catalytic Hydrogenation Universality of CuNi_0.05_@OC

**Figure 7:**
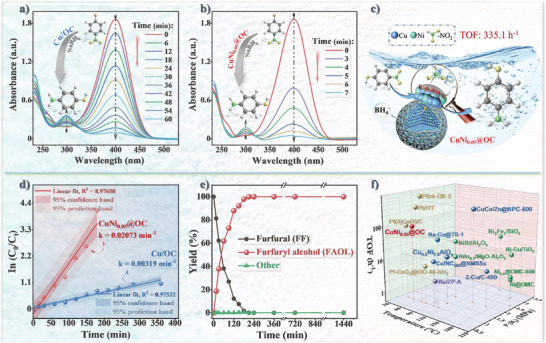
The time‐dependent UV–vis absorption spectra following the *p*‐NP reduction reaction over a) Cu/OC and b) CuNi_0.05_@OC. c) The possible reduction process of *p*‐NP on the surface of dilute CuNi_0.05_ alloy NPs. Reaction conditions: NaBH_4_ (0.2 m); *p*‐NP (10 mm); catalyst (2 mg); 25 °C; 1000 rpm. SH of FF toward FAOL over Cu/OC and CuNi_0.05_@OC: d) Plot of ln (*C*_0_/*C_t_*) versus time. e) Evolution of the product distribution with reaction time over CuNi_0.05_@OC. f) Comparison of the performance of hydrogenation over different catalysts (see Table S12, Supporting Information, for details). Reaction conditions: P(H_2_) (1.0 MPa); FF (0.75 mmol); catalyst (20 mg); water (15 mL); 50 °C; 800 rpm.

Besides CTH of *p*‐CNB, CuNi_0.05_@OC also showed excellent catalytic performance in hydrogenation of a rich variety of functionalized nitroaromatics, giving rise to primary amine compounds in almost 100% yields (Table S9, Supporting Information). Additionally, using sodium borohydride as the hydrogen source, we have systematically investigated another set of CTH conversion including *p*‐NP to *p*‐AP, a class of important intermediates for rubber, dye, pesticide, and medicine synthesis,^[^ ^62^ ^]^ with a series of designed experiments. While the OC shell only acts as adsorbent for *p*‐NP (Figure S40a, Supporting Information), CuNi_0.05_@OC prompted faster conversion of *p*‐NP to *p*‐AP, represented by UV peaks at 400 and 295 nm respectively, compared to Cu/OC (**Figure** **7**a,b). The linear correlation between ln (*C*_0_/*C_t_*) and *t* indicates pseudo‐first order kinetics (Figure S40b, Supporting Information) with the apparent rate constant of 0.539 min^−1^ for CuNi_0.05_@OC significantly larger than that of 0.048 min^−1^ for Cu/OC. The high TOF value of 335.1 h^−1^ achieved using CuNi_0.05_@OC under mild reaction conditions surpasses many reported catalysts (Figure S40c and Table S10, Supporting Information), thanks to the synergistic effect of dilute Cu─Ni alloying and oxygen‐doped carbon chainmail (Figure 7c).^[^ ^67^ ^]^ In order to fully evaluate its potential in hydrogenation reactions, CuNi_0.05_@OC was also used in the selective hydrogenation (SH) of FF in the presence of hydrogen gas. FF is one of the most important biomass‐based platform compounds, its SH into FAOL receives growing attention as greener renewable replacement of oil‐based chemicals in various industries.^[^ ^68^ ^]^ However, due to diverse unsaturated groups in the molecule, effective and selective FF hydrogenation toward FAOL is still a huge challenge often resulting in slow reaction and mixture products (Figure S41, Supporting Information). As summarized in Table S11, Supporting Information, both Cu/OC and CuNi_0.05_@OC achieved ≈100% FAOL selectivity under the same conditions, while the latter shows a rate constant six times higher than the former (Figure 7d). The high selectivity toward FAOL does not attenuate with extended reaction time even after 24 h (Figure 7e). Compared to the preferred catalysts reported in the literature, the CuNi_0.05_@OC catalyst displayed superior catalytic SH performance of FF toward FAOL under low reaction temperature and pressure (Figure 7f and Table S12, Supporting Information). The catalytic SH performances of CuNi_0.05_@OC to convert other biomass‐derived aldehydes, including 2‐naphthaldehyde, 5‐hydroxymethylfurfural, and benzaldehyde, into alcohols are all remarkable with nearly 100% conversion and >92.6% selectivity (entries 2–7, Table S11, Supporting Information). The above showcases demonstrate the huge application potential of the chainmail dilute alloy CuNi_0.05_@OC catalyst as a cost‐effective and versatile reaction platform for organic conversion under mild reaction conditions.

## 3 Conclusion

In conclusion, a facile one‐pot strategy has been developed for preparation of graphene chainmail protected dilute alloy catalyst via pyrolysis of bimetallic MOF with sharp contrast carbon solubility. Judicious selection of fabrication process yields an optimal three layers of oxygen‐doped graphene shell and dilute CuNi_0.05_ core with large compressive strain and carbon defects. The dual benefit of dispersed Ni on Cu base and outer OC chainmail include successive electron transfer from sub‐Cu to OC via dispersed Ni and anti‐oxidation protection, which leads to simultaneous achievements of excellent activity, selectivity, and durability during catalysis. The CuNi_0.05_@OC catalysts in this study achieve impressive catalytic performance for the transfer hydrogenation of *p*‐CNB to *p*‐CAN using hydrazine hydrate, reduction of nitro aromatics with sodium borohydride, and SH of FF employing hydrogen gas, all in aqueous media, outperforming most supported metal catalysts reported in literature. The broad tolerance of various nitroaromatics and unsaturated aldehydes, as well as rich hydrogen donors demonstrate excellent universality of the chainmail dilute CuNi*_x_* alloy, delivering easily scalable production of non‐noble metal based catalysts for practical hydrogenation applications.

## Conflict of Interest

The authors declare no conflict of interest.

## Author Contributions

H.Y.: Conceptualization, Methodology, Investigation, Data and formal analysis, Visualization, Writing—original draft, Writing—review and editing. M.H.: Data and formal analysis, Data curation, Funding acquisition, Conceptualization, Supervision, Writing—review and editing. X.H., W.Q., and F.D.: Investigation, Data and formal analysis, Visualization. Y.Z. and Y.C.: Formal analysis, Data curation. J.G.: Formal analysis, Writing—review and editing. S.Y.: Formal analysis, Project administration, Funding acquisition, Conceptualization, Supervision, Writing—review and editing. All authors discussed the results, drew conclusions, and commented on the manuscript.

## Supporting information

Supporting Information

Supplemental Movie 1

## Data Availability

The data that support the findings of this study are available from the corresponding author upon reasonable request.

## References

[1] C. Vogt , B. M. Weckhuysen , Nat. Rev. Chem. 2022, 6, 89. 37117296 10.1038/s41570-021-00340-y

[2] L. Zhang , M. Zhou , A. Wang , T. Zhang , Chem. Rev. 2020, 120, 683. 31549814 10.1021/acs.chemrev.9b00230

[3] X. Liu , G. Lan , Z. Li , L. Qian , J. Liu , Y. Li , Chin. J. Catal. 2021, 42, 694.

[4] L. Yu , D. Deng , X. Bao , Angew. Chem., Int. Ed. 2020, 59, 15294. 10.1002/anie.202007604 32473050

[5] X. Cui , P. Ren , D. Deng , J. Deng , X. Bao , Energy Environ. Sci. 2016, 9, 123.

[6] S. Gutiérrez‐Tarriño , S. Rojas‐Buzo , C. W. Lopes , G. Agostini , J. J. Calvino , A. Corma , P. Oña‐Burgos , Green Chem. 2021, 23, 4490.

[7] W. Yan , F. Xiao , X. Li , W. He , Y. Yao , D. Wan , X. Liu , Y. Liu , F. Feng , Q. Zhang , C. Lu , X. Li , Chem. Eng. J. 2023, 452, 139361.

[8] L. Zhao , X. Qin , X. Zhang , X. Cai , F. Huang , Z. Jia , J. Diao , D. Xiao , Z. Jiang , R. Lu , N. Wang , H. Liu , D. Ma , Adv. Mater. 2022, 34, 2110455. 10.1002/adma.202110455 35305275

[9] T. Yang , X. Mao , Y. Zhang , X. Wu , L. Wang , M. Chu , C.‐W. Pao , S. Yang , Y. Xu , X. Huang , Nat. Commun. 2021, 12, 6022. 34654822 10.1038/s41467-021-26316-6 PMC8519910

[10] S. Chen , R. Wojcieszak , F. Dumeignil , E. Marceau , S. Royer , Chem. Rev. 2018, 118, 11023. 30362725 10.1021/acs.chemrev.8b00134

[11] J. Vavra , T.‐H. Shen , D. Stoian , V. Tileli , R. Buonsanti , Angew. Chem., Int. Ed. 2021, 60, 1347. 10.1002/anie.202011137 32997884

[12] M. T. Marques , J. B. Correia , O. Conde , Scr. Mater. 2004, 50, 963.

[13] T. Li , Y. Bai , Y. Wang , H. Xu , H. Jin , Coord. Chem. Rev. 2020, 410, 213221.

[14] F. Lan , H. Zhang , C. Zhao , Y. Shu , Q. Guan , W. Li , ACS Catal. 2022, 12, 5711.

[15] J.‐M. Aguiar‐Hualde , Y. Magnin , H. Amara , C. Bichara , Carbon 2017, 120, 226.

[16] R. Yang , J. Catal. 1990, 122, 206.

[17] J. Wu , J. Fan , X. Zhao , Y. Wang , D. Wang , H. Liu , L. Gu , Q. Zhang , L. Zheng , D. J. Singh , X. Cui , W. Zheng , Angew. Chem., Int. Ed. 2022, 61, e202207512. 10.1002/anie.202207512 35762984

[18] M. A. Ahsan , A. R. P. Santiago , Y. Hong , N. Zhang , M. Cano , E. Rodriguez‐Castellon , L. Echegoyen , S. T. Sreenivasan , J. C. Noveron , J. Am. Chem. Soc. 2020, 142, 14688. 32786805 10.1021/jacs.0c06960

[19] Z. Xia , S. Guo , Chem. Soc. Rev. 2019, 48, 3265. 31089609 10.1039/c8cs00846a

[20] R. G. Rao , R. Blume , M. T. Greiner , P. Liu , T. W. Hansen , K. S. Dreyer , D. D. Hibbitts , J.‐P. Tessonnier , ACS Catal. 2022, 12, 7344.

[21] W. Zhang , X. Mei , L. Yuan , G. Wang , Y. Li , S. Peng , Appl. Surf. Sci. 2022, 593, 153459.

[22] S. Luo , H. Song , D. Philo , M. Oshikiri , T. Kako , J. Ye , Appl. Catal. B 2020, 272, 118965.

[23] J. Yu , Y. Yang , L. Chen , Z. Li , W. Liu , E. Xu , Y. Zhang , S. Hong , X. Zhang , M. Wei , Appl. Catal. B 2020, 277, 119273.

[24] Q. Chen , P. Peng , G. Yang , Y. Li , M. Han , Y. Tan , C. Zhang , J. Chen , K. Jiang , L. Liu , C. Ye , E. Xing , Angew. Chem., Int. Ed. 2022, 61, e202205978. 10.1002/anie.202205978 35679132

[25] W. Li , Y. Zhao , Y. Liu , M. Sun , G. I. N. Waterhouse , B. Huang , K. Zhang , T. Zhang , S. Lu , Angew. Chem., Int. Ed. 2021, 60, 3290. 10.1002/anie.202013985 33105050

[26] Z. Jiang , S. Song , X. Zheng , X. Liang , Z. Li , H. Gu , Z. Li , Y. Wang , S. Liu , W. Chen , D. Wang , Y. Li , J. Am. Chem. Soc. 2022, 144, 19619. 36223550 10.1021/jacs.2c09613

[27] Z.‐D. Yang , X.‐Y. Yang , T. Liu , Z.‐W. Chang , Y.‐B. Yin , X.‐B. Zhang , J.‐M. Yan , Q. Jiang , Small 2018, 14, 1800590. 10.1002/smll.201800590 30047210

[28] J. Chen , J. Feng , F. Yang , R. Aleisa , Q. Zhang , Y. Yin , Angew. Chem., Int. Ed. 2019, 58, 9275. 10.1002/anie.201904828 31062923

[29] M. Kan , C. Yang , Q. Wang , Q. Zhang , Y. Yan , K. Liu , A. Guan , G. Zheng , Adv. Energy Mater. 2022, 12, 1614.

[30] M. Komarneni , J. Shan , U. Burghaus , J. Phys. Chem. 2011, 115, 16590.

[31] M. J. Islam , M. G. Mesa , A. Osatiashtiani , J. C. Manayil , M. A. Isaacs , M. J. Taylor , S. Tsatsos , G. Kyriakou , Appl. Catal. B 2021, 299, 120652.

[32] Z.‐F. Huang , S. Xi , J. Song , S. Dou , X. Li , Y. Du , C. Diao , Z. J. Xu , X. Wang , Nat. Commun. 2021, 12, 3992. 34183651 10.1038/s41467-021-24182-w PMC8238955

[33] S. Zhao , Y. Yang , F. Bi , Y. Chen , M. Wu , X. Zhang , G. Wang , Chem. Eng. J. 2023, 454, 140376.

[34] C. Miao , T. Hui , Y. Liu , J. Feng , D. Li , J. Catal. 2019, 370, 107.

[35] Z. Li , W. Wei , H. Li , S. Li , L. Leng , M. Zhang , J. H. Horton , D. Wang , W. Sun , C. Guo , W. Wu , J. Wang , ACS Nano 2021, 15, 10175. 34101427 10.1021/acsnano.1c02094

[36] W. Zhang , Y. Li , S. Peng , J. Mater. Chem. A 2017, 5, 13072.

[37] E. V. Golubina , E. S. Lokteva , A. V. Erokhin , A. A. Veligzhanin , Y. V. Zubavichus , V. A. Likholobov , V. V. Lunin , J. Catal. 2016, 344, 90.

[38] H. Guo , H.‐Y. Niu , C. Liang , C.‐G. Niu , Y. Liu , N. Tang , Y. Yang , H.‐Y. Liu , Y.‐Y. Yang , W.‐J. Wang , Chem. Eng. J. 2020, 401, 126072.

[39] G. X. Pei , X. Y. Liu , X. Yang , L. Zhang , A. Wang , L. Li , H. Wang , X. Wang , T. Zhang , ACS Catal. 2017, 7, 1491.

[40] R. Prins , Chem. Rev. 2012, 112, 2714. 22324402 10.1021/cr200346z

[41] X. Zhang , G. Cui , H. Feng , L. Chen , H. Wang , B. Wang , X. Zhang , L. Zheng , S. Hong , M. Wei , Nat. Commun. 2019, 10, 5812. 31862887 10.1038/s41467-019-13685-2 PMC6925196

[42] R. Tu , K. Liang , Y. Sun , Y. Wu , W. Lv , C. Q. Jia , E. Jiang , Y. Wu , X. Fan , B. Zhang , Q. Lu , B. Zhang , X. Xu , Chem. Eng. J. 2023, 452, 139526.

[43] R. Gao , L. Pan , H. Wang , Y. Yao , X. Zhang , L. Wang , J.‐J. Zou , Adv. Sci. 2019, 6, 1900054. 10.1002/advs.201900054 PMC6523378 31131202

[44] T. Zheng , C. Liu , C. Guo , M. Zhang , X. Li , Q. Jiang , W. Xue , H. Li , A. Li , C.‐W. Pao , J. Xiao , C. Xia , J. Zeng , Nat. Nanotechnol. 2021, 16, 1386. 34531557 10.1038/s41565-021-00974-5

[45] J. Shan , J. Liu , M. Li , S. Lustig , S. Lee , M. Flytzani‐Stephanopoulos , Appl. Catal. B 2018, 226, 534.

[46] R. Reske , H. Mistry , F. Behafarid , B. R. Cuenya , P. Strasser , J. Am. Chem. Soc. 2014, 136, 6978. 24746172 10.1021/ja500328k

[47] Q. He , D. Liu , J. H. Lee , Y. Liu , Z. Xie , S. Hwang , S. Kattel , L. Song , J. G. Chen , Angew. Chem., Int. Ed. 2020, 59, 3033. 10.1002/anie.201912719 31826317

[48] H. Yuan , M. Hong , F. Dong , Y. Chen , X. Du , X. Huang , J. Gao , S. Yang , Appl. Catal. B 2023, 334, 122864.

[49] M. Wen , S. Song , Q. Liu , H. Yin , K. Mori , Y. Kuwahara , G. Li , T. An , H. Yamashit , Appl. Catal. B 2021, 282, 119511.

[50] S. Li , L. Wang , M. Wu , Y. Sun , X. Zhu , Y. Wan , Chin. J. Catal. 2020, 41, 1337.

[51] M. Boronat , P. Concepción , A. Corma , S. González , F. Illas , P. Serna , J. Am. Chem. Soc. 2007, 129, 16230. 18052067 10.1021/ja076721g

[52] Z. Wang , H. Wang , Y. Shi , C. Liu , L. Wu , S. Liang , Chem. Eng. Sci. 2022, 41, 117936.

[53] S. L. Scott , ACS Catal. 2018, 8, 8597.

[54] S. Zhang , H. Li , L. Wang , J. Liu , G. Liang , K. Davey , J. Ran , S.‐Z. Qiao , J. Am. Chem. Soc. 2023, 262, 6410. 10.1021/jacs.2c13590 36913199

[55] M. Li , Z. Zhao , Z. Xia , M. Luo , Q. Zhang , Y. Qin , L. Tao , K. Yin , Y. Chao , L. Gu , W. Yang , Y. Yu , G. Lu , S. Guo , Angew. Chem., Int. Ed. 2021, 133, 8324. 10.1002/anie.202016199 33434387

[56] A. Vojvodic , J. K. Nørskov , Science 2011, 334, 1355. 22158809 10.1126/science.1215081

[57] S. Chen , S. Wang , P. Hao , M. Li , Y. Zhang , J. Guo , W. Ding , M. Liu , J. Wang , X. Guo , Appl. Catal. B 2021, 304, 120996.

[58] Y. Lv , M. Han , W. Gong , D. Wang , C. Chen , G. Wang , H. Zhang , H. Zhao , Angew. Chem., Int. Ed. 2020, 59, 23521. 10.1002/anie.202009913 32909312

[59] J. Song , Z.‐F. Huang , L. Pan , K. Li , X. Zhang , L. Wang , J.‐J. Zou , Appl. Catal. B 2018, 227, 386.

[60] P. Ferrin , S. Kandoi , A. U. Nilekar , M. Mavrikakis , Surf. Sci. 2012, 606, 679.

[61] W. Ye , J. Yu , Y. Zhou , D. Gao , D. Wang , C. Wang , D. Xue , Appl. Catal. B 2016, 181, 371.

[62] J. Xia , G. He , L. Zhang , X. Sun , X. Wang , Appl. Catal. B 2016, 180, 408.

[63] X.‐Q. Zhang , R.‐F. Shen , X.‐J. Guo , X. Yan , Y. Chen , J.‐T. Hu , W.‐Z. Lang , Chem. Eng. J. 2021, 408, 128018.

[64] C. Lu , M. Wang , Z. Feng , Y. Qi , F. Feng , L. Ma , Q. Zhang , X. Li , Catal. Sci. Technol. 2017, 7, 1581.

[65] S. Iihama , S. Furukawa , T. Komatsu , ACS Catal. 2016, 6, 742.

[66] L. Jiang , K. Liu , S.‐F. Hung , L. Zhou , R. Qin , Q. Zhang , P. Liu , L. Gu , H. M. Chen , G. Fu , N. Zheng , Nat. Nanotechnol. 2020, 15, 848. 32747741 10.1038/s41565-020-0746-x

[67] X. Huang , X. Hou , J. Zhao , L. Zhang , Appl. Catal. B 2016, 181, 127.

[68] M. Huš , B. Likozar , M. Grilc , Chem. Eng. J. 2022, 436, 135070.

