# Supplementary material for: Graphene Chainmail Shelled Dilute Ni─Cu Alloy for Selective and Robust Aqueous Phase Catalytic Hydrogenation
Source: Adv Sci (Weinh). 2024 Jan 19;11(13):2304349. doi: 10.1002/advs.202304349 (PMC10987116; doi:10.1002/advs.202304349)
Supplement: Supplementary file 1 — Supporting Information [file ADVS-11-2304349-s002.pdf]

ADVANCED SCIENCE
Open Access

Supporting Information

for *Adv. Sci.*, DOI 10.1002/advs.202304349

Graphene Chainmail Shelled Dilute Ni—Cu Alloy for Selective and Robust Aqueous Phase Catalytic Hydrogenation

Haifeng Yuan, Mei Hong, Xianzhen Huang, Weitao Qiu, Feng Dong, Yu Zhou, Yanpeng Chen, Jinqiang Gao* and Shihe Yang**

Supporting Information

Graphene Chainmail Shelled Dilute Ni-Cu Alloy for Selective and Robust Aqueous Phase Catalytic Hydrogenation

Haifeng Yuan, Mei Hong, Xianzhen Huang, Weitao Qiu, Feng Dong, Yu Zhou, Yanpeng Chen, Jinqiang Gao,* Shihe Yang**

H. Yuan, X. Huang, Dr. W. Qiu, F. Dong, Y. Zhou, Dr. J. Gao, Dr. M. Hong, Prof. S. Yang
Guangdong Provincial Key Lab of Nano-Micro Materials Research, School of Chemical Biology and Biotechnology, Shenzhen Graduate School, Peking University Shenzhen, Guangdong 518055, China
E-mail: hongmei@pku.edu.cn, jinqiang_gao@pku.edu.cn, chsyang@pku.edu.cn

Y. Chen
Shenzhen Key Laboratory of Organic Pollution Prevention and Control, Environmental Science and Engineering Research Center, Harbin Institute of Technology (Shenzhen), Shenzhen, Guangdong 518055, China

Prof. S. Yang
Insitute of Biomedical Engineering, Shenzhen Bay Laboratory, Shenzhen, Guangdong 518055, China

Contents list of the supporting information

1. **Experimental**......1

Chemicals and materials 1

Preparation of catalysts 1

The catalytic reactions......1

Characterization of catalysts 3

Density functional theory (DFT) calculation methods......6

2. **Figures and tables** 7

Figure S1. Phase diagram of Cu-Ni alloy system, data from *SGnobl noble metal alloy database*. 7

Figure S2. a, b) The FE-SEM images of $CuNi_{0.05}$@OC sample. c) The corresponding 3D illustration. 7

Figure S3. a) XRD patterns of various catalysts. b) In-depth analysis of the (111) crystalline surface of the samples......8

Figure S4. A positive correlation between Ni content and the number of graphene layers in the samples. 8

Figure S5. a-d) TEM image of Cu/OC, $CuNi_{0.01}$@OC, $CuNi_{0.25}$@OC, and Ni@OC, respectively, insets are the NP size distributions. e-i) The HAADF-STEM image of Cu/OC with elemental mappings, and the corresponding elemental line-scanning profiles of the representative NPs. j-n) The HAADF-STEM image of Ni@OC with elemental mappings, and the corresponding elemental line-scanning profiles of the representative NPs. o-s) The HAADF-STEM image of $CuNi_{0.25}$@OC with elemental mappings, inset is the corresponding elemental line-scanning profiles of the representative NPs......9

Figure S6. a) The N_2 adsorption-desorption isotherms and b) the BJH pore size distribution curves of the samples......10

Figure S7. XPS survey spectra of various catalysts. 11

Figure S8. HR XPS spectra of $CuNi_{0.01}$@OC sample. 11

Figure S9. H_2-TPR profile of $CuNi_{0.65}$@OC sample. 12

Figure S10. First derivatives of the a) Cu and b) Ni *K*-edge XANES region. 12

Figure S11. The valence state of various species obtained from a) Cu and b) Ni *K*-edge XANES spectra.13

Figure S12. Cu L_3VV Auger spectra of $CuNi_{0.05}$@OC and Cu/OC sample. 13

Figure S13. The EXAFS wavelet transform spectra of a) $CuNi_{0.05}$@OC, b) Cu foil, c) Cu_2O, and d) CuO, respectively. 14

Figure S14. Cu *K*-edge EXAFS fitting curves of Cu foil and $CuNi_{0.05}$@OC: a, b) The Fourier transformation of filtered $\chi(k)\cdot k^3$; c, d) The radial structure functions obtained by Fourier transformation of k^3-weighted EXAFS results....................15

Figure S15. In-situ CO-DRIFTS spectra fitting of $CuNi_{0.05}$@OC and Cu/OC sample flushed with Ar for 5 min at room temperature. Cu: blue; Ni: green; C: gray; O: yellow. 16

Figure S16. Ni *K*-edge EXAFS fitting curves of Ni foil and $CuNi_{0.05}$@OC: a, b) The Fourier transformation of filtered $\chi(k)\cdot k^3$; c, d) The radial structure functions obtained by Fourier transformation of k^3-weighted EXAFS results....................17

Figure S17. Turnover frequency based on Ni or Cu atoms for the various catalysts. 18

Figure S18. First-order kinetic fit for *p*-CNB hydrogenation to *p*-CAN at different temperatures. Reaction conditions: hydrazine hydrate (1.5 mmol); *p*-CNB (0.5 mmol); catalyst (20 mg); water (15 mL); 1000 rpm. 18

Figure S19. Hot filtration test for *p*-CNB hydrogenation. Reaction conditions: hydrazine hydrate (1.5 mmol); *p*-CNB (0.5 mmol); $CuNi_{0.05}$@OC catalyst (20 mg); water (15 mL); T =50 °C; 1000 rpm. Yields were determined by GC-MS. 19

Figure S20. Magnetization curves at room temperature, inset in the upper left corner is magnified magnetic hysteresis loop from -900 to 900 Oe for $CuNi_{0.05}$@OC, in the lower right corner is magnified magnetic hysteresis loop from -20k to 20k Oe for Cu/OC, and in the lower left corner is photos of the $CuNi_{0.05}$@OC sample from dispersed in ultra-pure water to aggregation under external magnetic field. 19

Figure S21. Photos of CTH of *p*-CNB using $CuNi_{0.05}$@OC at different stages of hydrogenation and recovery. 20

Figure S22. Reusability of Cu/OC in the consecutive cycles. Reaction conditions: hydrazine hydrate (1.5 mmol); *p*-CNB (0.5 mmol); catalyst (20 mg); water (15 mL); T =50 °C; 4 h; 1000 rpm. 20

Figure S23. The XRD patterns of used catalysts....................21

Figure S24. a) The N_2 adsorption-desorption isotherms and b) the BJH pore size distribution curves of used catalysts. 21

Figure S25. The HR-TEM images of used catalysts. 22

Figure S26. HR-XPS spectra of used $CuNi_{0.05}$@OC and Cu/OC catalysts in comparison with those of the fresh ones. 23

Figure S27. Stability tests of $CuNi_{0.05}$@OC in CTH of *p*-CNB to *p*-CAN after air exposure. Reaction conditions: hydrazine hydrate (1.5 mmol); *p*-CNB (0.5 mmol); catalyst (20 mg); water (15 mL); T =50 ℃; 1000 rpm. Reaction time: 4 h (red bars), 1 h (red stars). 23

Figure S28. Effect of Ni/Cu molar ratio on the catalytic performance and physicochemical property of the series catalysts including Cu/OC, $CuNi_{0.01}$@OC, $CuNi_{0.05}$@OC, $CuNi_{0.25}$@OC, $CuNi_{0.65}$@OC, and Ni@OC: a) *p*-CNB yield; b) ratio of C-M species; c) ratio of oxygen vacancy; d) I_D/I_G in the carbon coating; e) surface area; f) pore volume. 24

Figure S29. Geometric configurations of Ni (111) surfaces. Ni: green; C: gray; O: yellow.........25

Figure S30. Calculated work function (Φ) of Ni (111) surfaces. 25

Figure S31. a) AFM image of $CuNi_{0.05}$@OC, b) the corresponding 3D image, and c) height profile of the yellow and cyan lines in panel (a). d) Surface potential distribution of $CuNi_{0.05}$@OC under dark irradiation, e) the corresponding 3D image, and f) potential profile of the greed and red lines in panel (d)........26

Figure S32. a) Charge density differential analysis of $CuNi_{0.05}$@OC presented in half-section for a clearer representation of electron migration. The yellow and blue contours indicate electron increment and depletion, respectively. Cu: blue; Ni: green; C: gray; O: yellow; same below. b) Bader charge analysis of $CuNi_{0.05}$@OC. The numbers stand for the amount of charge carried by corresponding atoms of pristine $CuNi_{0.05}$@OC system, which reveal that the outer oxygen-doped carbon (OC) shell carries negative charges from the internal Cu-Ni dilute alloy. c) Bader charge analysis of Cu-Ni dilute alloy. The numbers stand for the amount of charge carried by corresponding atoms of pristine CuNi(111) surfaces, which reveal that isolated Ni atom carries negative charges from sub-surface Cu atoms...27

Figure S33. a) The Mott-Schottky type contact of $CuNi_x$@OC catalysts. b) The Mott-Schottky contacting interface of diluted alloy $CuNi_{0.05}$ NPs and OC shell of $CuNi_{0.05}$@OC samples. Cu: blue; Ni: green; OC: grey. 28

Figure S34. Relationship between d-band center and ΔG_{H*} values over various catalyst models.28

Figure S35. Differential charge density diagrams of H-adsorption state for a) $CuNi_{0.05}$@OC and b) Cu/OC models. 29

Figure S36. Top and side views of the optimized structure of *p*-CNB molecule on a) Ni@OC and b) $CuNi_{0.05}$@OC. The letter d represents the adsorption distance between *p*-CNB molecule and substrate. 30

Figure S37. a) Structural diagram of *p*-CNB. Molecular structure of gaseous *p*-CNB, with the labeled bond length (L(N-O_1)) and bond angle (∠(N-O_1)) of N-O_1. Correspondingly, the bond lengths and bond angles of N-O_1 of *p*-CNB after adsorption on the Ni@OC b) and CuNi@OC c). C: orange; O: yellow; N: blue; H: white. 30

Figure S38. The positive correlation relationship between TOF value and a) τ, and b) the content of $O_β$. 31

Figure S39. Photographs of reaction mixture containing a) 1 g WO_3 and b) mixture of 1 g WO_3 and 20 mg $CuNi_{0.05}$@OC before and after hydrogenation. Reaction conditions: hydrazine hydrate (1.5 mmol); *p*-CNB (0.5 mmol); water (15 mL); T =50 °C; 4 h; 1000 rpm. 31

Figure S40. a) UV-vis absorption spectra of *p*-NP before and after addition of excessive $NaBH_4$, and UV-vis intensity of *p*-NP aqueous solution in the presence of only $NaBH_4$ and the carbon carrier recorded at different intervals. b) Plot of ln (C_0/C_t) vs. time. c) Comparison of the CTH conversion of *p*-NP to *p*-AP over different catalysts (see Table S10 for details). Reaction conditions: $NaBH_4$ (0.2 M); *p*-NP (10 mM); catalyst (2 mg); T =25 °C; 1000 rpm. 32

Figure S41. Schematic illustration of FF hydrogenation pathways, our work in selective carbonyl hydrogenation of FF toward FAOL, and applications of hydrogenated products in different engineering aspects. 33

Table S1. The ICP-AES of the different samples. 34

Table S2. Textural properties of the samples. 34

Table S3. XPS characterization data of the different catalysts. 35

Table S4. EXAFS fitting parameters at the Cu or Ni *K*-edge for various samples. 36

Table S5. Cu L_3VV Auger spectra fitting parameters for Cu/OC and $CuNi_{0.05}$@OC samples. 37

Table S6. Summary of CTH performance of *p*-CNB with hydrazine hydrate as hydrogen source over various catalysts. 37

Table S7. Thermodynamic parameter values for the catalytic reduction of *p*-CNB using Cu/OC, Ni@OC, $CuNi_{0.05}$@OC, and $CuNi_{0.25}$@OC catalysts calculated using Arrhenius formula and Elling equation. 38

Table S8. Recycling of Cu/OC and $CuNi_{0.05}$@OC catalysts in the catalytic reduction of *p*-CNB base on the TOF values [a]. 39

Table S9. CTH of a rich variety of functionalized nitroaromatics over $CuNi_{0.05}$@OC [a]. 40

Table S10. Summary of CTH performance of *p*-NP with sodium borohydride as hydrogen source over various catalysts. 41

Table S11. Selective hydrogenation of various biomass-derived aldehydes over the $CuNi_{0.05}$@OC [a]. 42

Table S12. Summary of SH performance of FF with hydrogen as hydrogen source over various catalysts. 43

Table S13. Model structure information of CuNi@OC. 44

Table S14. Model structure information of Ni@OC. 49

Table S15. Model structure information of Cu/OC. 54

3. **References** 56

1. Experimental

Chemicals and materials

Copper nitrate hexahydrate [$Cu(NO_3)_2{\cdot}6H_2O$, 99.9%], nickel nitrate hexahydrate [$Ni(NO_3)_2{\cdot}6H_2O$, 99.9%], and hydrazine hydrate solution ($N_2H_4{\cdot}H_2O$, 80 wt.%) were purchased from Shanghai Aladdin Biochemical Technology Co., Ltd. Terephthalic acid, *p*-chloronitrobenzene ($C_6H_4ClNO_2$, 99.8%), *p*-nitrophenol ($C_6H_5NO_3$, 99.8%), and furfural ($C_5H_4O_2$, 98%) were all purchased from Shanghai Macklin Biochemical Co., Ltd. Other chemicals were purchased from Sinopharm Chemical Reagents Co., Ltd or Shanghai Bide Pharmatech Co., Ltd, and used without further purification. Deionized (DI) water was used during the experiment.

Preparation of catalysts

$CuNi_x$@OC composites (where x represents the molar ratio of Ni/Cu in the precursor) were obtained by direct pyrolysis of Cu/Ni-MOF coordinated by terephthalic acid. Specifically, a certain amount of $Ni(NO_3)_2{\cdot}6H_2O$ was dissolved in 60 mL of methanol and 100 mL of ultra-pure water, and the mixed solution formed by 50 mmol of terephthalic acid and 23 mmol of NaOH was added dropwise under stirring until the PH ≈ 10.83, which is called solution A. 12 mmol of $Cu(NO_3)_2{\cdot}6H_2O$ was dissolved in 60 mL of methanol and 100 mL of ultrapure water to form solution B. Then, under nitrogen bubbling and continuous stirring, solution B was slowly added to solution A. After keeping the reaction at room temperature for 6.0 h, the precipitate was obtained by centrifugation, washed with water and methanol for three times, and then completely dried overnight in a vacuum furnace to obtain Cu/Ni-MOF. A series of Cu:Ni ratios (1:0, 1:0.01, 1:0.05, 1:0.25, 1:0.65, 0:1) were designed by changing the amount of transition metal nitrate. Then, the obtained Cu/Ni-MOF was pyrolyzed in a tube furnace at 550 °C under argon for 2.0 h to produce a self-supporting $CuNi_x$@OC catalyst.

The catalytic reactions

For the catalytic transfer hydrogenation of nitroaromatics, nitroaromatic hydrocarbon (0.5 mmol), $CuNi_x$@OC catalyst (20 mg) and H_2O (15 mL) were added to a round-bottom flask and mixed, followed by $N_2H_4{\cdot}H_2O$ (1.5 mmol). The flask was sealed with a balloon and reacted at 1000 rpm. Then, the reaction

liquid was extracted with ethyl acetate, and the supernatant was extracted and analyzed by gas chromatography-mass spectrometry (GC-MS, Agilent 5977E/B). For catalyzing the liquid phase hydrogenation of *p*-nitrophenol (*p*-NP), $NaBH_4$ was used as hydrogen source and the whole reduction process was monitor by UV-2550 spectroscopy. Specifically, 20 mL *p*-NP solution (10 mM), catalyst (2 mg), and $NaBH_4$ (0.2 M) were added into a 50 mL round flask placed in a water bath at a constant temperature of 25 °C. Beer-Lambent law corrects the concentration of *p*-NP and *p*-aminophenol (*p*-AP) by absorbance at 317 nm and 295 nm, respectively. The liquid phase hydrogenation of furfural (FF) was carried out in a 100 mL stainless steel autoclave. Catalyst (20 mg), FF (0.75 mmol), and H_2O (15 mL) as solvent were added to a reactor, which was sealed, ventilated, and exchanged with H_2 three times to exhaust air. After hydrogenation at preset temperature, pressure, and time, the reaction supernatant was collected by centrifugation, and aliquots were taken for GC-MS analysis to monitor the conversion of FF and the selectivity to furfuryl alcohol (FAOL).

For the recyclability test, the used $CuNi_x$@OC catalyst was recovered using an external magnet, then washed thoroughly with ethyl acetate, and finally dried in a vacuum oven at room temperature for the next catalytic test. The initial molar concentration of substrate was recorded as C_0, while C_t refers to the molar concentration of substrate at time *t*. To show the intrinsic activity of catalysts, the conversion of substrate was restricted to less than 20%, and the turnover frequency (TOF) was denoted as the molar amount of substrate converted per mole of metal sites per hour. The following equations were used to calculate substrate conversion, product selectivity, yield, reaction rate, activation energy (E_a), activation entropy (ΔS^{0*}), activation enthalpy (ΔH), and Gibbs free-energy of activation (ΔG), respectively. Arrhenius plots gave the E_a, while the ΔS^{0*} and ΔH were calculated based on the transition state theory and the Eyring equation.

Equations for conversion, selectivity, yield, and reaction rate:

$$\text{Conversion}_{\text{substrate}}(\%) = M_{\text{substrate converted}}/M_{\text{substrate in feed}} \times 100 \quad (1)$$

$$\text{Selectivity}_{\text{product}}(\%) = M_{\text{substrate formed}}/M_{\text{substrate converted}} \times 100 \quad (2)$$

$$\text{Yield}_{\text{product}}(\%) = [\text{Conversion}_{\text{substrate}}(\%) \times \text{Selectivity}_{\text{product}}(\%)]/100 \quad (3)$$

$$\text{Reaction rate} = -[dC_t/dt] \quad (4)$$

where C_t refers to the molar concentration of substrate at time t.

Equations for E_a:

$$\ln K = \ln A - [E_a/(R \times T)] \quad (5)$$

where K is reaction rate constant at temperature T, A is the arrhenius constant, and R is the molar gas constant.

Equations for ΔH and ΔS^{0*}:

$$\ln(K/T) = \ln(k_B/h) - [\Delta H/(R \times T)] + (\Delta S^{0*}/R) \quad (6)$$

where h, k_B, and R are the Planck constant, Boltzmann constant, and molar gas constant, respectively.

Equations for ΔG:

$$\Delta G = \Delta H - (T \times \Delta S^{0*}) \quad (7)$$

where T is absolute temperature in Kelvin.

Characterization of catalysts

The prepared samples were characterized by an array of analytical methods. The actual contents of Cu and Ni elements were determined by inductively coupled plasma emission spectrometry (ICP-AES). The nitrogen adsorption-desorption analysis was carried out at -196 °C by using a Micromeritics ASAP 2010 analyzer. Prior to each measurement, the sample was pretreated at 300 °C for 3 h under vacuum. Based on Brunauer-Emmett-Teller (BET) and Barrett-Joyner-Halenda (BJH) methods, the surface areas and pore size distributions were obtained. X-ray powder diffraction pattern (XRD) was collected on a Rigaku XRD-6000 diffractometer, which is equipped with the in-situ reaction cell connected to a gas mass flow and temperature programming device. The temperature was ramped at 10 °C min^{-1} between 25 and 800 °C, and was held at the corresponding temperature point for 5 min before XRD sweep, the flow rate of nitrogen was 10 mL min^{-1}. The phases of components were identified based on JCPDS standard cards. The electron microscope images were taken by JSM-6701 field emission scanning electron microscope (FE-SEM) and FEI TECNAI G2 F30 high-resolution transmission electron microscope (HR-TEM). The compressive strain (τ) is defined as percent deviation of metal lattice constant from its theoretical value.

The geometric phase analysis (GPA) technique was employed to analyze the displacement information from interference fringes by reference to lattice regions, which is divided into the following steps: (1) Fast Fourier Transform (FFT) on the obtained HR-TEM images; (2) Selection of diffraction spots in different lattice directions; (3) Converting FFT into inverse FFT; (4) Calculating the geometric phase image by using the relationship between the phase of the local Fourier component $P_g(r)$ and the displacement field component u(r). g is the reciprocal lattice vector. $P_g(r) = -2\pi g$ Âu(r); (5) Calculating phase images $P_{g1}(r)$ and $P_{g2}(r)$ of two displacement fields; (6) The displacement field differentiation to obtain the strain field. The Raman spectra were obtained by using high-resolution Raman spectrometer (LabRAM HR Evolution, HORIBA). The diffuse reflectance UV-vis (UV-vis-DR) spectra were collected with a Shimadzu UV-3600 spectrophotometer. X-ray photoelectron spectroscopy (XPS) was obtained on ESCALAB 250xi spectrometer with a working voltage of 12 kV, a current of 12 mA, and monochromatic Al Kα radiation (hν =1486.6 eV) to check the electronic characteristics of the sample surface. The spectrum was calibrated by using the binding energy of C 1s peak at 284.8 eV and analyzed with the CasaXPS software. For the charge compensation dual beam sources, the background pressure was set to $<2\times10^{-7}$ mbar Ar during measurement. The hydrogen temperature programmed reduction (H_2-TPR) and hydrogen temperature programmed desorption (H_2-TPD) were obtained on a Micromeritics Chemisorb 2750 chemical adsorption instrument with a thermal conductivity detector (TCD). For the H_2-TPR test, 100 mg samples were weighed and placed in a quartz tube and pretreated under He atmosphere at 150 °C for 90 min. Then, it was cooled to 50 °C in 10% H_2/Ar mixed gas and kept at this temperature for 60 min. Finally, in the atmosphere of 10% H_2/Ar mixed gas, the temperature was raised from 50 °C to 800 °C at a rate of 10 °C min^{-1}, and the hydrogen consumption was detected by TCD. In the H_2-TPD test, 100 mg samples were weighed and placed in a quartz tube, which was first purged with He atmosphere at 150 °C for 90 min. Then, it was cooled to 50 °C in 10% H_2/Ar mixed gas and kept at this temperature for 90 min. Prior to heating up, the samples were held for 60 min under Ar atmosphere. Finally, in Ar atmosphere, the temperature was raised from 50 °C to 800 °C at a rate of 10 °C min^{-1}. The hydrogen desorption amount of the sample was detected by TCD. Diffuse reflection Fourier transform infrared spectroscopy (DRIFTS) was collected on Brook tensor instrument equipped with high sensitivity MCT detector. Prior to the test, the in-situ cell was heated to 150 °C and held for 60 min under air atmosphere to remove volatile substances. About 30 mg of the sample was pressed into the cell, and all spectra were recorded in the

range of 4000-1200 cm^{-1} with a resolution of 4 cm^{-1} and scanned 64 times. In a typical experiment, the sample was first purged with H_2/Ar at 150 °C for 60 min, then cooled to room temperature and the background was recorded. Then, the sample was exposed to carbon monoxide or the mixture vapor of substrate (equimolar chlorobenzene and nitrobenzene) for 30 min. Finally, at room temperature, the chemical desorption of the sample was scanned at different times. Magnetic property of the catalyst was tested by an ADE-EV7 vibrating sample magnetometer (VSM) system, and the saturation magnetization was recorded at 2T. Atomic force microscopy (AFM) images were collected from Bruker Multimode 8 system and ZEISS Merlin system, and Kelvin probe force microscope (KPFM) images are collected using a Dimension Edge. The extended X-ray absorption fine structure (EXAFS) of Cu or Ni were measured at 21A X-ray nanodiffraction (XND) beamline of Taiwan Photon Source (TPS), National Synchrotron Radiation Research Center (NSRRC). This beamline adopted 4-bounce channel-cut Si (111) monochromator for mono-beam X-ray nanodiffraction and X-ray absorption spectroscopy. The end-station was equipped with three ionization chambers and Lytle/SDD detector after the focusing position of KB mirror for transmission and fluorescence mode X-ray absorption spectroscopy (XAS). The photon flux on the sample ranges from 1×10^{11}~3×10^{9} photon/sec for X-ray energy from 6-27 keV. Data analysis and EXAFS fitting were performed with the Athena and Artemis programs of the Demeter data analysis packages that utilizes the FEFF6 program.[1,2] The energy calibration of the sample was conducted through a standard Cu or Ni foil, which as a reference was simultaneously measured. EXAFS of the Cu or Ni foil is fitted and the obtained amplitude reduction factor S_0^2 value of 0.81 or 0.85 was set to determine the coordination numbers (*CNs*). A linear function was subtracted from the pre-edge region, then the edge jump was normalized. The χ(k) data were isolated by subtracting a smooth, third-order polynomial approximating the absorption background of an isolated atom. The k^3-weighted $\chi(k)$ data were Fourier transformed after applying a Hanning window function (Δk = 1.0). For the global amplitude EXAFS, (*CN*, *R*, σ^2, and ΔE_0) were obtained by nonlinear fitting, with least-squares refinement in *R*-space.

Density functional theory (DFT) calculation methods

We performed the first-principles calculations in the frame of density functional theory (DFT) with the Vienna ab initio simulation package (VASP).[3,4] The exchange-correlation energy is described by the Perdew-Burke-Ernzerhof (PBE) form of generalized-gradient approximation (GGA) exchange-correlation energy functional.[5,6] The DFT-D3 semiempirical correction was described via Grimme's

scheme method and the cutoff energy for the plane-wave basis set was set to 450 eV.[3,6] The projector augmented wave (PAW) method was used to describe the electron interactions.[4] The structure optimizations of CuNi@OC, Ni@OC, and Cu/OC have been carried out by allowing all atomic positions to vary and fixing lattice parameters until the energy difference of successive atom configurations was less than 1 × 10^{-4} eV. The maximum force on each atom in the relaxed structures was less than 0.03 eV $Å^{-1}$ as the convergence criteria of structure optimization. The k-point spacing was set to be smaller than 0.03 $Å^{-1}$ over Brillouin zone (BZ) at structure optimization.[7] During the further calculations, the 0.31%, 0.13% and 0.04% lattice strains were applied for $CuNi_{0.05}$@OC, Ni@OC, and Cu/OC to simulate various degrees of compressive strain, respectively. the in-plane lattice constants of samples were decreased and then fixed, after which the atomic positions were stressed to simulate the compressive strain.

To calculate the adsorption energies of H atom, *p*-CNB molecule, or O_2 molecule on Cu/OC, Ni@OC, or $CuNi_{0.05}$@OC, we first construct the corresponding surfaces. In order to obtain the most stable structures, their structure optimizations were performed by setting the thickness of 15 Å in vacuo. The adsorption energies were calculated by the following equation:

$$\Delta E = E_{system} - E_{slab} - E_{adsorbate}$$

where, E_{system}, E_{slab} and $E_{adsorbate}$ represent the total energies of the adsorption system, substrate surface and adsorbate, respectively.

Note that the $CuNi_{0.05}$@OC model illustration contains a total of 322 atoms, and is based on four Ni atom doping into the Cu NPs, and five O atom doping into the C_{240}. Detailed model structure information can be found in **Table S12-S14**.

2. Figures and tables

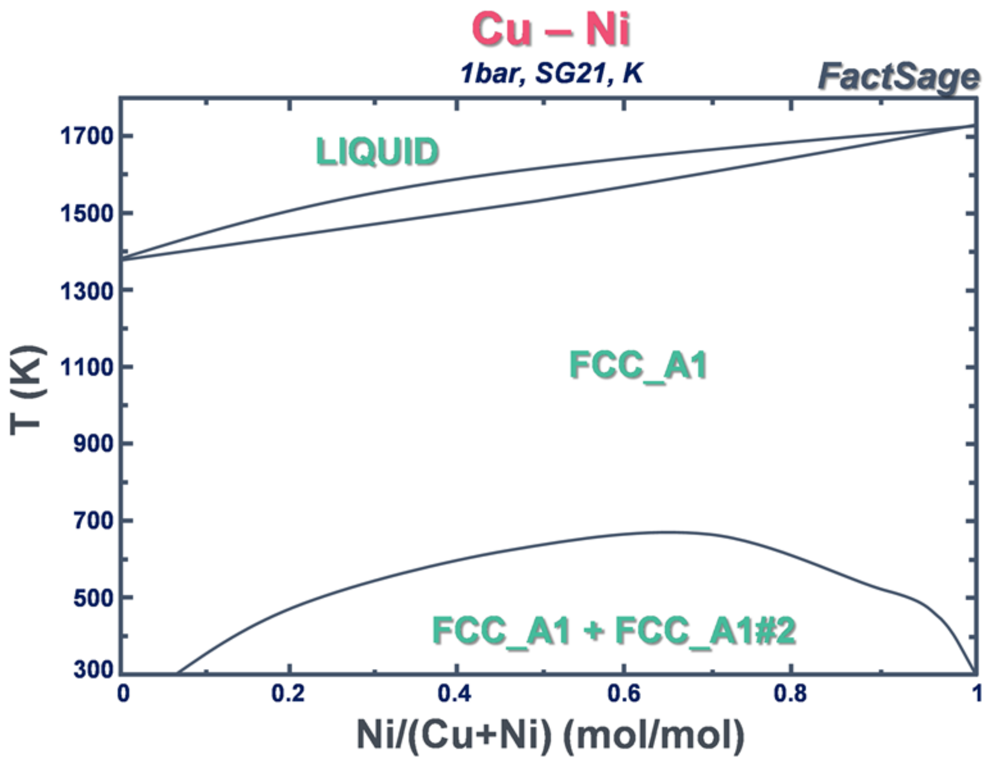

Figure S1. Phase diagram of Cu-Ni alloy system, data from *SGnobl noble metal alloy database*.

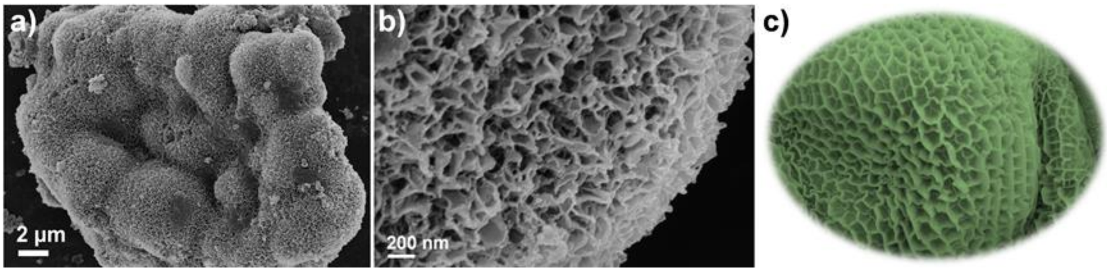

Figure S2. a, b) The FE-SEM images of $CuNi_{0.05}$@OC sample. **c)** The corresponding 3D illustration.

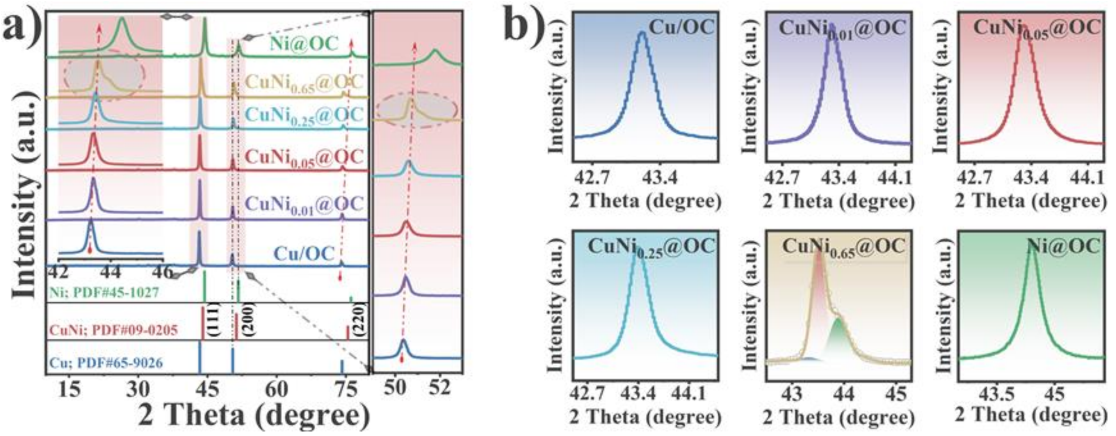

Figure S3. a) XRD patterns of various catalysts. **b)** In-depth analysis of the (111) crystalline surface of the samples.

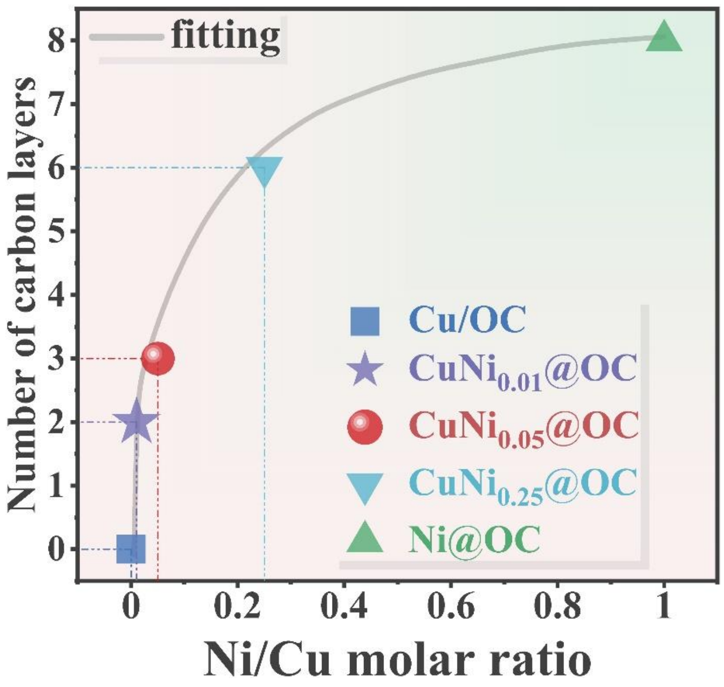

Figure S4. A positive correlation between Ni content and the number of graphene layers in the samples.

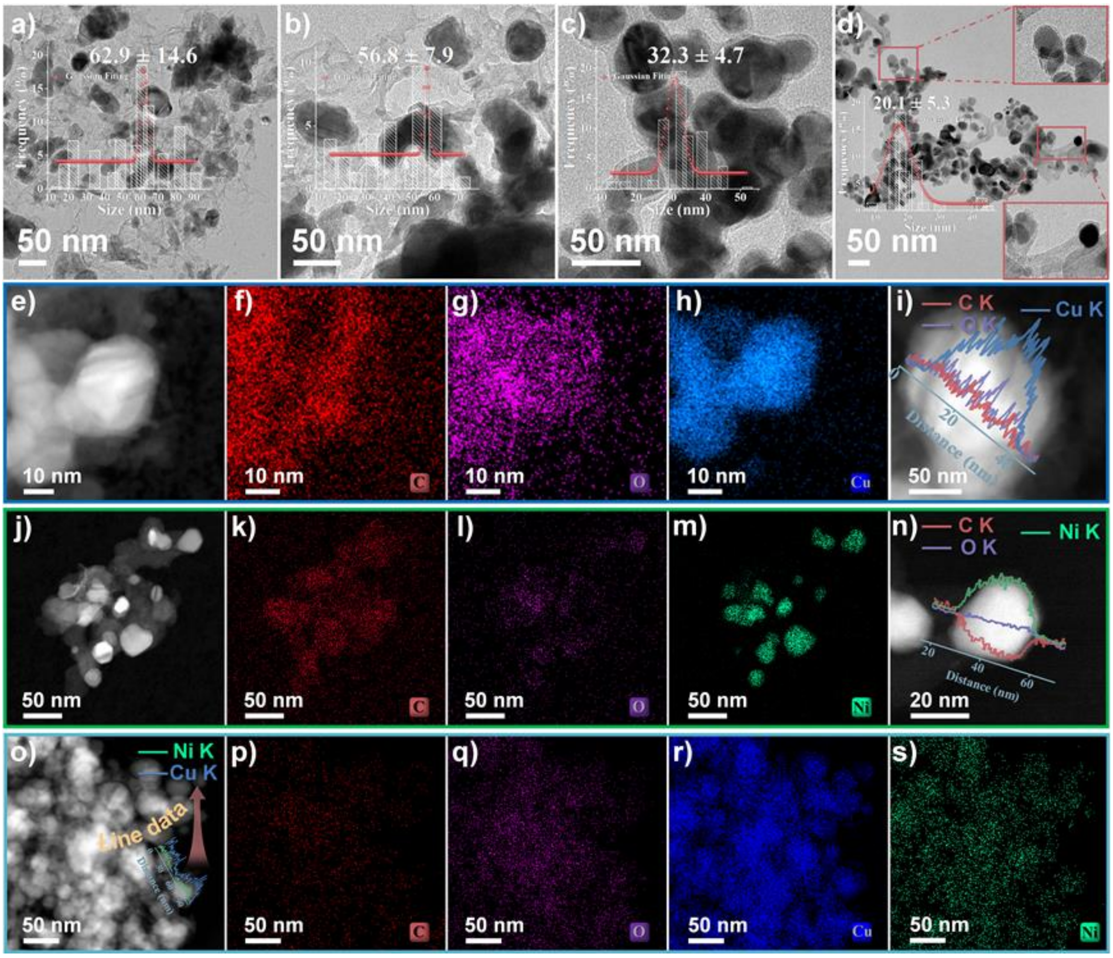

Figure S5. a-d) TEM image of Cu/OC, $CuNi_{0.01}$@OC, $CuNi_{0.25}$@OC, and Ni@OC, respectively, insets are the NP size distributions. **e-i)** The HAADF-STEM image of Cu/OC with elemental mappings, and the corresponding elemental line-scanning profiles of the representative NPs. **j-n)** The HAADF-STEM image of Ni@OC with elemental mappings, and the corresponding elemental line-scanning profiles of the representative NPs. **o-s)** The HAADF-STEM image of $CuNi_{0.25}$@OC with elemental mappings, inset is the corresponding elemental line-scanning profiles of the representative NPs.

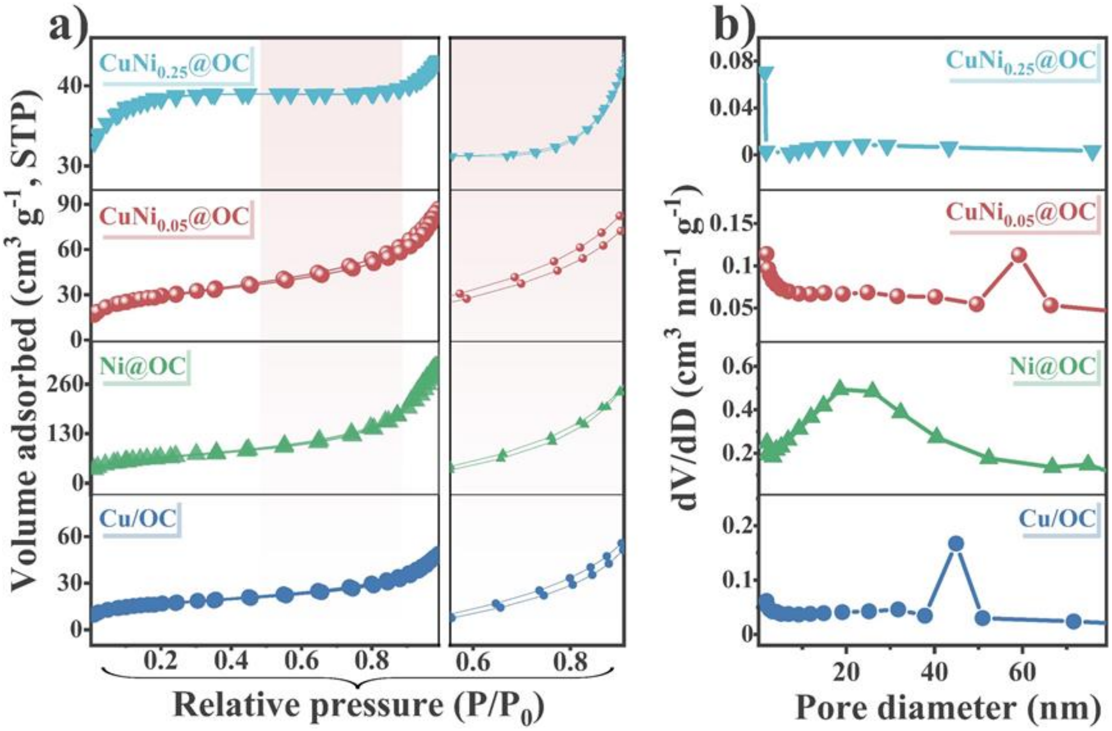

Figure S6. a) The N_2 adsorption-desorption isotherms and **b)** the BJH pore size distribution curves of the samples.

The N_2 adsorption-desorption isotherms of the catalysts were used to study the effect of Ni doping on texture characteristics. As shown in ***Figure S6****, all samples show typical type IV isotherms in the relative pressure range of P/P_0 = 0.6-1.0, which confirmed the desired porous property.*[8] *$CuNi_{0.05}$@OC shows a high surface area of 99.8 $m^2\ g^{-1}$, which is between Ni@OC (232.1 $m^2\ g^{-1}$) and Cu/OC (55.7 $m^2\ g^{-1}$), indicating that Ni doping was able to enhance the active site of base Cu, as evident defects can be observed on the enclosing carbon layer (yellow circles in* ***Figure 1****e and* ***Figure 2****d. $CuNi_{0.25}$@OC has a higher surface area (110.6 $m^2\ g^{-1}$) than $CuNi_{0.05}$@OC, but its pore volume and pore diameter are smaller than those of $CuNi_{0.05}$@OC, which may be due to pore blockage by the high doping amount of Ni. Notably, the multilevel pore structure of $CuNi_{0.05}$@OC with more pores and larger surface area not only facilitates the immobilization and dispersion of the active metal, but also provides more opportunities for substrate molecules to contact the active site, diffusion, and efflux.*

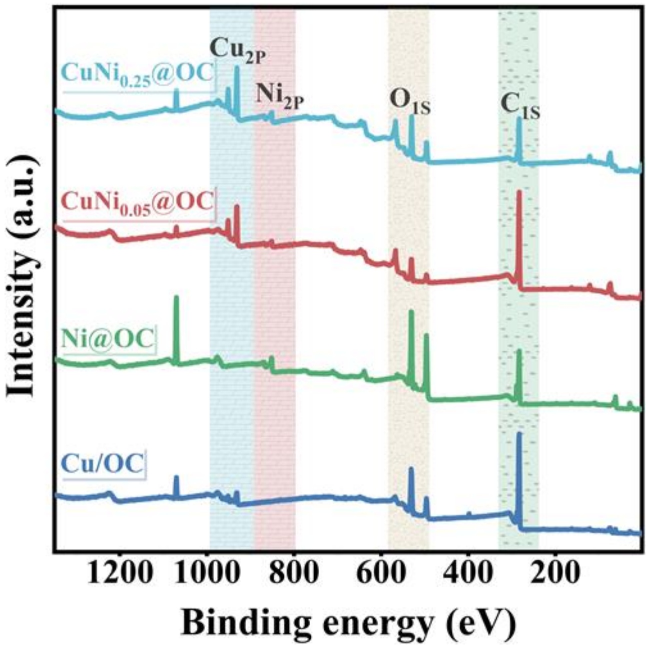

Figure S7. XPS survey spectra of various catalysts.

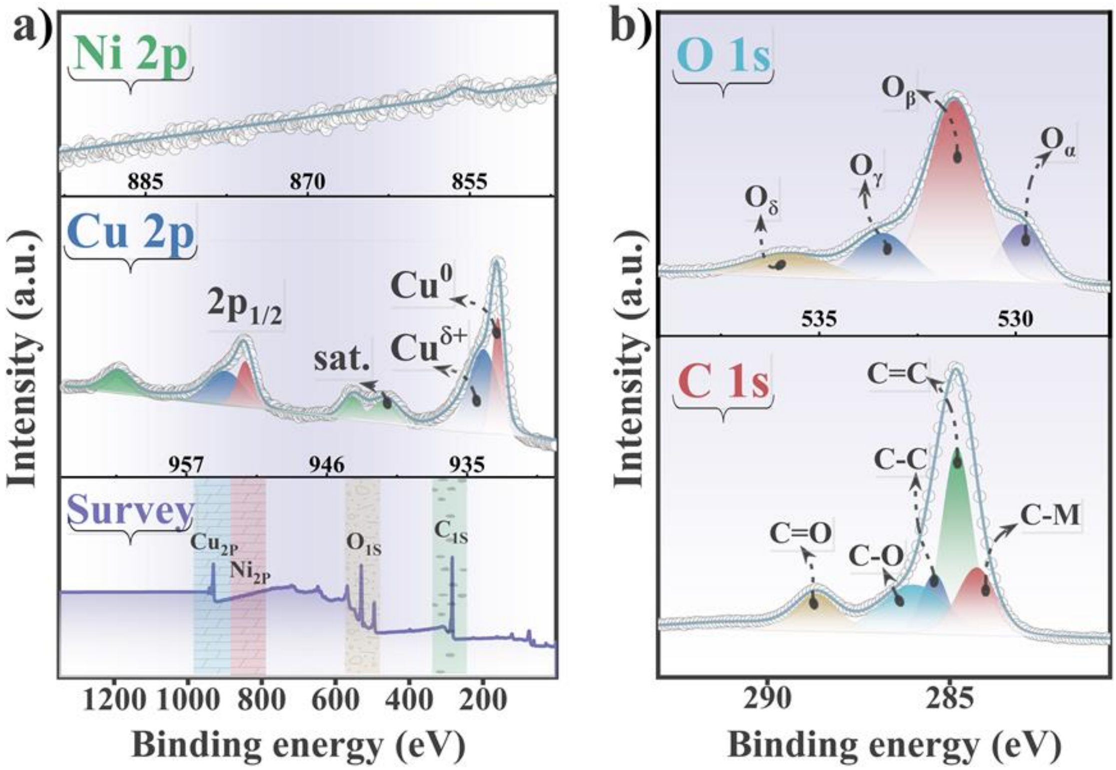

Figure S8. HR XPS spectra of $CuNi_{0.01}$@OC sample.

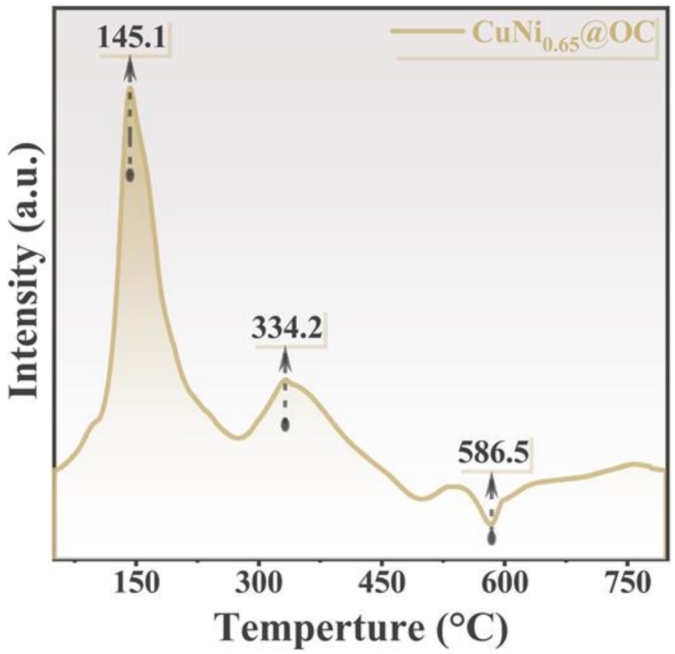

Figure S9. H_2-TPR profile of $CuNi_{0.65}$@OC sample.

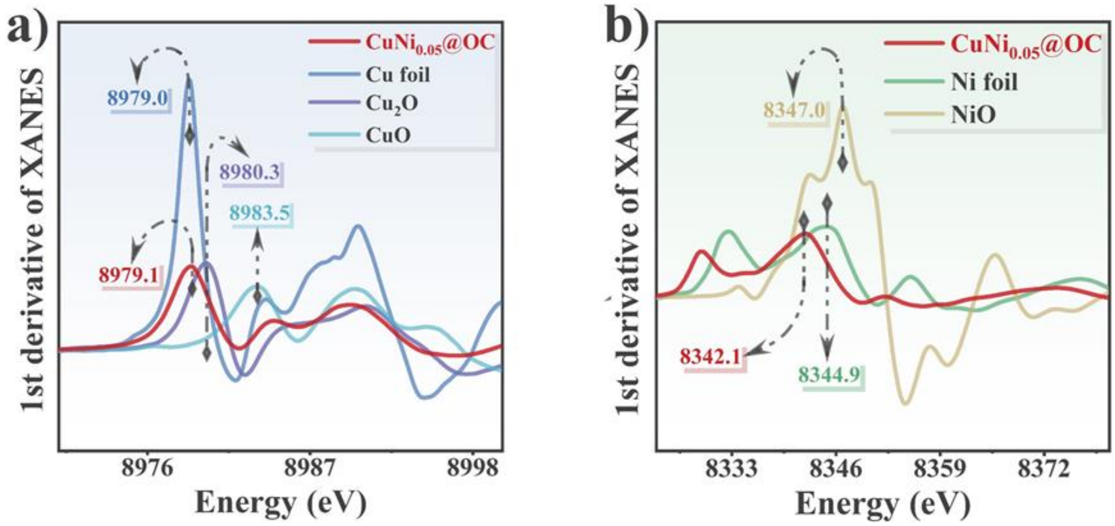

Figure S10. First derivatives of the **a)** Cu and **b)** Ni *K*-edge XANES region.

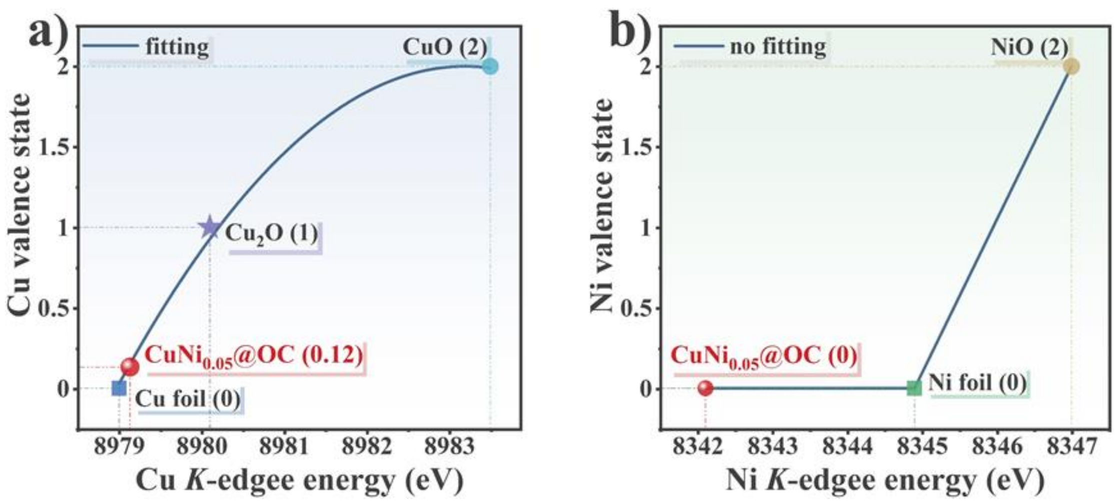

Figure S11. The valence state of various species obtained from **a)** Cu and **b)** Ni *K*-edge XANES spectra.

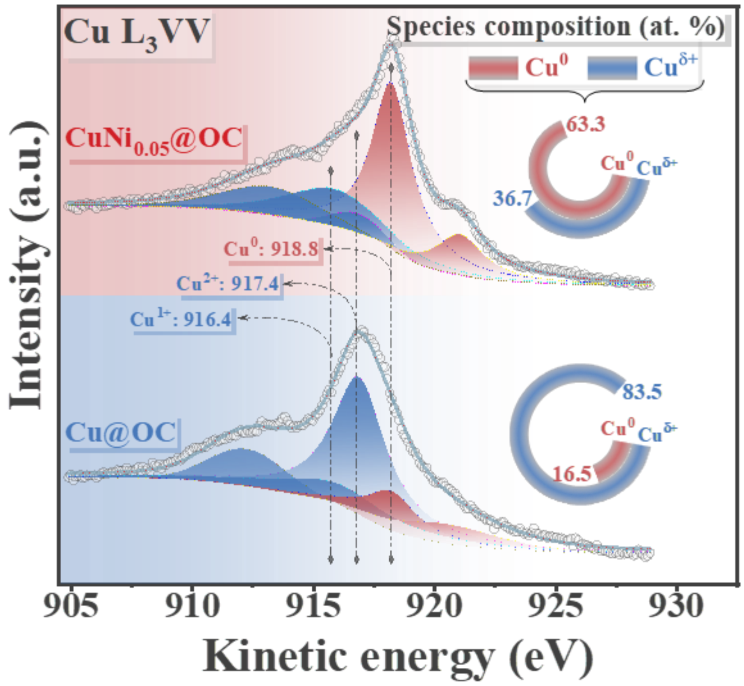

Figure S12. Cu L_3VV Auger spectra of $CuNi_{0.05}$@OC and Cu/OC sample.

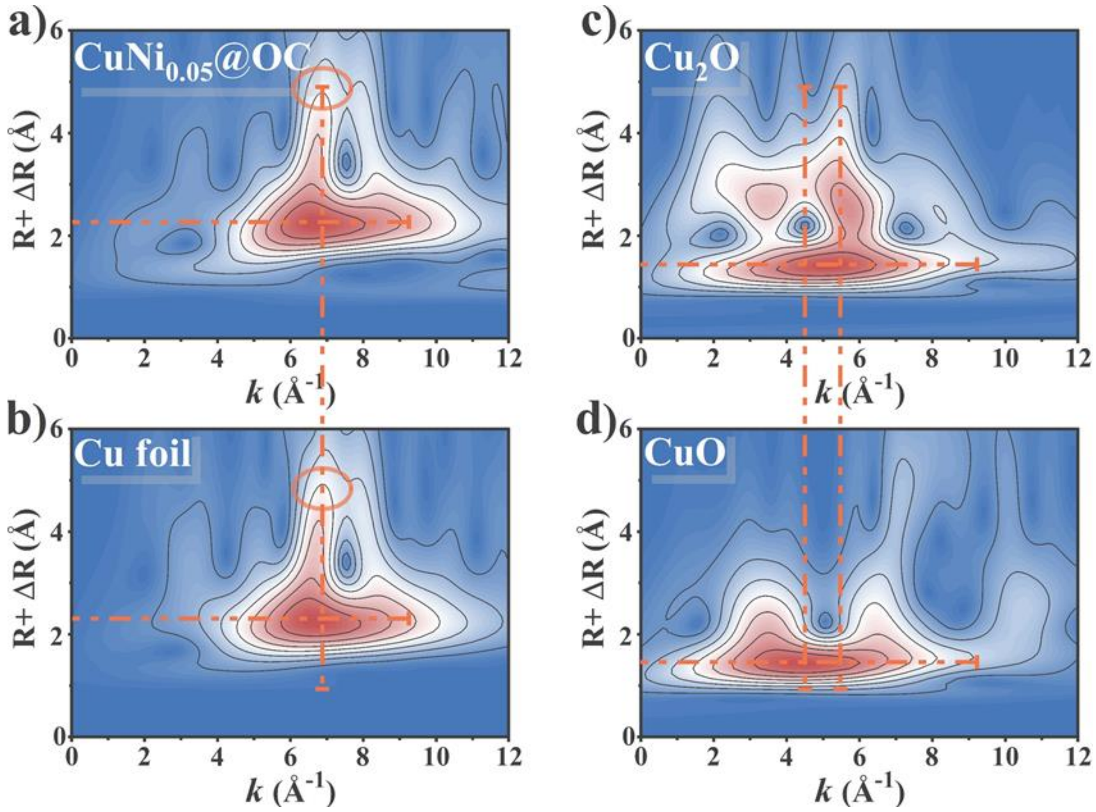

Figure S13. The EXAFS wavelet transform spectra of **a)** $CuNi_{0.05}$@OC, **b)** Cu foil, **c)** Cu_2O, and **d)** CuO, respectively.

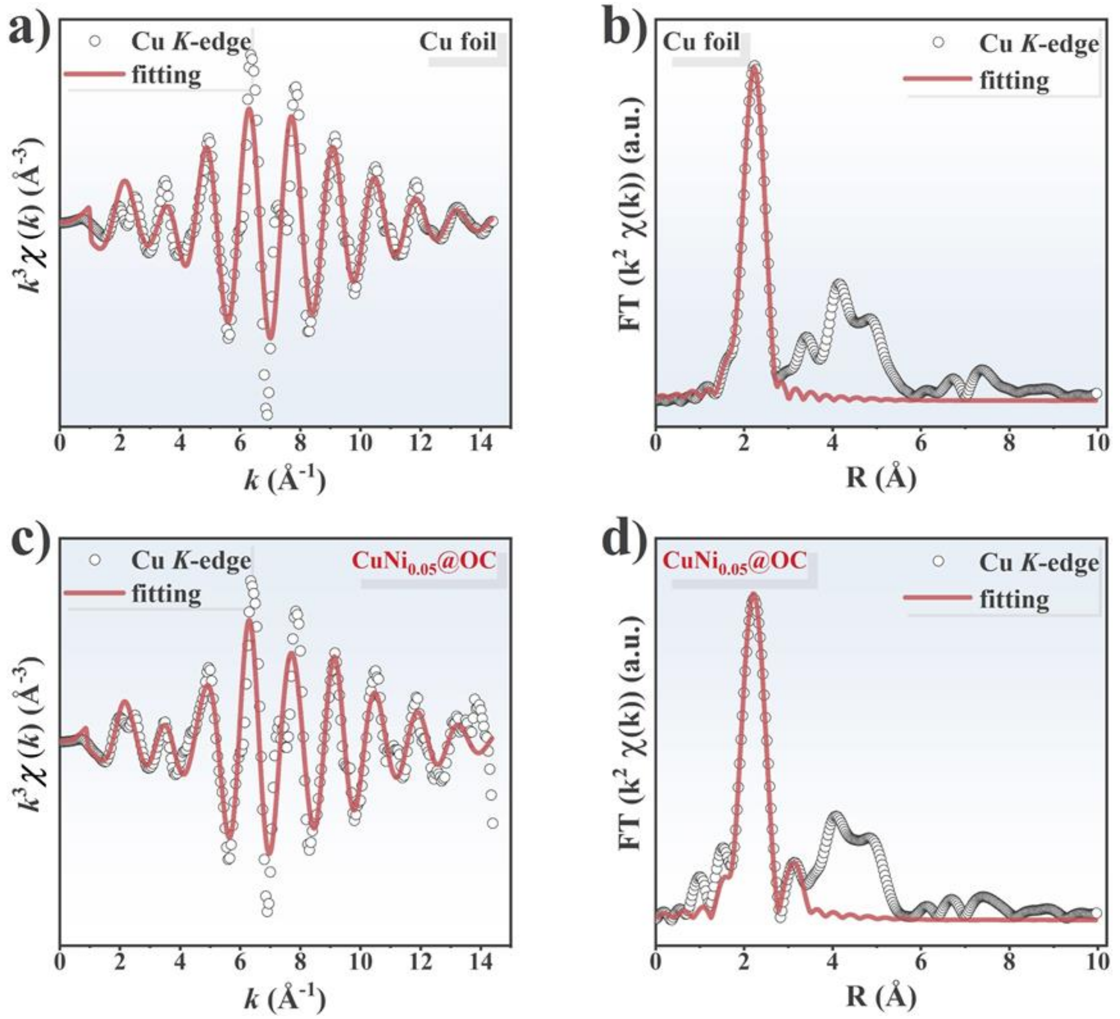

Figure S14. Cu *K*-edge EXAFS fitting curves of Cu foil and CuNi$_{0.05}$@OC: **a, b)** The Fourier transformation of filtered $\chi(k)\cdot k^3$; **c, d)** The radial structure functions obtained by Fourier transformation of k^3-weighted EXAFS results.

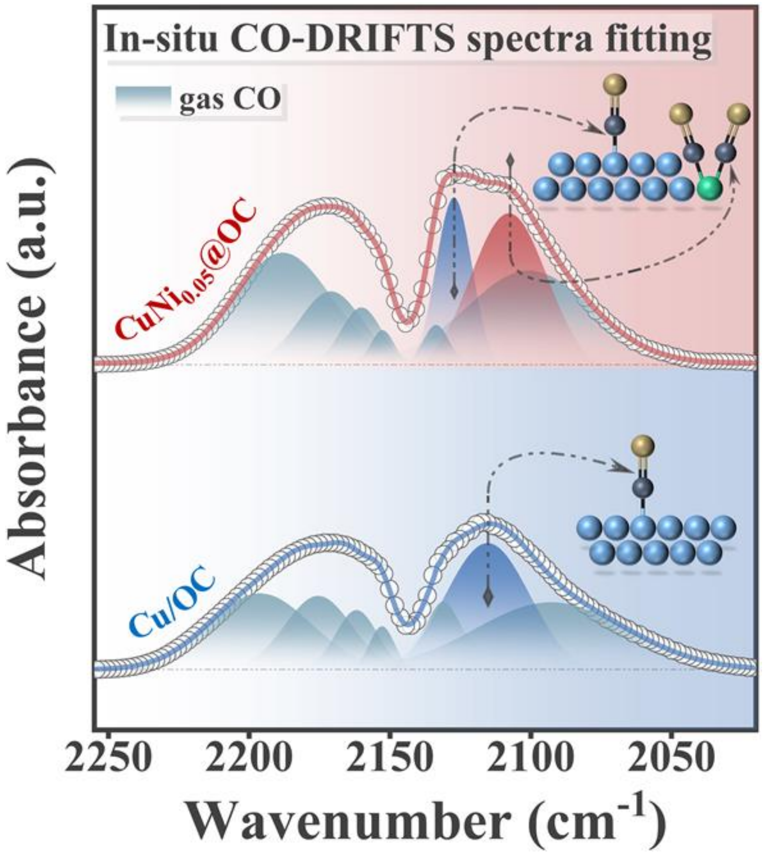

Figure S15. In-situ CO-DRIFTS spectra fitting of $CuNi_{0.05}$@OC and Cu/OC sample flushed with Ar for 5 min at room temperature. Cu: blue; Ni: green; C: gray; O: yellow.

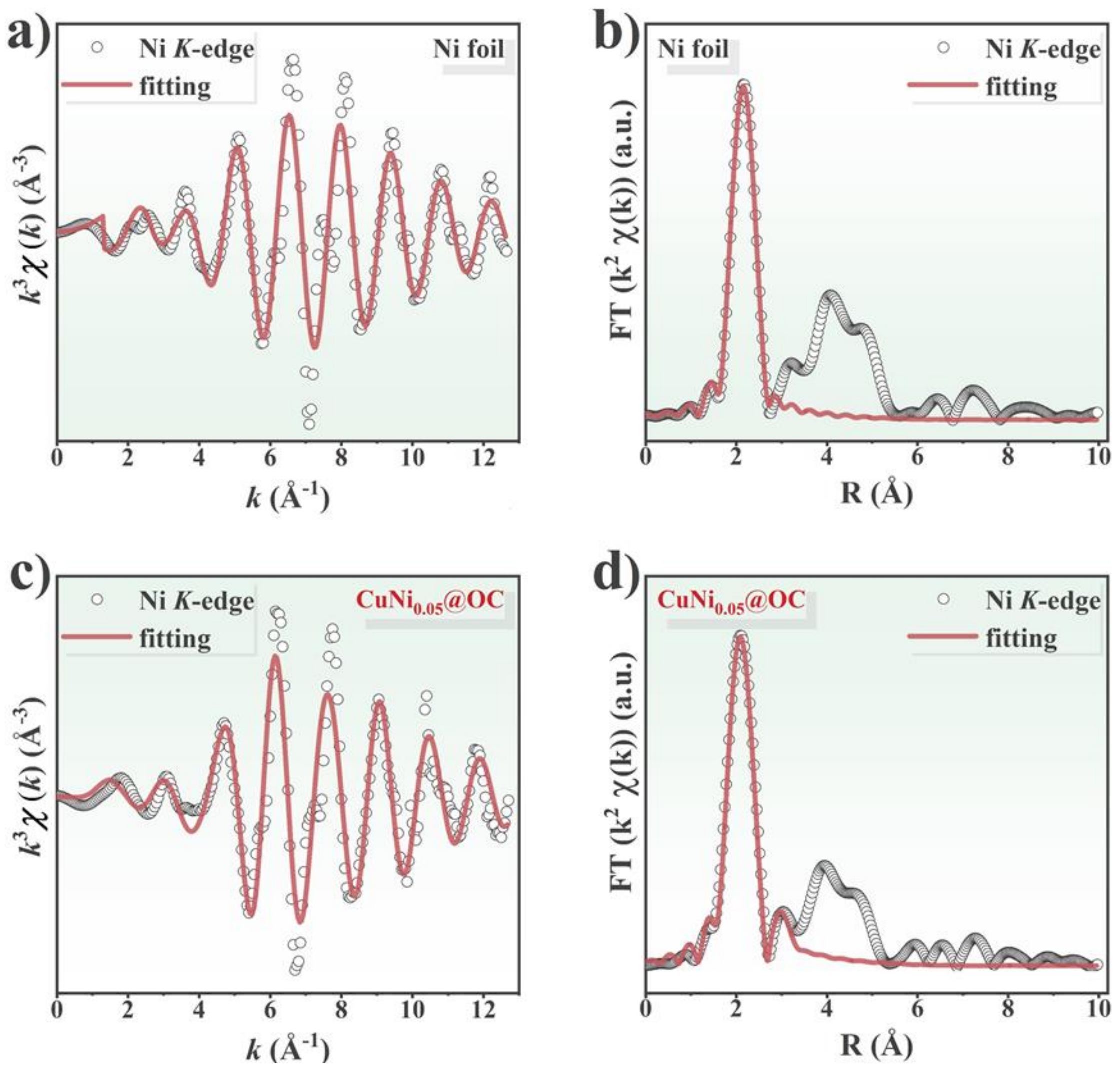

Figure S16. Ni *K*-edge EXAFS fitting curves of Ni foil and $CuNi_{0.05}$@OC: **a, b)** The Fourier transformation of filtered $\chi(k)\cdot k^3$; **c, d)** The radial structure functions obtained by Fourier transformation of k^3-weighted EXAFS results.

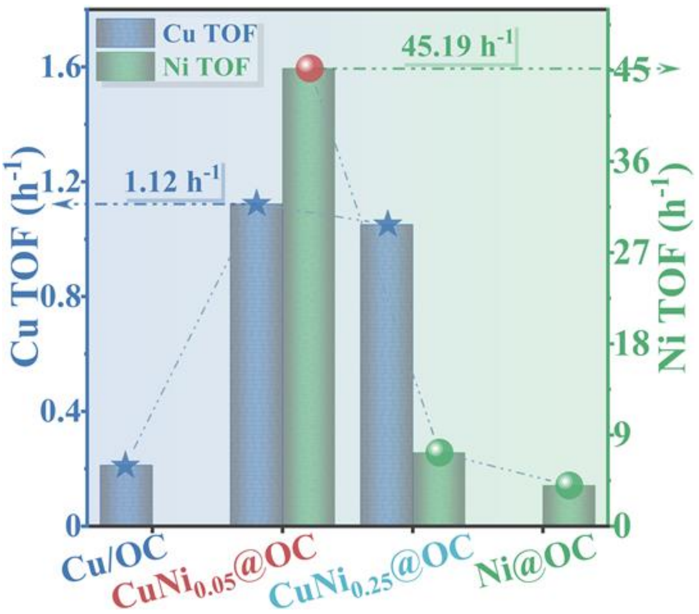

Figure S17. Turnover frequency based on Ni or Cu atoms for the various catalysts.

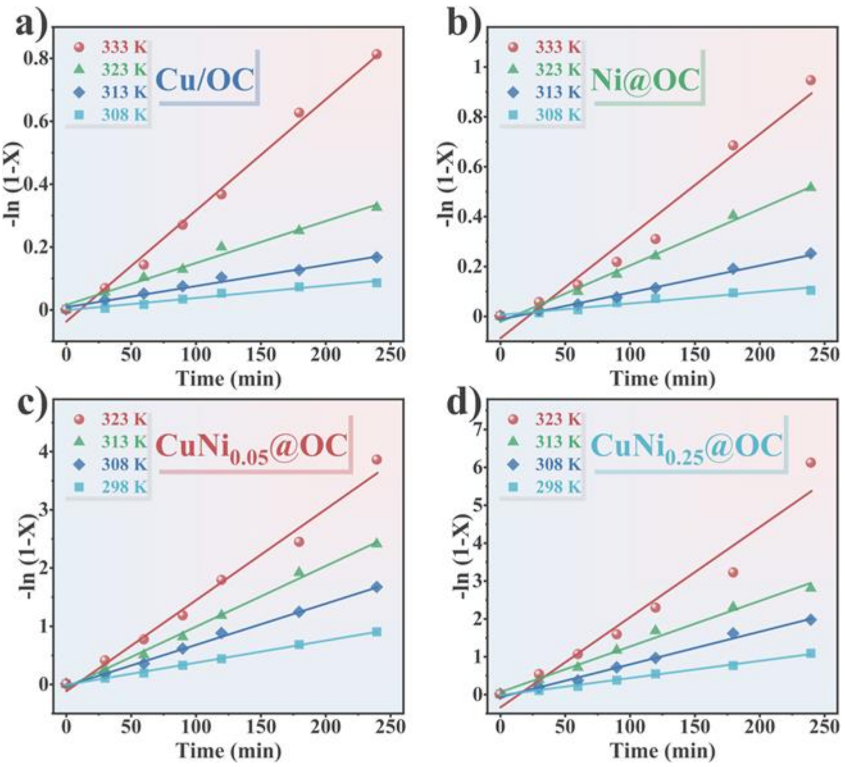

Figure S18. First-order kinetic fit for *p*-CNB hydrogenation to *p*-CAN at different temperatures. Reaction conditions: hydrazine hydrate (1.5 mmol); *p*-CNB (0.5 mmol); catalyst (20 mg); water (15 mL); 1000 rpm.

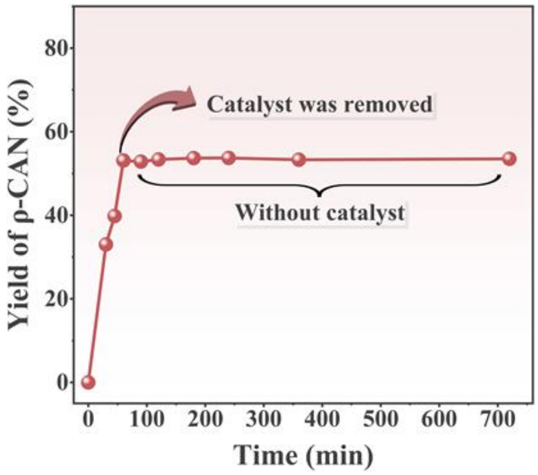

Figure S19. Hot filtration test for *p*-CNB hydrogenation. Reaction conditions: hydrazine hydrate (1.5 mmol); *p*-CNB (0.5 mmol); $CuNi_{0.05}$@OC catalyst (20 mg); water (15 mL); T =50 °C; 1000 rpm. Yields were determined by GC-MS.

After the catalyst was recovered from the solution by a magnet, leaving all the filtrate in the reacttion system, and the yield of p-CAN did not increase. The filtrate was analyzed and no metal was detected by ICP-AES, which proves that the reaction proceeded in a non-homogeneous manner.

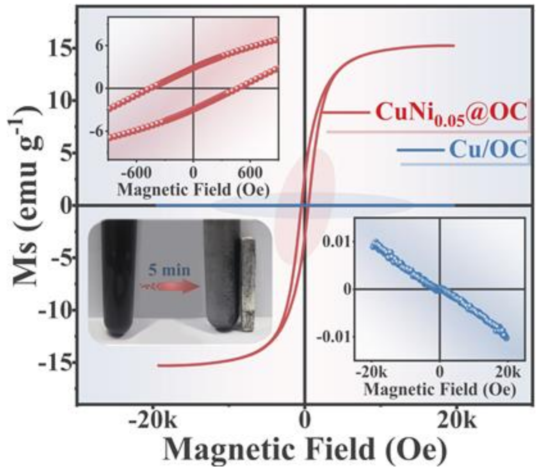

Figure S20. Magnetization curves at room temperature, inset in the upper left corner is magnified magnetic hysteresis loop from -900 to 900 Oe for $CuNi_{0.05}$@OC, in the lower right corner is magnified magnetic hysteresis loop from -20k to 20k Oe for Cu/OC, and in the lower left corner is photos of the $CuNi_{0.05}$@OC sample from dispersed in ultra-pure water to aggregation under external magnetic field.

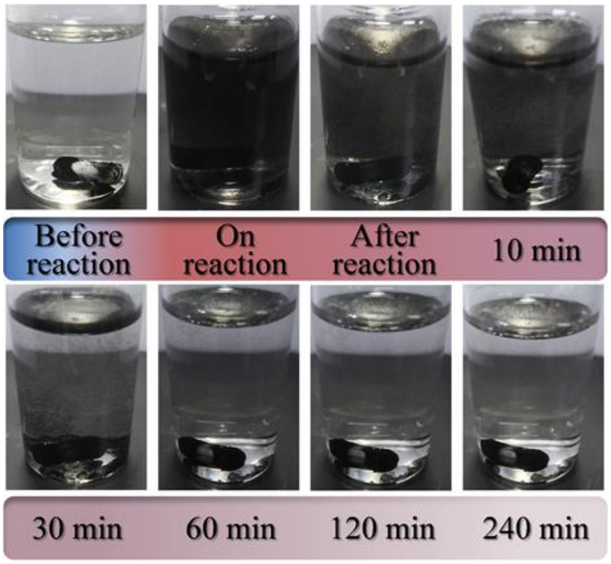

Figure S21. Photos of CTH of *p*-CNB using $CuNi_{0.05}$@OC at different stages of hydrogenation and recovery.

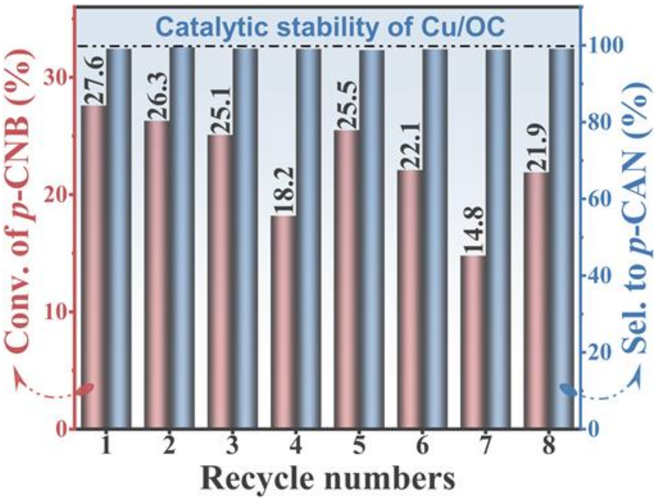

Figure S22. Reusability of Cu/OC in the consecutive cycles. Reaction conditions: hydrazine hydrate (1.5 mmol); *p*-CNB (0.5 mmol); catalyst (20 mg); water (15 mL); T =50 °C; 4 h; 1000 rpm.

After repeated use for 4 cycles, the activity of Cu/OC decreased from 27.6 % to 18.2 %. Extending the reaction time to 8.0 h promoted the conversion of the 5th and 6th cycles. The yield obtained from the 7th run was only 14.8 % albert by prolonging the reaction time to 12.0 h. After reduction and regeneration of the catalyst after the 7th operation, the yield was still low of 21.9 %.

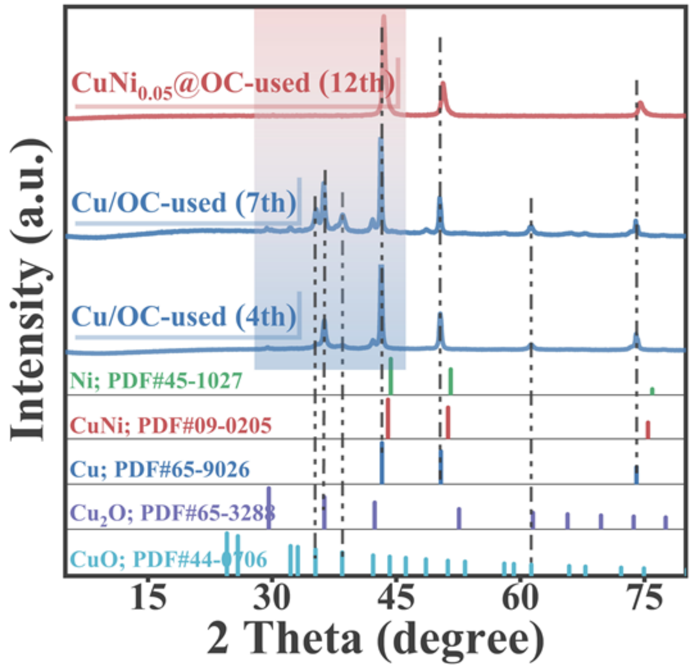

Figure S23. The XRD patterns of used catalysts.

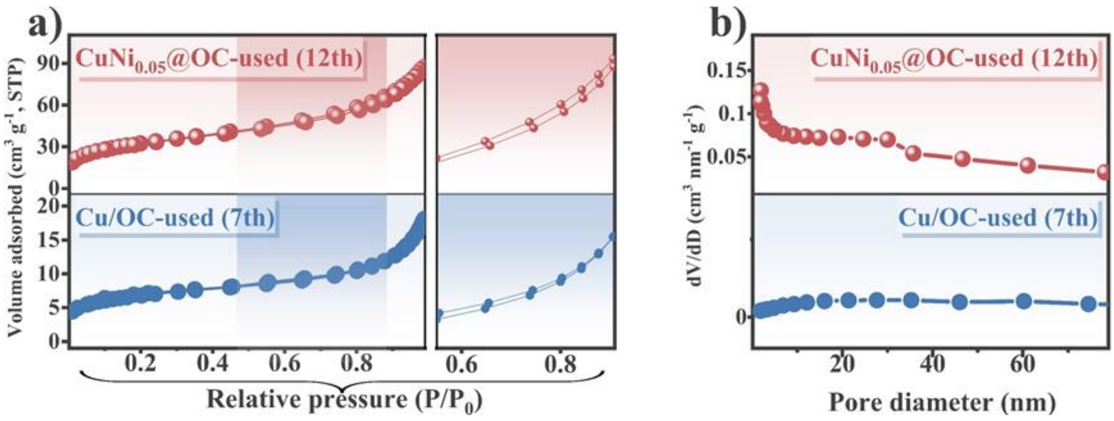

Figure S24. a) The N_2 adsorption-desorption isotherms and **b)** the BJH pore size distribution curves of used catalysts.

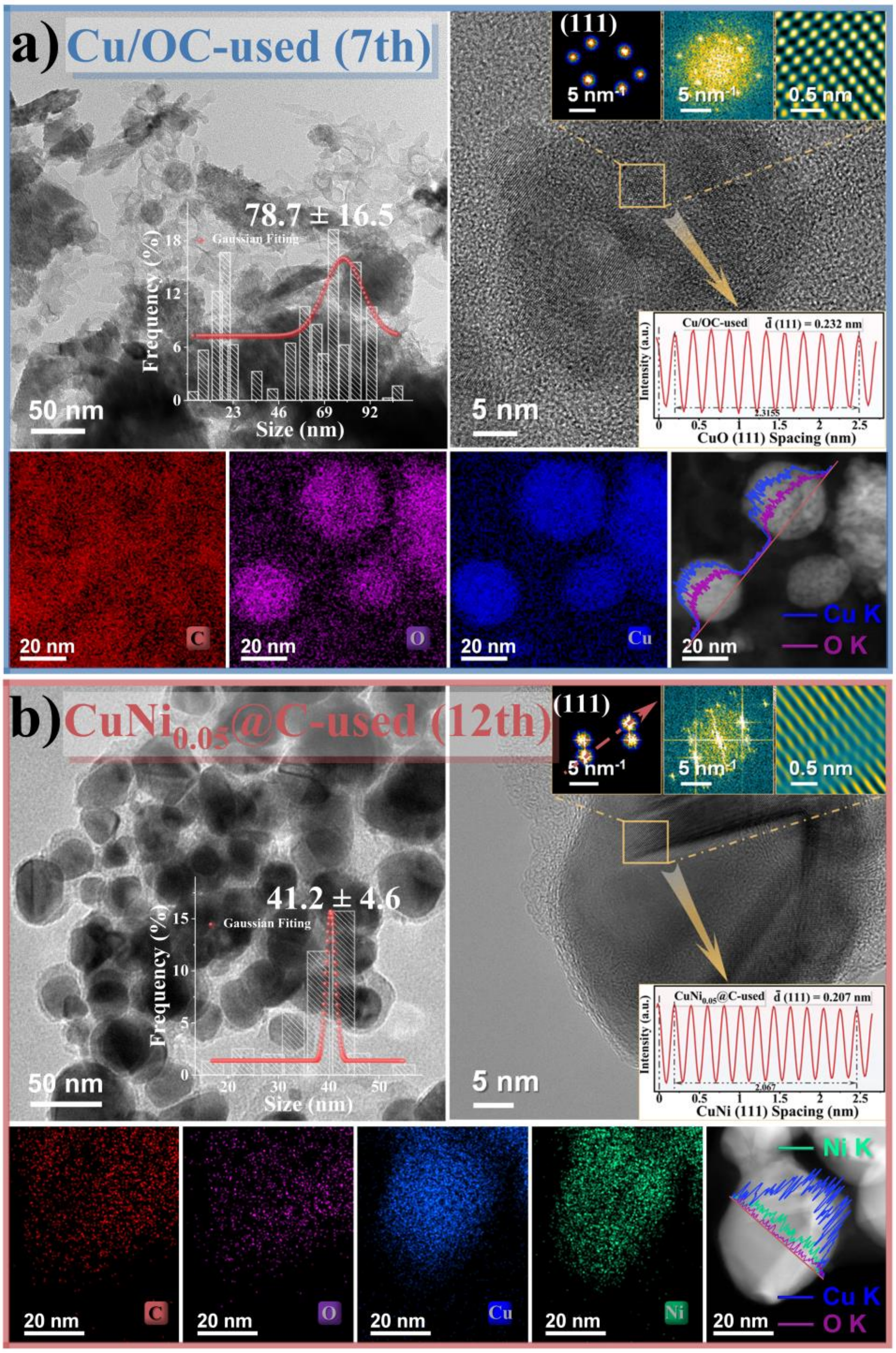

Figure S25. The HR-TEM images of used catalysts.

The Cu/OC catalyst showed obvious sintering during usage, with the NP size increase from the initial 62.9 nm (Figure S5a) to 78.7 nm after 7 cycles. On the other hand, the $CuNi_{0.05}$@OC catalyst almost did not change during repeated usage, with the NP size kept from the initial 40.6 nm to only slightly larger of 41.2 nm even after 12 cycles.

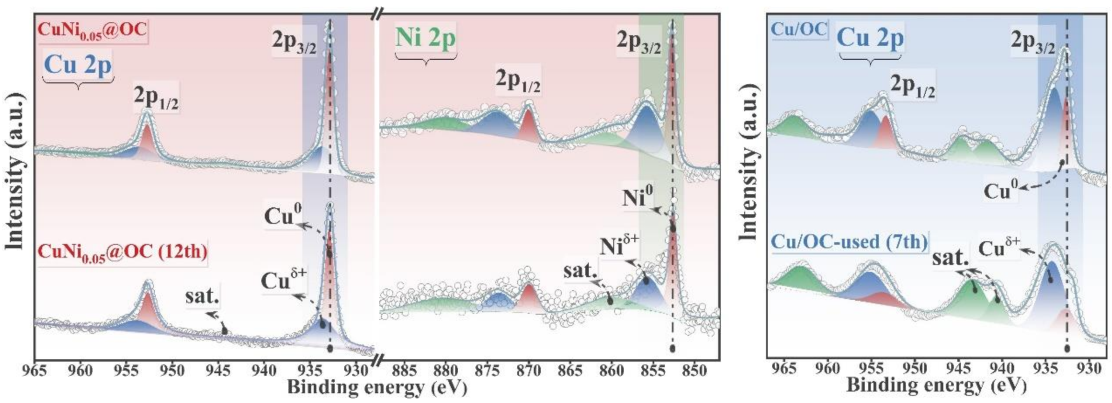

Figure S26. HR-XPS spectra of used $CuNi_{0.05}$@OC and Cu/OC catalysts in comparison with those of the fresh ones.

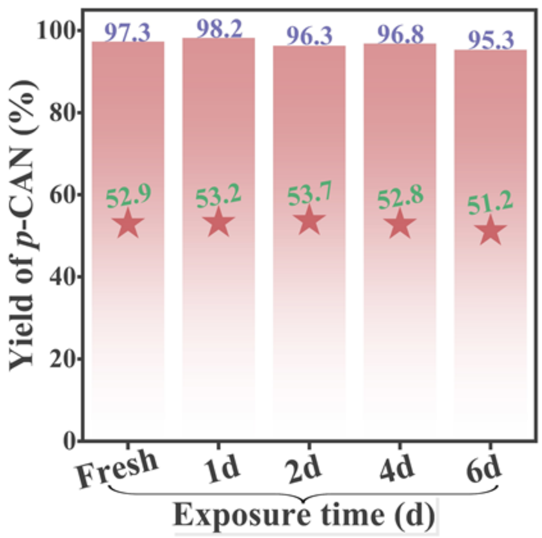

Figure S27. Stability tests of $CuNi_{0.05}$@OC in CTH of *p*-CNB to *p*-CAN after air exposure. Reaction conditions: hydrazine hydrate (1.5 mmol); *p*-CNB (0.5 mmol); catalyst (20 mg); water (15 mL); T =50 ℃; 1000 rpm. Reaction time: 4 h (red bars), 1 h (red stars).

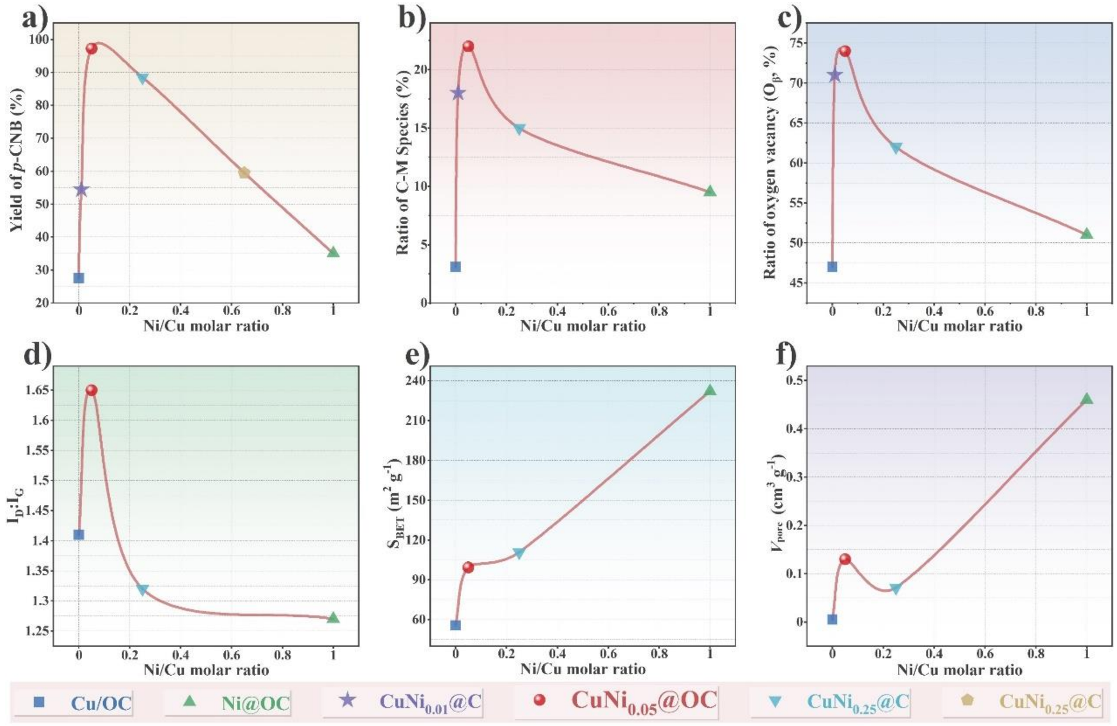

Figure S28. Effect of Ni/Cu molar ratio on the catalytic performance and physicochemical property of the series catalysts including Cu/OC, $CuNi_{0.01}$@OC, $CuNi_{0.05}$@OC, $CuNi_{0.25}$@OC, $CuNi_{0.65}$@OC, and Ni@OC: **a)** *p*-CNB yield; **b)** ratio of C-M species; **c)** ratio of oxygen vacancy; **d)** I_D/I_G in the carbon coating; **e)** surface area; **f)** pore volume.

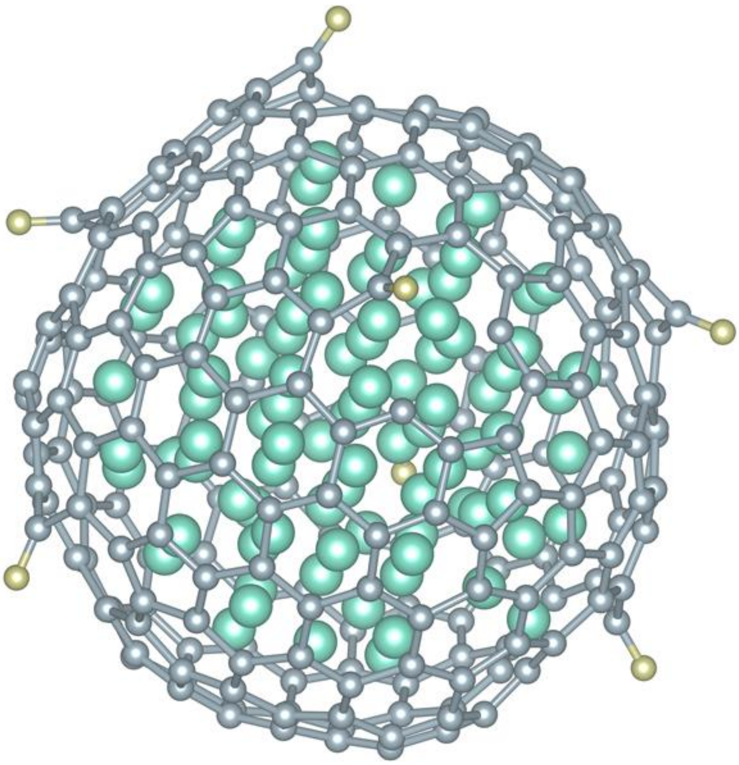

Figure S29. Geometric configurations of Ni (111) surfaces. Ni: green; C: gray; O: yellow.

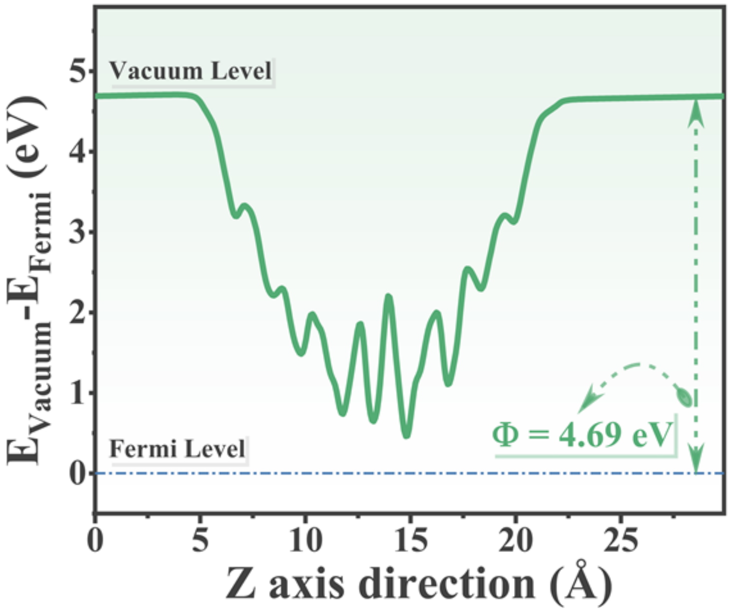

Figure S30. Calculated work function (Φ) of Ni (111) surfaces.

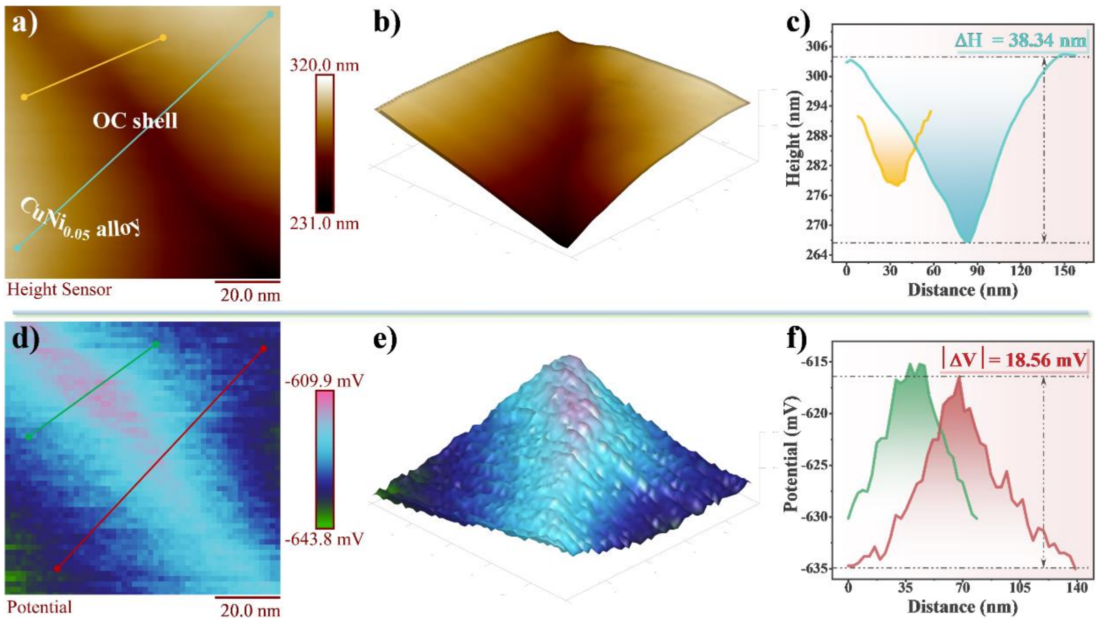

Figure S31. a) AFM image of $CuNi_{0.05}$@OC, **b)** the corresponding 3D image, and **c)** height profile of the yellow and cyan lines in panel (a). **d)** Surface potential distribution of $CuNi_{0.05}$@OC under dark irradiation, **e)** the corresponding 3D image, and **f)** potential profile of the greed and red lines in panel (d).

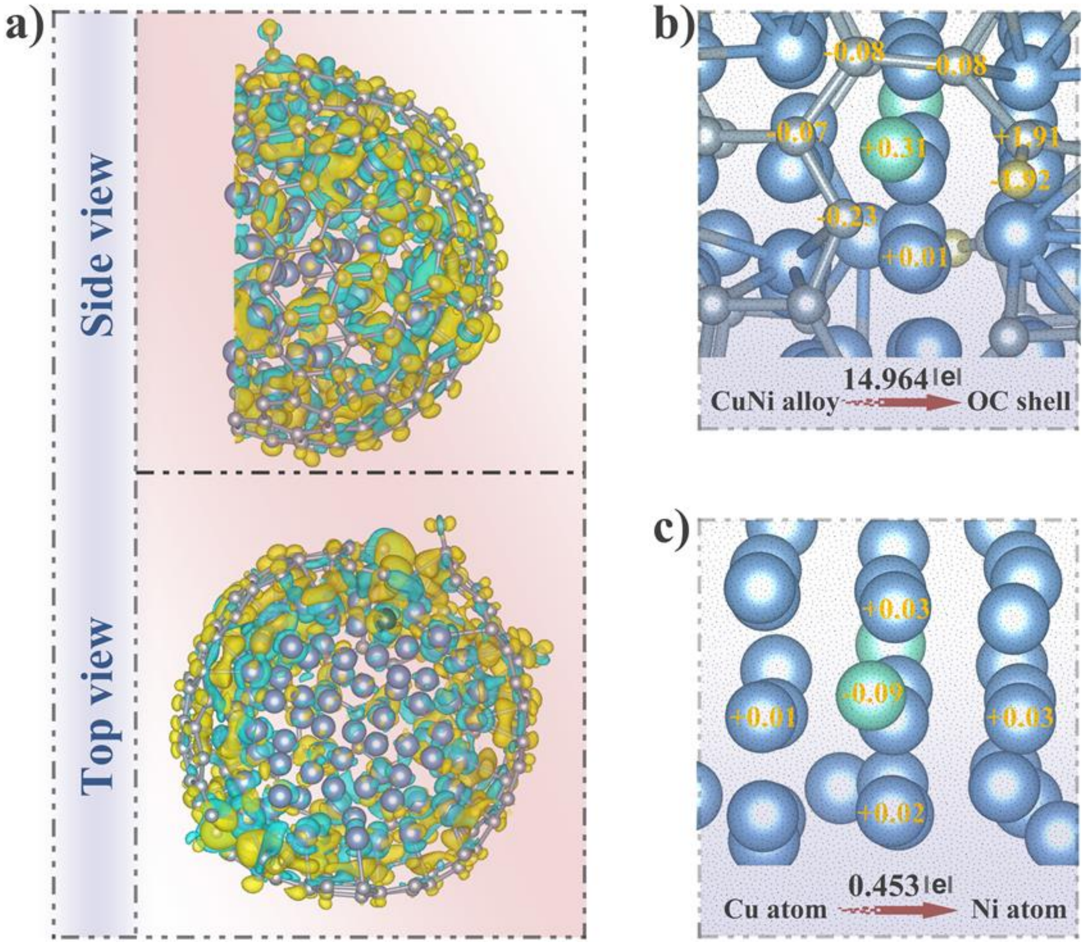

Figure S32. a) Charge density differential analysis of $CuNi_{0.05}$@OC presented in half-section for a clearer representation of electron migration. The yellow and blue contours indicate electron increment and depletion, respectively. Cu: blue; Ni: green; C: gray; O: yellow; same below. **b)** Bader charge analysis of $CuNi_{0.05}$@OC. The numbers stand for the amount of charge carried by corresponding atoms of pristine $CuNi_{0.05}$@OC system, which reveal that the outer oxygen-doped carbon (OC) shell carries negative charges from the internal Cu-Ni dilute alloy. **c)** Bader charge analysis of Cu-Ni dilute alloy. The numbers stand for the amount of charge carried by corresponding atoms of pristine CuNi(111) surfaces, which reveal that isolated Ni atom carries negative charges from sub-surface Cu atoms.

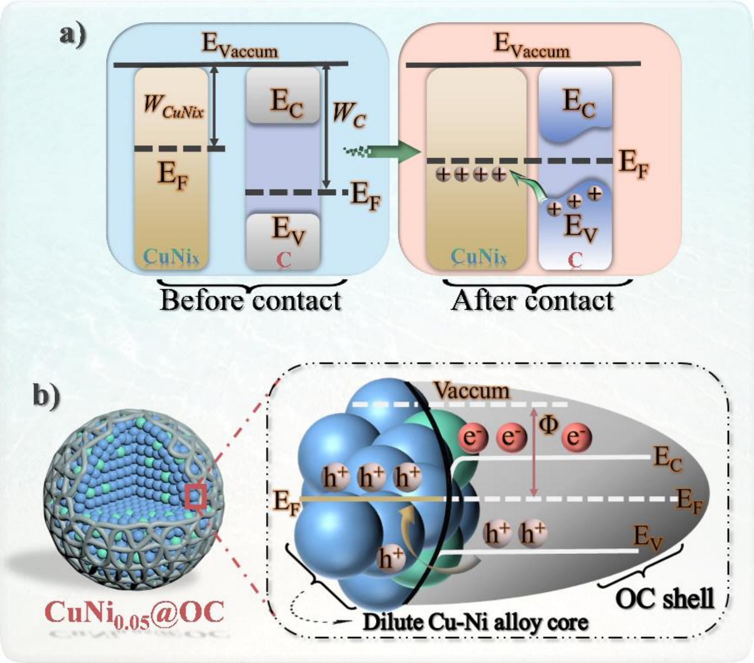

Figure S33. a) The Mott-Schottky type contact of $CuNi_x$@OC catalysts. **b)** The Mott-Schottky contacting interface of diluted alloy $CuNi_{0.05}$ NPs and OC shell of $CuNi_{0.05}$@OC samples. Cu: blue; Ni: green; OC: grey.

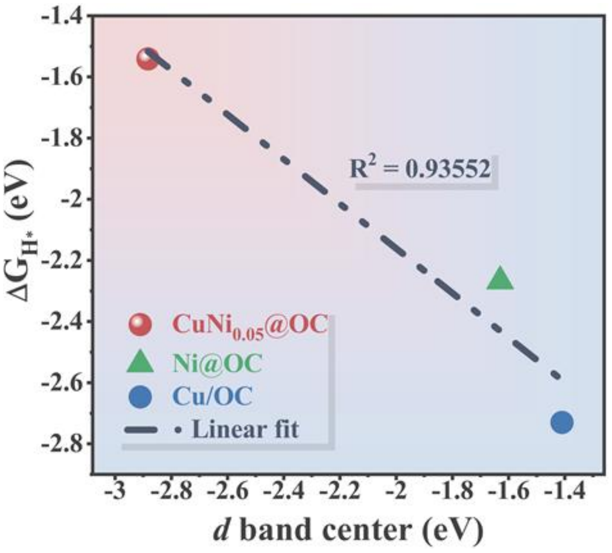

Figure S34. Relationship between d-band center and ΔG_{H^*} values over various catalyst models.

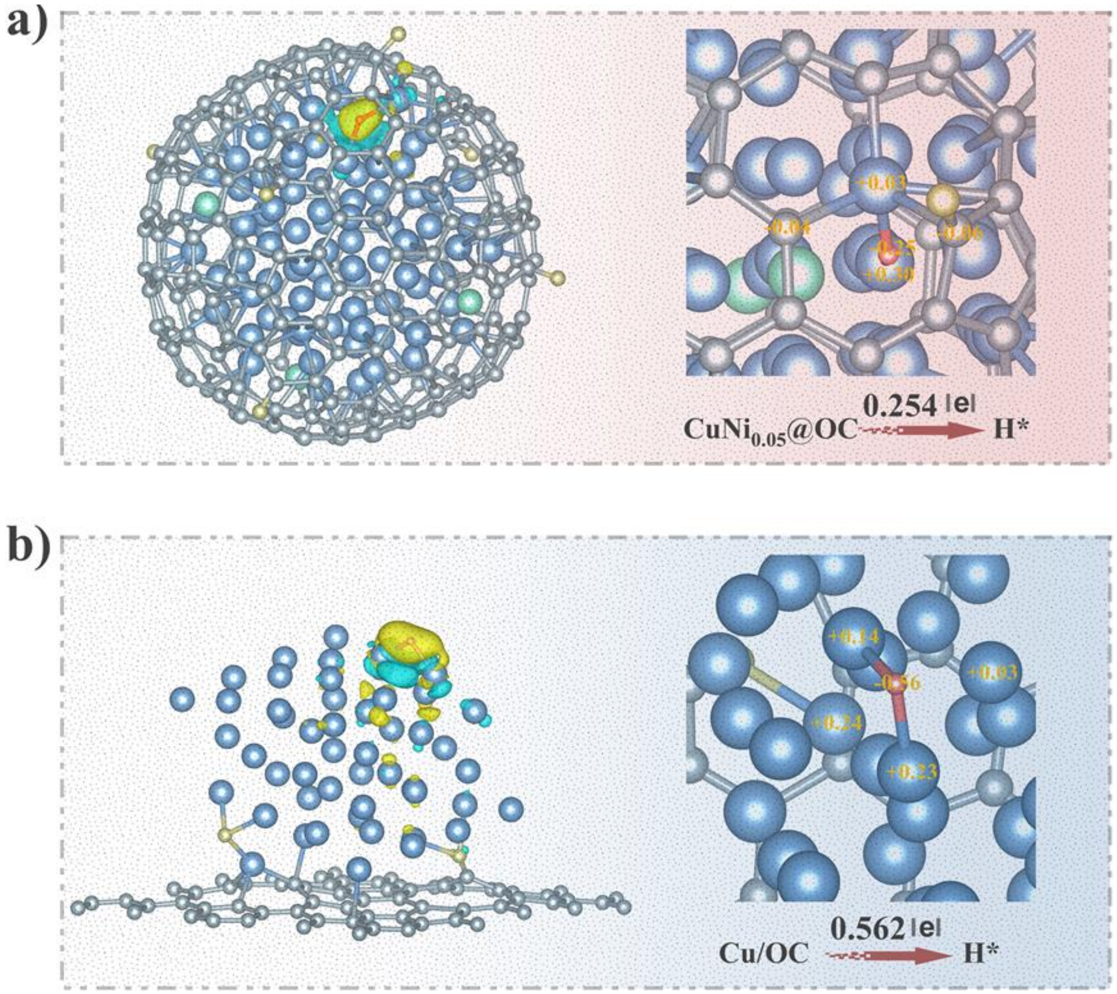

Figure S35. Differential charge density diagrams of H-adsorption state for **a)** $CuNi_{0.05}$@OC and **b)** Cu/OC models.

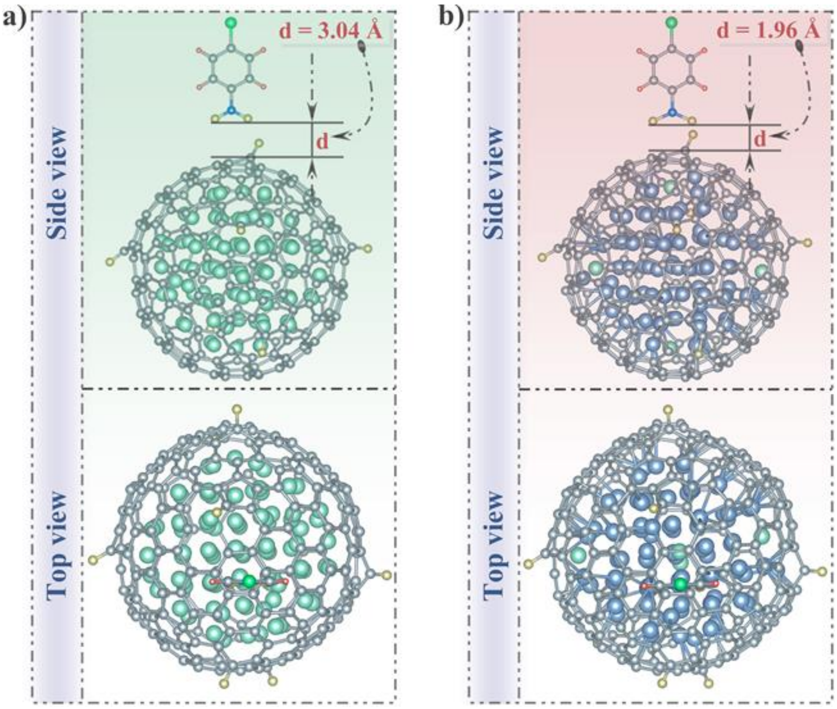

Figure S36. Top and side views of the optimized structure of *p*-CNB molecule on **a)** Ni@OC and **b)** $CuNi_{0.05}$@OC. The letter d represents the adsorption distance between *p*-CNB molecule and substrate.

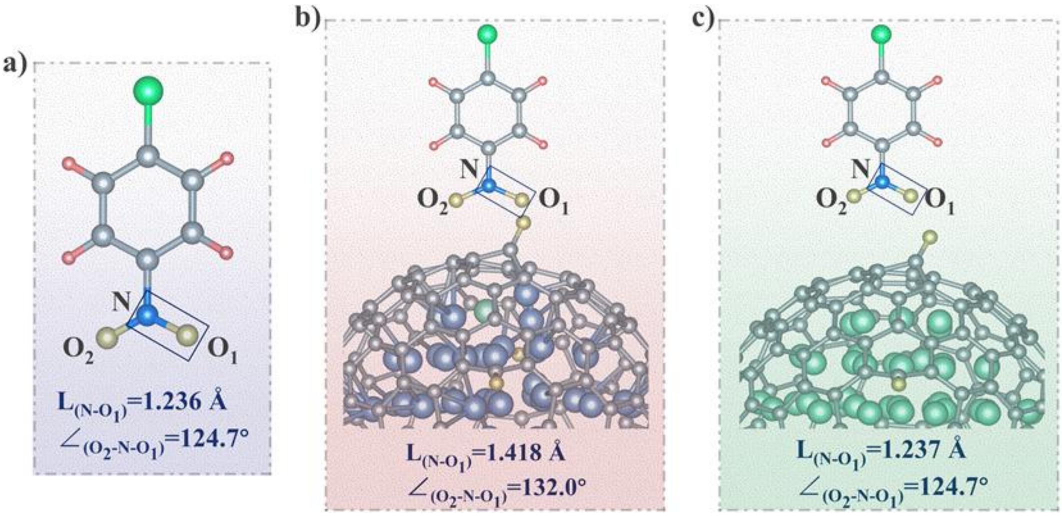

Figure S37. a) Structural diagram of *p*-CNB. Molecular structure of gaseous *p*-CNB, with the labeled bond length (L(N-O_1)) and bond angle (∠(N-O_1)) of N-O_1. Correspondingly, the bond lengths and bond angles of N-O_1 of *p*-CNB after adsorption on the Ni@OC **b)** and CuNi@OC **c)**. C: orange; O: yellow; N: blue; H: white.

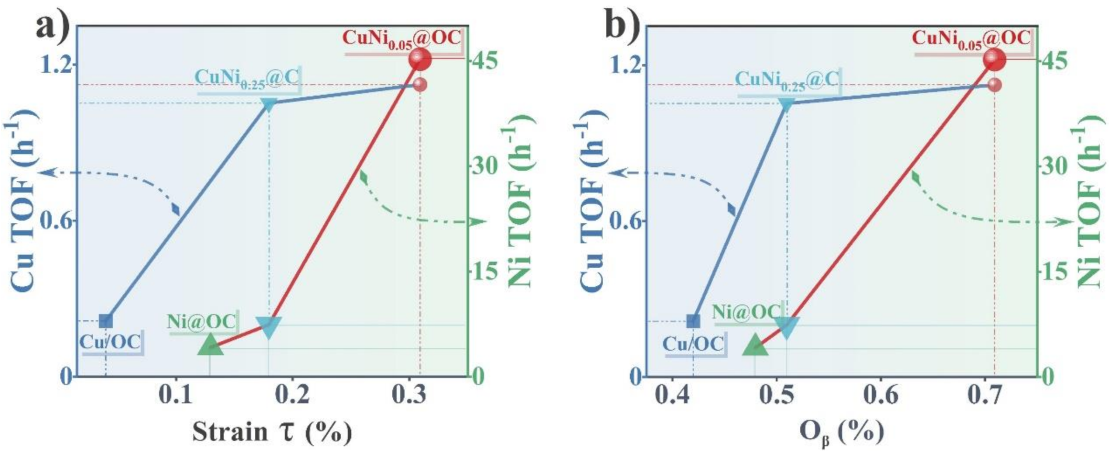

Figure S38. The positive correlation relationship between TOF value and **a)** τ, and **b)** the content of Oβ.

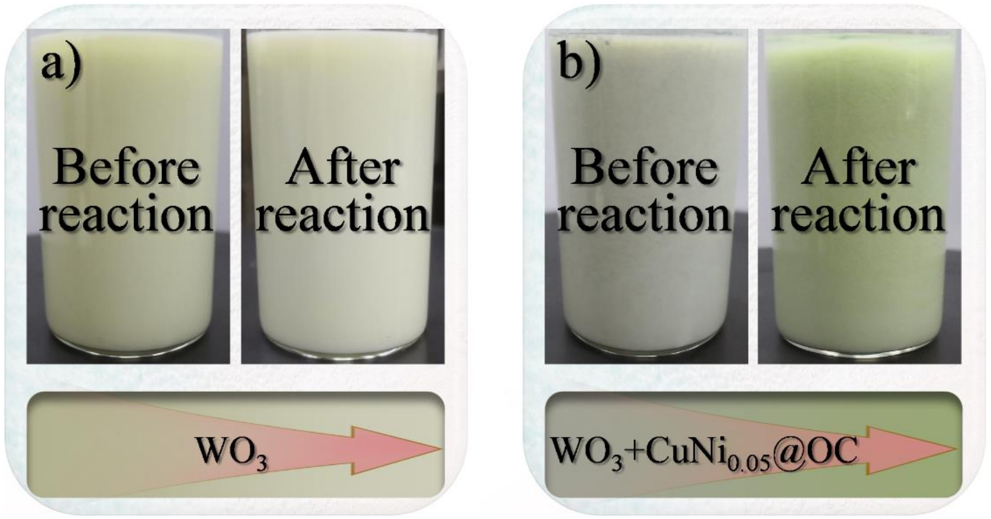

Figure S39. Photographs of reaction mixture containing **a)** 1 g WO_3 and **b)** mixture of 1 g WO_3 and 20 mg $CuNi_{0.05}$@OC before and after hydrogenation. Reaction conditions: hydrazine hydrate (1.5 mmol); *p*-CNB (0.5 mmol); water (15 mL); T =50 °C; 4 h; 1000 rpm.

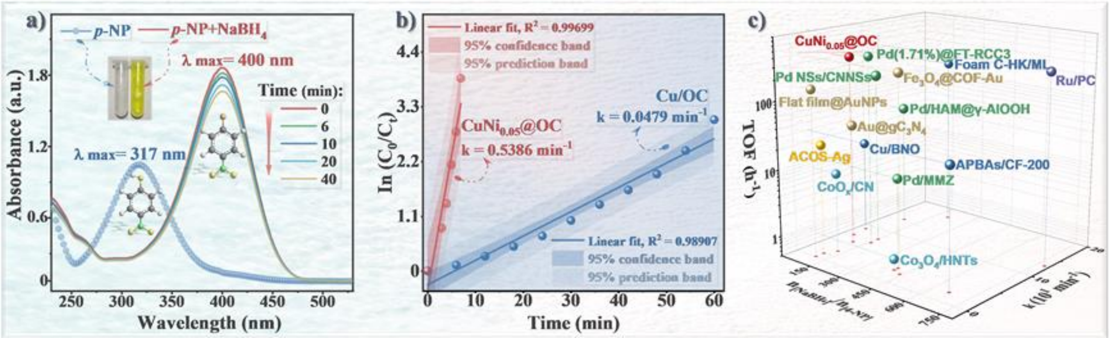

Figure S40. a) UV-vis absorption spectra of *p*-NP before and after addition of excessive $NaBH_4$, and UV-vis intensity of *p*-NP aqueous solution in the presence of only $NaBH_4$ and the carbon carrier recorded at different intervals. **b)** Plot of ln (C_0/C_t) vs. time. **c)** Comparison of the CTH conversion of *p*-NP to *p*-AP over different catalysts (see **Table S10** for details). Reaction conditions: $NaBH_4$ (0.2 M); *p*-NP (10 mM); catalyst (2 mg); T =25 °C; 1000 rpm.

Catalytic reduction of p-AP by p-NP has great commercial value: on the one hand, it can deal with the pollution caused by the accumulation of p-NP, a non-biodegradable pollutant, in water; On the other hand, p-AP with high added value can be obtained, which can be used in medicine, pesticide, rubber, dye and other industries. As shown in ***Figure S40****a, when* $NaBH_4$ *was added to p-NP, a yellow aqueous solution and the absorption maximum at 400 nm were initially formed, which confirmed the formation of p-nitrophenolate ion (p-NP*$^-$*).*[9] *After the carbon carrier was introduced into the yellow solution, the time was prolonged to 40 min, and the peak intensity at 400 nm decreased slightly, but no new peak appeared at 295 nm, which proved that the carbon carrier was not a suitable catalyst, but it had a certain adsorption effect on p-NP*$^-$*.*

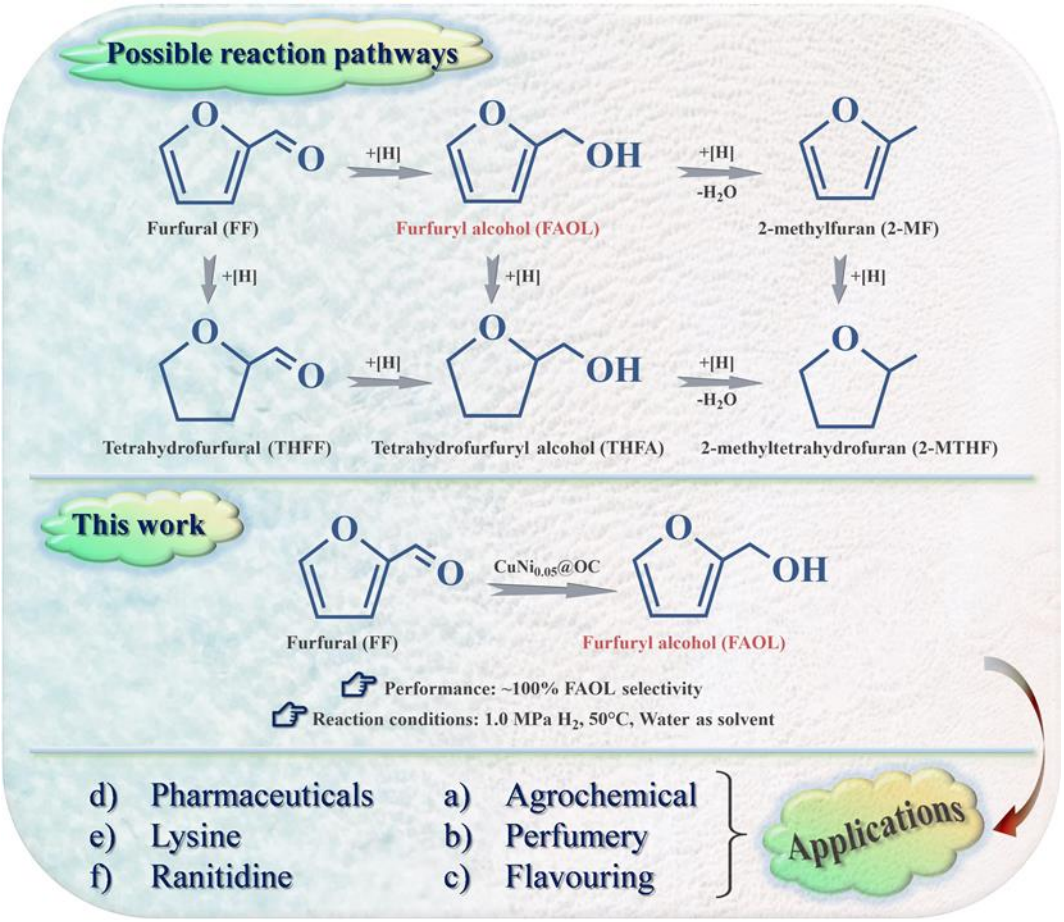

Figure S41. Schematic illustration of FF hydrogenation pathways, our work in selective carbonyl hydrogenation of FF toward FAOL, and applications of hydrogenated products in different engineering aspects.

Table S1. The ICP-AES of the different samples.

Samples	W_{Cu} (wt.%) [a]	W_{Ni} (wt.%) [a]	Bulk Ni/Cu molar ratio [b]
Cu/OC	58.62	-	-
Ni@OC	-	1.86	-
$CuNi_{0.01}$@OC	55.89	0.39	0.01
$CuNi_{0.05}$@OC	57.99	1.82	0.05
$CuNi_{0.25}$@OC	58.93	8.52	0.25
$CuNi_{0.65}$@OC	59.21	11.91	0.65
$CuNi_{0.05}$@OC-used (12th)	56.55	1.77	0.05
Cu/OC-used (7th)	41.87	-	-

[a] W_{Cu} *or* W_{Ni}, determined by ICP-AES. [b] The metal molar ratio.

Table S2. Textural properties of the samples.

Samples	S_{BET} [a] (m^2g^{-1})	V_{pore} [b] (cm^3g^{-1})	D_{pore} [c] (nm)
Cu/OC	55.7	0.0052	7.95
Ni@OC	232.01	0.46	8.01
$CuNi_{0.05}$@OC	99.38	0.13	5.68
$CuNi_{0.25}$@OC	110.61	0.071	2.41
$CuNi_{0.05}$@OC-used (12th)	108.57	0.13	6.93
Cu/OC-used (7th)	22.31	0.0034	5.07

[a] Surface area derived from BET equation. [b] Pore volume, obtained from the volume of nitrogen adsorbed at the relative pressure of 0.98. [c] Pore size, derived from BJH method using adsorption branch.

Table S3. XPS characterization data of the different catalysts.

Samples	BE of C 1s (eV)					BE of O 1s (eV)				BE of Cu $2p_{3/2}$ (eV)		BE of Ni $2p_{3/2}$ (eV)		XPS comp. (at. %)		Surface Ni/Cu atomic ratio	Species composition (/%)			
	C=O	C-O	C-C	C=C	C-M	$O_δ$ [a]	$O_γ$ [a]	$O_β$ [a]	$O_α$ [a]	$Cu^{δ+}$	Cu^0	$Ni^{δ+}$	Ni^0	Cu	Ni		C-M	$O_β$	Cu^0	Ni^0
Cu/OC	289.3	286.8	285.4	284.8	284.7	536.6	533.3	531.8	529.8	933.8	932.5	-	-	1.75	-	-	0.031	0.47	0.29	-
Ni@OC	290.3	287.2	285.7	284.8	284.6	536.4	533.2	532.0	530.8	-	-	856.1	853.1	-	3.07	-	0.095	0.51	-	0.47
$CuNi_{0.01}$@OC	288.7	286.1	285.4	284.8	284.3	535.7	533.4	531.6	529.9	933.7	932.6	-	-	5.08	0.38	0.07	0.18	0.71	0.37	-
$CuNi_{0.05}$@OC	288.2	286.5	285.5	284.8	284.2	535.7	533.6	531.9	529.8	933.7	932.9	855.7	852.7	4.21	1.37	0.33	0.22	0.74	0.51	0.45
$CuNi_{0.25}$@OC	288.8	286.4	285.4	284.8	284.3	536.0	532.7	531.6	529.9	933.7	932.8	855.4	852.7	8.46	3.63	0.43	0.15	0.62	0.62	0.37

[a] $O_α$, $O_β$, $O_γ$, and $O_δ$ defined as lattice oxygen, oxygen vacancy, surface adsorbed oxygen, and adsorbed water, respectively.

Table S4. EXAFS fitting parameters at the Cu or Ni *K*-edge for various samples.

Sample		Shell	*CN* [a]	*R* (Å) [b]	σ^2 (Å^2) [c]	ΔE_0 (eV) [d]	*R* factor [e]
Cu *K*-edge (S_0^2 = 0.81)	Cu foil	Cu-Cu	12 [f]	2.53±0.011	0.00079	4.85±1.02	0.004
	Cu_2O	Cu-O	2 [f]	1.91±0.011	0.0049	7.01±1.57	0.005
	CuO	Cu-O	6 [f]	1.93±0.018	0.0059	3.19±0.70	0.006
		Cu-Cu	4 [f]	2.92±0.024	0.0066	6.71±1.51	
	$CuNi_{0.05}$@OC	Cu-Cu	7.0±0.3	2.52±0.017	0.0069	3.22±1.62	0.011
Ni *K*-edge (S_0^2 = 0.85)	Ni foil	Ni foil	12 [f]	2.46±0.021	0.0056	7.13±2.05	0.006
	NiO	Ni-O	6 [f]	2.15±0.022	0.0080	4.07±1.16	0.005
		Ni-Ni	12 [f]	2.94±0.016	0.0069	4.56±1.07	
	$CuNi_{0.05}$@OC	Ni-Cu	8.6±0.4	2.41±0.028	0.0057	4.53±0.69	0.012

[a] *CN*, coordination number; [b] *R*, distance between absorber and backscatter atoms; [c] σ^2, Debye-Waller factor to account for both thermal and structural disorders; [d] ΔE_0, inner potential correction, fitted for each spectrum independently; [e] *R* factor. [f] This value indicates quantitative EXAFS analysis, least square fittings implemented in the ARTEMIS software were applied, based on the known structure of Cu or Ni.[1] Amplitude reduction factor (S_0^2) was fixed to 0.81 for Cu and 0.85 for Ni, according to the experimental EXAFS fit of Cu or Ni foil by fixing *CN* as the known crystallographic value. Data fitting range: 3.0 ≤ *k* (Å^{-1}) ≤ 12.0 and 1.5 ≤ *R* (Å) ≤ 3.2 (Cu foil); 3.0 ≤ *k* (Å^{-1}) ≤ 9.0 and 1.5 ≤ *R* (Å) ≤ ~3.5 (Cu *K*-edge of $CuNi_{0.05}$@OC). 3.0 ≤ *k* (/Å) ≤ 12.0 and 1.5 ≤ *R* (Å) ≤ 3.0 (Ni-foil); 3.0 ≤ *k* (/Å) ≤ 12.0 and 1.0 ≤ *R* (Å) ≤ ~2.2 (Ni *K*-edge of $CuNi_{0.05}$@OC). A reasonable range of EXAFS fitting parameters: 0.700 < S_0^2 < 1.000; *CN* > 0; σ^2 > 0 Å^2; |ΔE_0| < 10 eV; *R* factor < 0.02.

Table S5. Cu L_3VV Auger spectra fitting parameters for Cu/OC and $CuNi_{0.05}$@OC samples.

Sample	State		K.E. (eV)	FWHM (eV)	Peak shape [a]	Amount (%)
Cu/OC	Cu^0		918.8	2.11	GL	16.5
	$Cu^{\delta+}$	Cu^{2+}	917.4	2.72	GL	83.5
		Cu^{1+}	916.4	4.90	GL	
$CuNi_{0.05}$@OC	Cu^0		918.8	2.62	GL	63.3
	$Cu^{\delta+}$	Cu^{2+}	917.4	2.82	GL	36.7
		Cu^{1+}	916.4	6.26	GL	

[a] GL- Gaussian/Lorentzian line shape.

Table S6. Summary of CTH performance of *p*-CNB with hydrazine hydrate as hydrogen source over various catalysts.

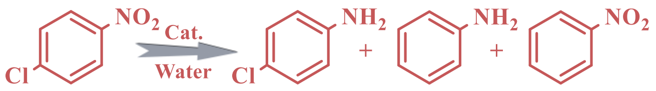

p-CNB *p*-CAN AN NB

Entry	Samples	Cat. mass (mg)	$N_2H_4 \cdot H_2O$ (mmol)	[*p*-CNB] (mmol)	T (°C)	t (h)	Solvent	TOF (h^{-1})	Conv. (%) [a]	Sel. (%) [a]	Refs.
1	$CuNi_{0.05}$@OC	20	1.5	0.5	50	4	water	45.2 [b]<br>0.21 [c]	97.9	99.4	This work
2	Ni SAs/NHCS	20	4	1	60	1.5	ethanol	29.9	100	99	[10]
3	Fe_1/N-C	20	1	0.2	60	2	ethanol	24.7	99	99	[11]
4	Fe_3O_4	10	6	1	80	1	ethanol	0.01	99	100	[12]
5	Ni/SiO_2	10	48	6	100	8	ethanol	43.2	97.8	94.5	[13]
6	Co@NC	10	8.2	1	80	1.5	ethanol	10.4	100	98	[14]
7	Co-Mo_2C/AC	94	6	1	25	2	ethanol	21.4	100	100	[15]
8	Co-Ag(1:1)@NC	5	2	0.5	80	1	ethanol	44.1	100	100	[16]
9	ZIF-8C@Pd@ZIF-8	10	1	0.1	85	1	acetonitrile	39.9	97.6	97.1	[17]

[a] The conv. of *p*-CNB and sel. toward *p*-CAN. [b] Ni TOF. [c] Cu TOF.

Table S7. Thermodynamic parameter values for the catalytic reduction of *p*-CNB using Cu/OC, Ni@OC, $CuNi_{0.05}$@OC, and $CuNi_{0.25}$@OC catalysts calculated using Arrhenius formula and Elling equation.

Samples	T (K)	ΔG (kJ mol^{-1})	Ea (kJ mol^{-1})	ΔH (kJ mol^{-1})	ΔS^{0}*(J mol^{-1} K^{-1})
Cu/OC	308	96.26	76.33	73.67	-73.34
	313	96.62			
	323	97.36			
	333	98.09			
Ni@OC	308	94.21	67.61	64.97	-96.50
	313	95.18			
	323	96.14			
	333	97.11			
$CuNi_{0.05}$@OC	298	87.24	51.39	48.82	-128.94
	308	88.53			
	313	89.18			
	323	90.47			
$CuNi_{0.25}$@OC	298	86.78	49.96	43.36	-132.28
	308	88.11			
	313	88.77			
	323	90.09			

Table S8. Recycling of Cu/OC and $CuNi_{0.05}$@OC catalysts in the catalytic reduction of *p*-CNB base on the TOF values [a].

Samples	Cycle	Time (min)	TOF (h^{-1}) [b]	Conv. (%) [c]	Sel. (%) [c]
Cu/OC	1	10	45.20 [b]	9.35	99.4
	2	10	44.22 [b]	9.15	98.6
	3	10	46.05 [b]	9.53	99.8
	4	10	44.65 [b]	9.24	99.7
	5	10	44.81[b]	9.27	98.9
	6	10	44.60 [b]	9.23	99.1
$CuNi_{0.05}$@OC	1	30	0.21[c]	5.27	99.3
	2	60	0.15 [c]	7.30	99.9
	3	90	0.065 [c]	4.91	99.4
	4	120	0.031 [c]	3.14	99.7
	5	240	0.022 [c]	4.35	99.2
	6	360	0.012 [c]	3.73	99.5

[a] Reaction conditions: hydrazine hydrate (1.5 mmol); substrate (0.5 mmol); catalyst (20 mg); water (15 mL); T =50 °C; 1000 rpm. [b] Ni TOF. [c] Cu TOF. [d] The conv. of *p*-CNB and sel. to *p*-CAN were determined by GC-MS.

Table S9. CTH of a rich variety of functionalized nitroaromatics over $CuNi_{0.05}$@OC [a].

Entry	Substrate	Product	t (h)	Conv. (%) [b]	Sel. (%) [b]	Entry	Substrate	Product	t (h)	Conv. (%) [b]	Sel. (%) [b]
**1**	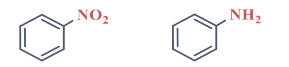		2	100	100	**17**	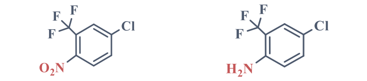		10	100	100
**2**	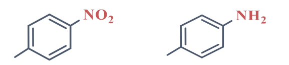		3	96.9	100	**18**	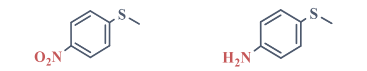		10	100	100
**3**	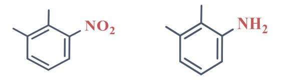		3	96.9	98.3	**19**	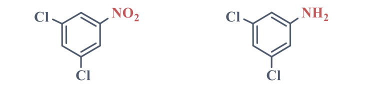		10	100	100
**4**	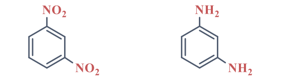		3	100	99.9	**20**	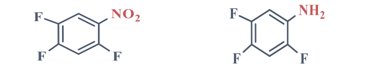		10	100	100
**5**	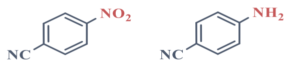		3	100	100	**21**	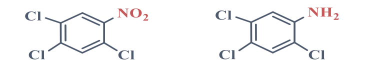		10	96.2	100
**6**	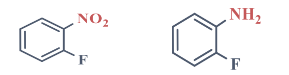		4	100	100	**22**	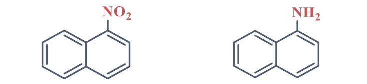		10	99.9	100
**7**	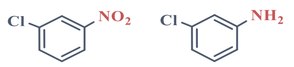		4	100	100	**23**	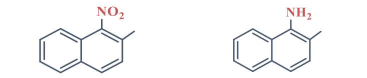		10	100	100
**8**	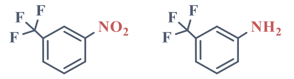		4	100	100	**24**	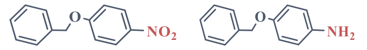		10	100	100
**9**	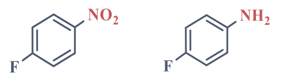		4	100	98.9	**25**	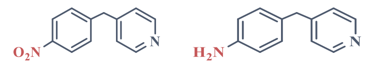		12	100	100
**10**	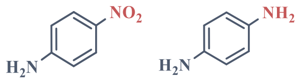		4	100	100	**26** [c]	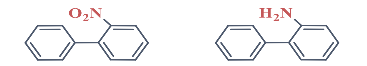		12	100	100
**11**	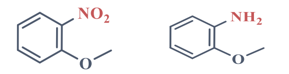		6	100	100	**27** [c]	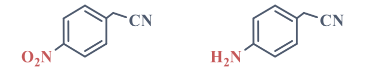		12	100	100
**12**	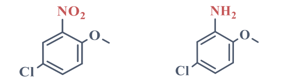		6	100	100	**28**	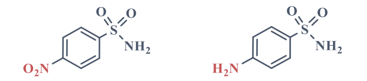		12	100	98.1
**13**	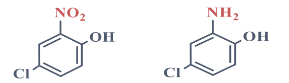		6	100	100	**29**	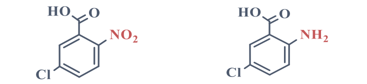		12	100	97.6
**14**	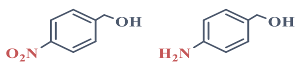		8	100	100	**30**	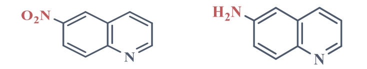		12	100	100
**15**	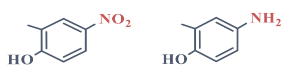		8	100	100	**31** [c]	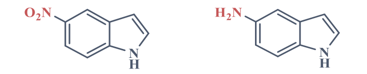		12	97.8	100
**16**	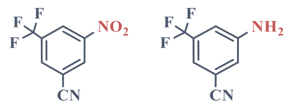		10	100	100						

[a] Reaction conditions: hydrazine hydrate (1.5 mmol); substrate (0.5 mmol); catalyst (20 mg); water (15 mL); T =50 °C; 1000 rpm. [b] The conv. of various aldehydes and sel. to the products were determined by GC-MS. [c] T =80 °C.

Table S10. Summary of CTH performance of *p*-NP with sodium borohydride as hydrogen source over various catalysts.

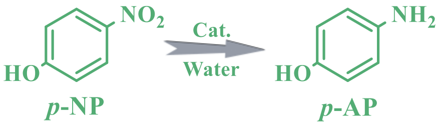

Entry	Samples	Cat. mass (mg)	[$NaBH_4$] (mM)	[*p*-NP] (mM)	T (°C)	t (min)	TOF (h^{-1})	K (min^{-1})	Refs.
1	$CuNi_{0.05}$@C	2	200	10	25	7	335.10	0.538	This work
2	ACOS-Ag	2.5	40	0.1	25	20	21.9	0.122	[18]
3	Cu/BNO	322.6	25	5	25	5	27	0.692	[19]
4	Foam C-HK/ML	0.1	40	0.2	25	1	163.404	1.75	[20]
5	Co_3O_4/HNTs	0.5	48	0.12	25	10	0.07	0.265	[21]
6	CoO_x/CN	2	200	0.4	25	10	7.73	0.252	[22]
7	APBAs/CF-200	20	200	0.01	25	3	29.82	2.057	[23]
8	$Ag_{0.5}$/C15h	0.2	112.5	0.09	25	2.5	354	0.51	[24]
9	Ag/ZrGP	10	500	0.1	25	10	1.07	0.175	[25]
10	Pd@COF-Ph	0.1	190	0.01	25	8.53	212.3	0.612	[26]
11	Pd NSs/CNNSs	0.7	1000	0.145	25	6	169.16	0.76	[27]
12	1.0 Pd/MMZ	2	200	5	25	14	10.5	0.321	[28]
13	Pd(1.71%)@FTRCC3	2	190	10	25	3	345.8	0.64	[29]
14	Pd/HAM@γ-AlOOH	0.2	100	10	25	4	33.4375	1.386	[30]
15	Ru/PC	0.09	125	0.08	25	5	321.67	1.29	[31]
16	Au/SBA-15-ZrO_2	99	10	10	25	20	13	0.14	[32]
17	Flat film@AuNPs	0.12	30	0.3	25	75	150	0.012	[33]
18	Au@g-C_3N_4	2	100	10	25	10	12	0.9	[34]
19	Fe_3O_4@COF-Au	3	990	1.8	25	20	354.6	0.222	[35]
20	P(AAm-co-MTM)@Au	30	100	10	30	25	323	0.136	[36]

Table S11. Selective hydrogenation of various biomass-derived aldehydes over the $CuNi_{0.05}$@OC [a].

Entry	Substrate	Product	t (h)	Conv. (%) [b]	Sel. (%) [b]
1	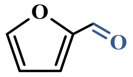	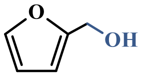	3	98.3<br>52.9 [c]	99.6<br>100 [c]
2	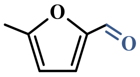	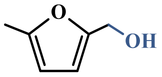	4	95.6	92.6
3	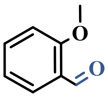	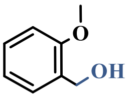	1.5	100	100
4	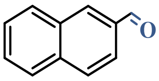	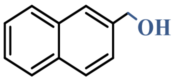	1.5	99.6	100
5	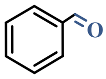	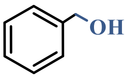	1	100	100
6	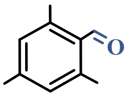	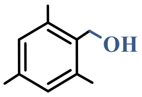	1	99.9	100
7	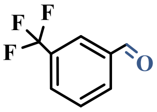	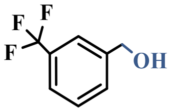	1	99.2	100

[a] Reaction conditions: $P(H_2)$ (1.0 MPa); T =50 °C; catalyst (20 mg); substrate (0.75 mmol); water (15 mL); 800 rpm. [b] The conv. of various aldehydes and sel. to the products were determined by GC-MS. [c] Cu/OC as catalyst.

Table S12. Summary of SH performance of FF with hydrogen as hydrogen source over various catalysts.

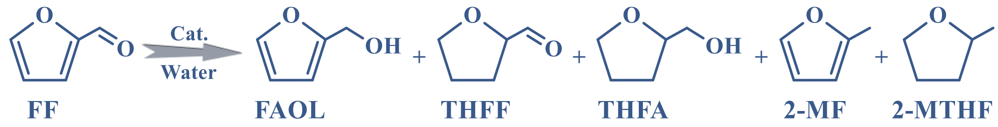

Entry	Samples	T (°C)	H_2 (MPa)	t (h)	Solvent	S/C	TOF (h^{-1})	Conv. (%) [a]	Sel. (%) [a]	Refs.
1	$CuNi_{0.05}$@OC	50	1	3	water	124.6	93.1	98.3	99.6	This work
2	Pt/HT	30	1.5	2	water	105.4	266.8	99.9	>99	[8]
3	Ru/ZP-A	90	1.3	7	water	25.7	2.3	62	97	[37]
4	Pt(3)Co(3)/C	35	1	10	water	911.1	91.1	100	100	[38]
5	$NiIn_{0.2}/MgO\text{-}Al_2O_3$	100	2	1	isopropanol	12	9.8	81.3	97.9	[39]
6	$Ni_{0.5}$@OMC-600	180	3	4	isopropanol	9.9	2.4	99	99	[40]
7	$Cu_{0.5}Ni_{2.5}AlO_y$	110	1.4	1.5	isopropanol	22.3	14	90	82	[41]
8	Na-Cu@TS-1	110	1	2	isopropanol	32.3	55.2	93	98.1	[42]
9	$NiBi/Al_2O_3$	100	2	2	isopropanol	58	29	100	97.7	[43]
10	Pt/m-CN-2	70	1	2	isopropanol	881.8	795	98.5	99.3	[44]
11	$Pt\text{-}CeO_2$@UIO-66-NH_2	80	1	30	isopropanol	214.1	7.1	100	99.4	[45]
12	Ni@OMC	180	3	12	isopropanol	26.4	2	92.3	98.6	[46]
13	5%Pd/C	120	6	3.5	isopropanol	513.8	189.7	82.4	28.2	[47]
14	Cu/NC_{300}@NMSSs	100	1	1	ethanol	13.1	11.5	100	>99	[48]
15	$Ni\text{-}Cu/TiO_2$	200	2.5	2	1,4-dioxane	40.7	18.5	91	35	[49]
16	nano-Co_2P/Al_2O_3	130	4	3	Methanol	3.7	1.2	100	98	[50]
17	2-Cu/C-400	170	2	3	n-propanol	18.1	5.99	99.9	99.8	[51]
18	Ni_3Fe_1/SiO_2	140	3.4	2	n-propanol	29.5	29.5	96.5	91.7	[52]
19	CuCo/Zn@NPC-600	140	2	4	Tetrahydrofuran	1515.2	344.7	>99.9	100	[53]

[a] The conv. of FF and sel. toward FAOL.

Table S13. Model structure information of CuNi@OC.

Fracttional coordinates for CuNi@OC in the DFT calculation											
Lattice parameters											
a	b	c	alpha	beta	gamma						
30.1209	30.1702	30.0273	90.0000	90.0000	90.0000						
Structure parameters											
NO.			x	y	z	***NO.***			x	y	z
**1**	C	C1	0.41984	0.6626	0.60642	**167**	C	C167	0.66166	0.36631	0.31903
**2**	C	C2	0.42169	0.69956	0.57328	**168**	C	C168	0.62902	0.37384	0.28299
**3**	C	C3	0.3895	0.70312	0.53522	**169**	C	C169	0.7215	0.40485	0.36388
**4**	C	C4	0.40269	0.72739	0.49335	**170**	C	C170	0.73554	0.45278	0.36465
**5**	C	C5	0.3777	0.71964	0.45231	**171**	C	C171	0.74113	0.47093	0.408
**6**	C	C6	0.34665	0.64053	0.57336	**172**	C	C172	0.71025	0.38065	0.40535
**7**	C	C7	0.35141	0.67209	0.53514	**173**	C	C173	0.69601	0.39899	0.32358
**8**	C	C8	0.32589	0.66458	0.49287	**174**	C	C174	0.71837	0.4758	0.32407
**9**	C	C9	0.33963	0.68782	0.45222	**175**	C	C175	0.69408	0.442	0.29995
**10**	C	C10	0.38144	0.63279	0.60742	**176**	C	C176	0.72502	0.55555	0.34888
**11**	C	C11	0.50339	0.67243	0.6212	**177**	C	C177	0.70793	0.59956	0.35158
**12**	C	C12	0.50306	0.70978	0.59218	**178**	C	C178	0.70996	0.62557	0.39824
**13**	C	C13	0.46252	0.72425	0.56983	**179**	C	C179	0.70975	0.52362	0.3168
**14**	C	C14	0.4608	0.64998	0.63088	**180**	C	C180	0.67484	0.61415	0.31628
**15**	C	C15	0.42382	0.57713	0.65895	**181**	C	C181	0.65593	0.58242	0.28289
**16**	C	C16	0.46127	0.61036	0.65981	**182**	C	C182	0.65632	0.68101	0.36369
**17**	C	C17	0.3841	0.58876	0.6324	**183**	C	C183	0.61694	0.70974	0.36569
**18**	C	C18	0.31258	0.60733	0.56943	**184**	C	C184	0.59752	0.72603	0.40663
**19**	C	C19	0.35148	0.55302	0.6222	**185**	C	C185	0.68009	0.66518	0.40319
**20**	C	C20	0.31491	0.56493	0.59321	**186**	C	C186	0.65334	0.65612	0.32229
**21**	C	C21	0.58659	0.66419	0.60436	**187**	C	C187	0.59205	0.70383	0.32434
**22**	C	C22	0.58382	0.70078	0.57084	**188**	C	C188	0.61405	0.66928	0.29826
**23**	C	C23	0.61845	0.70235	0.53278	**189**	C	C189	0.52263	0.73631	0.35973
**24**	C	C24	0.606	0.72243	0.49149	**190**	C	C190	0.47359	0.73819	0.36006
**25**	C	C25	0.62437	0.71506	0.4488	**191**	C	C191	0.44785	0.74367	0.40419
**26**	C	C26	0.54165	0.72562	0.56917	**192**	C	C192	0.54861	0.73908	0.40333
**27**	C	C27	0.54643	0.6514	0.63079	**193**	C	C193	0.54513	0.71475	0.3211
**28**	C	C28	0.33214	0.47156	0.60631	**194**	C	C194	0.44994	0.71843	0.32204
**29**	C	C29	0.29739	0.48546	0.57384	**195**	C	C195	0.4716	0.69352	0.28566
**30**	C	C30	0.28289	0.4576	0.53463	**196**	C	C196	0.54417	0.65689	0.25776
**31**	C	C31	0.26759	0.48024	0.49087	**197**	C	C197	0.52078	0.69212	0.2848
**32**	C	C32	0.27356	0.45379	0.45131	**198**	C	C198	0.59159	0.64159	0.26658
**33**	C	C33	0.2889	0.5324	0.56921	**199**	C	C199	0.61135	0.59522	0.25705
**34**	C	C34	0.35916	0.50509	0.63169	**200**	C	C200	0.65128	0.50126	0.25924
**35**	C	C35	0.39936	0.49231	0.65575	**201**	C	C201	0.67468	0.53605	0.28382

**36**	C	C36	0.47476	0.5113	0.68692	**202**	C	C202	0.6597	0.45269	0.26828
**37**	C	C37	0.43199	0.52841	0.67081	**203**	C	C203	0.62797	0.41632	0.25758
**38**	C	C38	0.50415	0.60344	0.68447	**204**	C	C204	0.55067	0.35779	0.25377
**39**	C	C39	0.54628	0.61229	0.65863	**205**	C	C205	0.59024	0.34481	0.28102
**40**	C	C40	0.62589	0.63451	0.60574	**206**	C	C206	0.50923	0.33721	0.26415
**41**	C	C41	0.66089	0.64086	0.57131	**207**	C	C207	0.37572	0.42496	0.25674
**42**	C	C42	0.65538	0.67106	0.5328	**208**	C	C208	0.37367	0.38045	0.28295
**43**	C	C43	0.67913	0.66304	0.48867	**209**	C	C209	0.34292	0.46085	0.26904
**44**	C	C44	0.66352	0.68394	0.44667	**210**	C	C210	0.34885	0.50955	0.26011
**45**	C	C45	0.45563	0.42876	0.67463	**211**	C	C211	0.37827	0.60386	0.26035
**46**	C	C46	0.48032	0.46392	0.69474	**212**	C	C212	0.33781	0.58963	0.28747
**47**	C	C47	0.41337	0.44405	0.65394	**213**	C	C213	0.39975	0.64747	0.27141
**48**	C	C48	0.38878	0.41013	0.62795	**214**	C	C214	0.44642	0.65837	0.2603
**49**	C	C49	0.34729	0.42454	0.60416	**215**	C	C215	0.51724	0.63709	0.22081
**50**	C	C50	0.3264	0.39438	0.57028	**216**	C	C216	0.58811	0.55622	0.23306
**51**	C	C51	0.29739	0.41032	0.53261	**217**	C	C217	0.5382	0.54226	0.21666
**52**	C	C52	0.29748	0.38492	0.48888	**218**	C	C218	0.58496	0.47663	0.21903
**53**	C	C53	0.28342	0.40679	0.44882	**219**	C	C219	0.61257	0.51269	0.23421
**54**	C	C54	0.62308	0.59095	0.63138	**220**	C	C220	0.58813	0.42928	0.23079
**55**	C	C55	0.58316	0.57911	0.6581	**221**	C	C221	0.54298	0.49522	0.20932
**56**	C	C56	0.57425	0.53077	0.66981	**222**	C	C222	0.5032	0.42314	0.21527
**57**	C	C57	0.53076	0.51304	0.6862	**223**	C	C223	0.54747	0.40168	0.22839
**58**	C	C58	0.52764	0.46623	0.69624	**224**	C	C224	0.45319	0.40712	0.21004
**59**	C	C59	0.6927	0.60654	0.56568	**225**	C	C225	0.50296	0.47007	0.20722
**60**	C	C60	0.67251	0.47326	0.60425	**226**	C	C226	0.42126	0.48857	0.2217
**61**	C	C61	0.70509	0.48555	0.56966	**227**	C	C227	0.41568	0.43992	0.23035
**62**	C	C62	0.71912	0.45634	0.53227	**228**	C	C228	0.3891	0.52295	0.23534
**63**	C	C63	0.7393	0.47625	0.49001	**229**	C	C229	0.46641	0.50179	0.20923
**64**	C	C64	0.74108	0.44675	0.44938	**230**	C	C230	0.45227	0.58351	0.22238
**65**	C	C65	0.71344	0.53143	0.5649	**231**	C	C231	0.40373	0.57107	0.23279
**66**	C	C66	0.68928	0.56461	0.58929	**232**	C	C232	0.46956	0.62811	0.23172
**67**	C	C67	0.65464	0.55439	0.62028	**233**	C	C233	0.48502	0.54725	0.21404
**68**	C	C68	0.40709	0.30291	0.56346	**234**	O	O234	0.50562	0.58788	0.7234
**69**	C	C69	0.45415	0.29667	0.56267	**235**	O	O235	0.794	0.55915	0.42855
**70**	C	C70	0.47316	0.27393	0.52445	**236**	O	O236	0.54083	0.79918	0.53021
**71**	C	C71	0.44838	0.26841	0.48249	**237**	O	O237	0.5533	0.31361	0.66161
**72**	C	C72	0.47386	0.26387	0.441	**238**	O	O238	0.20887	0.52567	0.39457
**73**	C	C73	0.48713	0.31961	0.59067	**239**	O	O239	0.5346	0.62574	0.18534
**74**	C	C74	0.46227	0.35703	0.62568	**240**	O	O240	0.4422	0.37606	0.18634
**75**	C	C75	0.41196	0.36623	0.61737	**241**	Cu	Cu241	0.56601	0.56817	0.49779
**76**	C	C76	0.38672	0.33749	0.58918	**242**	Cu	Cu242	0.56455	0.63843	0.54093
**77**	C	C77	0.34747	0.35241	0.56504	**243**	Cu	Cu243	0.6295	0.55951	0.54688
**78**	C	C78	0.62303	0.41316	0.63613	**244**	Cu	Cu244	0.62327	0.62328	0.48728
**79**	C	C79	0.65876	0.42694	0.60506	**245**	Cu	Cu245	0.46931	0.3839	0.27953

**80**	C	C80	0.64753	0.50729	0.63106	**246**	Cu	Cu246	0.5452	0.39144	0.31546
**81**	C	C81	0.60898	0.49504	0.65813	**247**	Cu	Cu247	0.36248	0.46871	0.33773
**82**	C	C82	0.5993	0.4468	0.66313	**248**	Cu	Cu248	0.42705	0.44714	0.29652
**83**	C	C83	0.55543	0.43245	0.68133	**249**	Cu	Cu249	0.43255	0.50982	0.34161
**84**	C	C84	0.56208	0.35343	0.65752	**250**	Cu	Cu250	0.49379	0.45404	0.33009
**85**	C	C85	0.53205	0.39061	0.66828	**251**	Cu	Cu251	0.49504	0.51481	0.27788
**86**	C	C86	0.48248	0.38841	0.65908	**252**	Cu	Cu252	0.55464	0.4625	0.2824
**87**	C	C87	0.6018	0.36942	0.63111	**253**	Cu	Cu253	0.55942	0.51439	0.34185
**88**	C	C88	0.61276	0.34286	0.59243	**254**	Cu	Cu254	0.61956	0.45811	0.32581
**89**	C	C89	0.53399	0.30394	0.56675	**255**	Cu	Cu255	0.37158	0.56279	0.34362
**90**	C	C90	0.52089	0.27752	0.52653	**256**	Cu	Cu256	0.43244	0.5628	0.28955
**91**	C	C91	0.54765	0.26819	0.48547	**257**	Cu	Cu257	0.43372	0.62521	0.33436
**92**	C	C92	0.52404	0.26245	0.44241	**258**	Cu	Cu258	0.49171	0.5655	0.33798
**93**	C	C93	0.58372	0.31289	0.56675	**259**	Cu	Cu259	0.55558	0.57111	0.29027
**94**	C	C94	0.67499	0.39547	0.57043	**260**	Cu	Cu260	0.51794	0.63437	0.31808
**95**	C	C95	0.70396	0.4093	0.53262	**261**	Cu	Cu261	0.62331	0.56683	0.34079
**96**	C	C96	0.7036	0.38234	0.48965	**262**	Cu	Cu262	0.36298	0.40816	0.39584
**97**	C	C97	0.72292	0.40006	0.44911	**263**	Cu	Cu263	0.42445	0.3766	0.35258
**98**	C	C98	0.65165	0.35419	0.56641	**264**	Cu	Cu264	0.43698	0.38844	0.43556
**99**	C	C99	0.47424	0.75062	0.53134	**265**	Ni	Ni265	0.48886	0.32705	0.42245
**100**	C	C100	0.52359	0.76334	0.53886	**266**	Cu	Cu266	0.49468	0.3919	0.37719
**101**	C	C101	0.44908	0.74723	0.49071	**267**	Cu	Cu267	0.55155	0.33782	0.37399
**102**	C	C102	0.47296	0.74897	0.44557	**268**	Cu	Cu268	0.55436	0.39024	0.42993
**103**	C	C103	0.5209	0.74063	0.44333	**269**	Cu	Cu269	0.61394	0.38956	0.37496
**104**	C	C104	0.27283	0.55417	0.52803	**270**	Cu	Cu270	0.29992	0.48083	0.40019
**105**	C	C105	0.28704	0.60084	0.52892	**271**	Ni	Ni271	0.32592	0.50385	0.4681
**106**	C	C106	0.26399	0.53081	0.48665	**272**	Cu	Cu272	0.38613	0.45	0.45539
**107**	C	C107	0.2623	0.56087	0.44483	**273**	Cu	Cu273	0.37773	0.50547	0.40215
**108**	C	C108	0.29395	0.62744	0.4895	**274**	Cu	Cu274	0.43193	0.44772	0.38534
**109**	C	C109	0.27967	0.6075	0.44717	**275**	Cu	Cu275	0.44382	0.50714	0.44598
**110**	C	C110	0.38266	0.2975	0.52231	**276**	Cu	Cu276	0.49841	0.44856	0.43462
**111**	C	C111	0.34627	0.32857	0.52309	**277**	Cu	Cu277	0.49526	0.50848	0.38771
**112**	C	C112	0.40138	0.28293	0.48059	**278**	Cu	Cu278	0.55814	0.44538	0.37458
**113**	C	C113	0.37948	0.29723	0.43884	**279**	Cu	Cu279	0.55821	0.50571	0.43244
**114**	C	C114	0.32508	0.34492	0.48373	**280**	Cu	Cu280	0.61358	0.44354	0.43289
**115**	C	C115	0.34102	0.32766	0.4404	**281**	Cu	Cu281	0.62557	0.50567	0.389
**116**	C	C116	0.65026	0.32958	0.52552	**282**	Cu	Cu282	0.67152	0.44486	0.38594
**117**	C	C117	0.60991	0.30325	0.52535	**283**	Ni	Ni283	0.69319	0.49554	0.44334
**118**	C	C118	0.67318	0.34351	0.4857	**284**	Cu	Cu284	0.32295	0.55892	0.4092
**119**	C	C119	0.65836	0.32461	0.44228	**285**	Cu	Cu285	0.38412	0.56148	0.45866
**120**	C	C120	0.59435	0.28408	0.48388	**286**	Cu	Cu286	0.37249	0.62128	0.407
**121**	C	C121	0.61922	0.29444	0.44153	**287**	Cu	Cu287	0.43255	0.56907	0.39395
**122**	C	C122	0.71831	0.59906	0.52524	**288**	Cu	Cu288	0.43867	0.62173	0.45172
**123**	C	C123	0.73105	0.55201	0.52521	**289**	Cu	Cu289	0.50355	0.56564	0.44262

**124**	C	C124	0.71088	0.62476	0.48468	**290**	Cu	Cu290	0.49029	0.62468	0.39146
**125**	C	C125	0.72633	0.60516	0.44171	**291**	Cu	Cu291	0.55658	0.58555	0.37775
**126**	C	C126	0.74268	0.52733	0.48573	**292**	Cu	Cu292	0.55701	0.63145	0.44674
**127**	C	C127	0.75911	0.56252	0.44846	**293**	Cu	Cu293	0.60953	0.5659	0.43327
**128**	C	C128	0.3783	0.7168	0.36903	**294**	Cu	Cu294	0.62123	0.63043	0.39631
**129**	C	C129	0.33996	0.68748	0.36885	**295**	Cu	Cu295	0.68301	0.55896	0.40082
**130**	C	C130	0.32128	0.67054	0.40947	**296**	Cu	Cu296	0.43014	0.67418	0.39424
**131**	C	C131	0.3989	0.73287	0.40919	**297**	Ni	Ni297	0.49173	0.68328	0.44282
**132**	C	C132	0.40289	0.70961	0.32784	**298**	Cu	Cu298	0.55214	0.67113	0.38115
**133**	C	C133	0.34009	0.66298	0.32774	**299**	Cu	Cu299	0.39252	0.3862	0.5035
**134**	C	C134	0.37891	0.67594	0.30309	**300**	Cu	Cu300	0.45263	0.3365	0.49334
**135**	C	C135	0.25975	0.45378	0.36047	**301**	Cu	Cu301	0.47168	0.3806	0.56002
**136**	C	C136	0.28049	0.41119	0.36314	**302**	Cu	Cu302	0.50243	0.39367	0.48819
**137**	C	C137	0.29336	0.38699	0.40386	**303**	Cu	Cu303	0.54509	0.33341	0.47995
**138**	C	C138	0.28038	0.48018	0.32527	**304**	Cu	Cu304	0.55616	0.376	0.54665
**139**	C	C139	0.30643	0.40528	0.32275	**305**	Cu	Cu305	0.61211	0.37949	0.48381
**140**	C	C140	0.30808	0.44838	0.29992	**306**	Cu	Cu306	0.34963	0.45188	0.52453
**141**	C	C141	0.34871	0.34161	0.35596	**307**	Cu	Cu307	0.41189	0.43812	0.56922
**142**	C	C142	0.38839	0.31242	0.35605	**308**	Cu	Cu308	0.40023	0.50506	0.51373
**143**	C	C143	0.40357	0.29054	0.39754	**309**	Cu	Cu309	0.44928	0.44464	0.50264
**144**	C	C144	0.32524	0.34968	0.39954	**310**	Cu	Cu310	0.46147	0.50121	0.5632
**145**	C	C145	0.33996	0.37238	0.3176	**311**	Cu	Cu311	0.51703	0.44698	0.54921
**146**	C	C146	0.41912	0.31589	0.3171	**312**	Cu	Cu312	0.50741	0.50389	0.49328
**147**	C	C147	0.41269	0.35341	0.28678	**313**	Cu	Cu313	0.56521	0.44449	0.49056
**148**	C	C148	0.29257	0.60433	0.36039	**314**	Cu	Cu314	0.57021	0.50635	0.54691
**149**	C	C149	0.27294	0.56034	0.35736	**315**	Cu	Cu315	0.61087	0.44106	0.55481
**150**	C	C150	0.24318	0.54701	0.39825	**316**	Cu	Cu316	0.61951	0.50645	0.48538
**151**	C	C151	0.29438	0.62926	0.40531	**317**	Cu	Cu317	0.66034	0.44247	0.49379
**152**	C	C152	0.32077	0.61991	0.32273	**318**	Cu	Cu318	0.34318	0.55509	0.52533
**153**	C	C153	0.28685	0.5289	0.32012	**319**	Cu	Cu319	0.40718	0.55898	0.57304
**154**	C	C154	0.32212	0.54247	0.28729	**320**	Cu	Cu320	0.39084	0.61892	0.51437
**155**	C	C155	0.47609	0.27217	0.35921	**321**	Cu	Cu321	0.44899	0.56471	0.50532
**156**	C	C156	0.52474	0.26824	0.36013	**322**	Cu	Cu322	0.46287	0.62106	0.56683
**157**	C	C157	0.55035	0.26717	0.40089	**323**	Cu	Cu323	0.50981	0.56096	0.5493
**158**	C	C158	0.44959	0.27269	0.39911	**324**	Cu	Cu324	0.50311	0.62412	0.49876
**159**	C	C159	0.46234	0.29255	0.31872	**325**	Cu	Cu325	0.67404	0.56268	0.48031
**160**	C	C160	0.54147	0.28675	0.31892	**326**	Cu	Cu326	0.44881	0.67594	0.50902
**161**	C	C161	0.50314	0.30162	0.29324	**327**	Cu	Cu327	0.54453	0.70665	0.4929
**162**	C	C162	0.6138	0.30658	0.3568	**328**	Cu	Cu328	0.47716	0.44407	0.61353
**163**	C	C163	0.6534	0.33583	0.35786	**329**	Cu	Cu329	0.53252	0.5032	0.61407
**164**	C	C164	0.67631	0.34457	0.4015	**330**	Cu	Cu330	0.55651	0.42344	0.61026
**165**	C	C165	0.59712	0.28468	0.39928	**331**	Cu	Cu331	0.47523	0.55632	0.61844
**166**	C	C166	0.58425	0.30913	0.31603	**332**	Cu	Cu332	0.56626	0.57512	0.59343

Table S14. Model structure information of Ni@OC.

Fracttional coordinates for Ni@OC in the DFT calculation											
Lattice parameters											
a	b	c	alpha	beta	gamma						
30.1209	30.1702	30.0273	90.0000	90.0000	90.0000						
Structure parameters											
NO.			x	y	z	***NO.***			x	y	z
**1**	C	C1	0.42567	0.66426	0.59954	**167**	C	C167	0.65651	0.36576	0.32191
**2**	C	C2	0.42799	0.70034	0.56584	**168**	C	C168	0.62498	0.37221	0.28517
**3**	C	C3	0.3965	0.70334	0.52761	**169**	C	C169	0.71701	0.40515	0.36576
**4**	C	C4	0.40963	0.72711	0.48611	**170**	C	C170	0.73179	0.45195	0.36508
**5**	C	C5	0.38479	0.71923	0.44591	**171**	C	C171	0.73777	0.47213	0.40667
**6**	C	C6	0.35282	0.64319	0.56604	**172**	C	C172	0.70842	0.37988	0.40603
**7**	C	C7	0.35719	0.67453	0.52833	**173**	C	C173	0.69088	0.39862	0.326
**8**	C	C8	0.33097	0.66727	0.48731	**174**	C	C174	0.71488	0.47364	0.32363
**9**	C	C9	0.3464	0.68824	0.44633	**175**	C	C175	0.69	0.43999	0.30056
**10**	C	C10	0.38664	0.63552	0.60055	**176**	C	C176	0.72147	0.55151	0.34658
**11**	C	C11	0.50861	0.67432	0.61512	**177**	C	C177	0.70627	0.59598	0.34753
**12**	C	C12	0.50899	0.71189	0.5875	**178**	C	C178	0.70725	0.62001	0.39339
**13**	C	C13	0.46898	0.72486	0.56304	**179**	C	C179	0.70775	0.52033	0.31388
**14**	C	C14	0.46599	0.65194	0.62446	**180**	C	C180	0.67344	0.60899	0.31171
**15**	C	C15	0.4269	0.58206	0.65463	**181**	C	C181	0.65594	0.57805	0.27732
**16**	C	C16	0.46596	0.61279	0.65366	**182**	C	C182	0.65598	0.67527	0.35875
**17**	C	C17	0.38816	0.5929	0.6259	**183**	C	C183	0.6192	0.70653	0.35945
**18**	C	C18	0.31833	0.6101	0.56234	**184**	C	C184	0.60098	0.724	0.40003
**19**	C	C19	0.35605	0.5571	0.61602	**185**	C	C185	0.67824	0.66026	0.39842
**20**	C	C20	0.32046	0.5682	0.58611	**186**	C	C186	0.65342	0.65135	0.31703
**21**	C	C21	0.5912	0.6651	0.59824	**187**	C	C187	0.59472	0.70048	0.31807
**22**	C	C22	0.58885	0.70204	0.56465	**188**	C	C188	0.61591	0.66579	0.29204
**23**	C	C23	0.62143	0.70245	0.52621	**189**	C	C189	0.52668	0.73236	0.35437
**24**	C	C24	0.60659	0.71904	0.48395	**190**	C	C190	0.47774	0.73309	0.35587
**25**	C	C25	0.62629	0.71286	0.44169	**191**	C	C191	0.4531	0.74313	0.39804
**26**	C	C26	0.54753	0.72671	0.56309	**192**	C	C192	0.55279	0.73886	0.39685
**27**	C	C27	0.55115	0.65166	0.62298	**193**	C	C193	0.54793	0.70967	0.31629
**28**	C	C28	0.3354	0.47601	0.60118	**194**	C	C194	0.45291	0.7128	0.31814
**29**	C	C29	0.3018	0.48972	0.56624	**195**	C	C195	0.47449	0.68704	0.28298
**30**	C	C30	0.28973	0.4623	0.52611	**196**	C	C196	0.54566	0.65099	0.2539
**31**	C	C31	0.27453	0.48439	0.4798	**197**	C	C197	0.52344	0.68477	0.28232
**32**	C	C32	0.27407	0.45428	0.43762	**198**	C	C198	0.5918	0.63643	0.26241
**33**	C	C33	0.29445	0.53563	0.56198	**199**	C	C199	0.61121	0.58983	0.25248
**34**	C	C34	0.36277	0.50951	0.62688	**200**	C	C200	0.651	0.4973	0.25465
**35**	C	C35	0.40087	0.49729	0.65457	**201**	C	C201	0.67391	0.5321	0.27946

**36**	C	C36	0.47866	0.51801	0.67884	**202**	C	C202	0.65655	0.4501	0.26796
**37**	C	C37	0.43366	0.53431	0.6672	**203**	C	C203	0.62482	0.41381	0.25834
**38**	C	C38	0.50868	0.60707	0.68018	**204**	C	C204	0.5467	0.3573	0.25775
**39**	C	C39	0.5511	0.61295	0.65276	**205**	C	C205	0.58593	0.3441	0.28455
**40**	C	C40	0.6291	0.63449	0.59881	**206**	C	C206	0.5043	0.34055	0.27253
**41**	C	C41	0.6636	0.64026	0.56429	**207**	C	C207	0.37716	0.42357	0.26352
**42**	C	C42	0.65702	0.66929	0.52548	**208**	C	C208	0.37526	0.37946	0.28894
**43**	C	C43	0.67731	0.65845	0.4816	**209**	C	C209	0.34493	0.45987	0.2766
**44**	C	C44	0.66371	0.68105	0.44043	**210**	C	C210	0.35204	0.50745	0.26565
**45**	C	C45	0.45808	0.43418	0.67007	**211**	C	C211	0.38211	0.60033	0.26131
**46**	C	C46	0.48442	0.47061	0.6853	**212**	C	C212	0.3419	0.5878	0.2886
**47**	C	C47	0.4143	0.44896	0.6538	**213**	C	C213	0.40229	0.64496	0.26885
**48**	C	C48	0.39092	0.41551	0.62637	**214**	C	C214	0.44885	0.65366	0.25755
**49**	C	C49	0.34914	0.42894	0.60214	**215**	C	C215	0.51805	0.63147	0.21705
**50**	C	C50	0.32895	0.39898	0.56895	**216**	C	C216	0.58924	0.55151	0.22707
**51**	C	C51	0.30205	0.41489	0.52995	**217**	C	C217	0.53857	0.53775	0.21572
**52**	C	C52	0.30117	0.3882	0.48867	**218**	C	C218	0.58545	0.47195	0.21469
**53**	C	C53	0.28545	0.40691	0.44745	**219**	C	C219	0.61391	0.50806	0.2275
**54**	C	C54	0.62786	0.59262	0.62629	**220**	C	C220	0.58698	0.42549	0.22923
**55**	C	C55	0.59031	0.58339	0.65678	**221**	C	C221	0.54304	0.4904	0.20729
**56**	C	C56	0.58182	0.53567	0.66891	**222**	C	C222	0.50136	0.41879	0.21574
**57**	C	C57	0.53646	0.51897	0.68131	**223**	C	C223	0.54551	0.39802	0.2291
**58**	C	C58	0.5311	0.47218	0.6889	**224**	C	C224	0.45084	0.40187	0.21346
**59**	C	C59	0.69653	0.60709	0.56105	**225**	C	C225	0.50256	0.46564	0.20686
**60**	C	C60	0.67746	0.4753	0.60377	**226**	C	C226	0.42197	0.48422	0.22597
**61**	C	C61	0.70933	0.48677	0.56884	**227**	C	C227	0.41528	0.43602	0.2339
**62**	C	C62	0.72081	0.45695	0.53086	**228**	C	C228	0.39057	0.51954	0.23869
**63**	C	C63	0.73618	0.47619	0.48742	**229**	C	C229	0.46664	0.49749	0.21148
**64**	C	C64	0.73711	0.44729	0.44728	**230**	C	C230	0.45358	0.57873	0.22276
**65**	C	C65	0.71782	0.53206	0.56257	**231**	C	C231	0.40629	0.56634	0.23519
**66**	C	C66	0.69386	0.56612	0.58588	**232**	C	C232	0.47094	0.62315	0.22849
**67**	C	C67	0.65978	0.55647	0.61731	**233**	C	C233	0.48586	0.54258	0.21442
**68**	C	C68	0.40767	0.30662	0.56599	**234**	O	O234	0.50929	0.5966	0.7197
**69**	C	C69	0.45464	0.30017	0.56532	**235**	O	O235	0.79175	0.55518	0.42396
**70**	C	C70	0.47378	0.27794	0.52671	**236**	O	O236	0.54954	0.79383	0.51521
**71**	C	C71	0.44888	0.26961	0.48568	**237**	O	O237	0.55653	0.31897	0.66602
**72**	C	C72	0.47368	0.26432	0.44404	**238**	O	O238	0.2161	0.52474	0.39373
**73**	C	C73	0.48719	0.32293	0.59341	**239**	O	O239	0.53491	0.62173	0.18073
**74**	C	C74	0.46381	0.36162	0.62397	**240**	O	O240	0.43949	0.37336	0.18617
**75**	C	C75	0.41303	0.37077	0.61779	**241**	Ni	Ni241	0.56788	0.56095	0.49953
**76**	C	C76	0.38736	0.34192	0.59076	**242**	Ni	Ni242	0.56487	0.61733	0.55979
**77**	C	C77	0.34866	0.35669	0.56598	**243**	Ni	Ni243	0.62356	0.55739	0.55756
**78**	C	C78	0.62613	0.417	0.63504	**244**	Ni	Ni244	0.62153	0.61839	0.49796
**79**	C	C79	0.66196	0.42961	0.60433	**245**	Ni	Ni245	0.46485	0.38699	0.27927

**80**	C	C80	0.65318	0.50955	0.63002	**246**	Ni	Ni246	0.54205	0.38919	0.31754
**81**	C	C81	0.61545	0.49859	0.65805	**247**	Ni	Ni247	0.36312	0.4802	0.34048
**82**	C	C82	0.60353	0.45098	0.66152	**248**	Ni	Ni248	0.42603	0.45514	0.29513
**83**	C	C83	0.55872	0.43711	0.6761	**249**	Ni	Ni249	0.43827	0.5121	0.34582
**84**	C	C84	0.5639	0.3583	0.65778	**250**	Ni	Ni250	0.49309	0.45396	0.33228
**85**	C	C85	0.53381	0.39622	0.66394	**251**	Ni	Ni251	0.48888	0.51448	0.27611
**86**	C	C86	0.48478	0.39437	0.65596	**252**	Ni	Ni252	0.53648	0.45137	0.26791
**87**	C	C87	0.60309	0.37404	0.63068	**253**	Ni	Ni253	0.55522	0.50646	0.32858
**88**	C	C88	0.61349	0.34645	0.59313	**254**	Ni	Ni254	0.60398	0.44693	0.31416
**89**	C	C89	0.53469	0.30868	0.56843	**255**	Ni	Ni255	0.37639	0.56925	0.34368
**90**	C	C90	0.52137	0.28149	0.52846	**256**	Ni	Ni256	0.43447	0.56841	0.29286
**91**	C	C91	0.5473	0.27072	0.48778	**257**	Ni	Ni257	0.45635	0.6357	0.32669
**92**	C	C92	0.52311	0.2645	0.44517	**258**	Ni	Ni258	0.49771	0.56491	0.33616
**93**	C	C93	0.58361	0.31641	0.5685	**259**	Ni	Ni259	0.5555	0.5638	0.28075
**94**	C	C94	0.67583	0.3977	0.56996	**260**	Ni	Ni260	0.53761	0.62934	0.31828
**95**	C	C95	0.70499	0.41017	0.53184	**261**	Ni	Ni261	0.62177	0.56053	0.32977
**96**	C	C96	0.70303	0.38305	0.48947	**262**	Ni	Ni262	0.37995	0.38537	0.40325
**97**	C	C97	0.72151	0.40016	0.44801	**263**	Ni	Ni263	0.44251	0.34159	0.37896
**98**	C	C98	0.65209	0.35652	0.56679	**264**	Ni	Ni264	0.44816	0.39102	0.44006
**99**	C	C99	0.48183	0.7485	0.52361	**265**	Ni	Ni265	0.49885	0.3292	0.43408
**100**	C	C100	0.53085	0.7611	0.53032	**266**	Ni	Ni266	0.49695	0.39413	0.38054
**101**	C	C101	0.45599	0.74489	0.48311	**267**	Ni	Ni267	0.55244	0.33553	0.3764
**102**	C	C102	0.47938	0.74795	0.43888	**268**	Ni	Ni268	0.55649	0.39148	0.43238
**103**	C	C103	0.52698	0.74437	0.43638	**269**	Ni	Ni269	0.6136	0.38934	0.37672
**104**	C	C104	0.27864	0.55723	0.52111	**270**	Ni	Ni270	0.33475	0.45101	0.41243
**105**	C	C105	0.29157	0.60422	0.52265	**271**	Ni	Ni271	0.33094	0.51401	0.46048
**106**	C	C106	0.26891	0.53418	0.4798	**272**	Ni	Ni272	0.40319	0.45231	0.44669
**107**	C	C107	0.2676	0.56473	0.44048	**273**	Ni	Ni273	0.38586	0.51492	0.40341
**108**	C	C108	0.29735	0.63211	0.4845	**274**	Ni	Ni274	0.42924	0.43952	0.37548
**109**	C	C109	0.28327	0.61175	0.44285	**275**	Ni	Ni275	0.45106	0.51308	0.44306
**110**	C	C110	0.38358	0.30014	0.52505	**276**	Ni	Ni276	0.49906	0.4505	0.43762
**111**	C	C111	0.34753	0.33131	0.52509	**277**	Ni	Ni277	0.50347	0.50885	0.38732
**112**	C	C112	0.40194	0.28301	0.4839	**278**	Ni	Ni278	0.5578	0.44648	0.37805
**113**	C	C113	0.37909	0.29329	0.44221	**279**	Ni	Ni279	0.5612	0.50355	0.43856
**114**	C	C114	0.32658	0.34614	0.48526	**280**	Ni	Ni280	0.61658	0.44656	0.43246
**115**	C	C115	0.34242	0.32568	0.44318	**281**	Ni	Ni281	0.61526	0.50552	0.38174
**116**	C	C116	0.64928	0.33215	0.52603	**282**	Ni	Ni282	0.66446	0.44986	0.36601
**117**	C	C117	0.60908	0.30584	0.52605	**283**	Ni	Ni283	0.68537	0.49536	0.43442
**118**	C	C118	0.67262	0.34408	0.48615	**284**	Ni	Ni284	0.32976	0.5725	0.40759
**119**	C	C119	0.6563	0.32517	0.44388	**285**	Ni	Ni285	0.39235	0.5714	0.45565
**120**	C	C120	0.5945	0.28377	0.48573	**286**	Ni	Ni286	0.38395	0.63454	0.40896
**121**	C	C121	0.61873	0.29341	0.44335	**287**	Ni	Ni287	0.43888	0.57873	0.3894
**122**	C	C122	0.71975	0.59791	0.51972	**288**	Ni	Ni288	0.45166	0.62524	0.45186
**123**	C	C123	0.73279	0.55121	0.52108	**289**	Ni	Ni289	0.50882	0.56471	0.44346

**124**	C	C124	0.70896	0.62037	0.47824	**290**	Ni	Ni290	0.5036	0.62267	0.39118
**125**	C	C125	0.72359	0.59926	0.43576	**291**	Ni	Ni291	0.56192	0.56977	0.37932
**126**	C	C126	0.74004	0.52621	0.48136	**292**	Ni	Ni292	0.56506	0.62219	0.44321
**127**	C	C127	0.75662	0.55866	0.44265	**293**	Ni	Ni293	0.61481	0.56054	0.43791
**128**	C	C128	0.3832	0.71696	0.36257	**294**	Ni	Ni294	0.62251	0.62061	0.38685
**129**	C	C129	0.3451	0.68819	0.36376	**295**	Ni	Ni295	0.67869	0.55838	0.39475
**130**	C	C130	0.32651	0.67233	0.4044	**296**	Ni	Ni296	0.44905	0.67773	0.39384
**131**	C	C131	0.40573	0.73071	0.40257	**297**	Ni	Ni297	0.51227	0.68414	0.44719
**132**	C	C132	0.40532	0.70993	0.32092	**298**	Ni	Ni298	0.56107	0.67284	0.38161
**133**	C	C133	0.3433	0.66356	0.32281	**299**	Ni	Ni299	0.38535	0.38118	0.48609
**134**	C	C134	0.38085	0.67612	0.29693	**300**	Ni	Ni300	0.44906	0.33568	0.49624
**135**	C	C135	0.27682	0.45643	0.38313	**301**	Ni	Ni301	0.47428	0.37647	0.55951
**136**	C	C136	0.28641	0.41044	0.37014	**302**	Ni	Ni302	0.50524	0.39291	0.49089
**137**	C	C137	0.29575	0.38294	0.40702	**303**	Ni	Ni303	0.55392	0.33564	0.48789
**138**	C	C138	0.29127	0.482	0.3403	**304**	Ni	Ni304	0.55733	0.37661	0.55178
**139**	C	C139	0.30752	0.40473	0.32843	**305**	Ni	Ni305	0.61689	0.38258	0.48325
**140**	C	C140	0.31127	0.44883	0.30965	**306**	Ni	Ni306	0.3499	0.44892	0.50068
**141**	C	C141	0.34964	0.34024	0.35963	**307**	Ni	Ni307	0.39193	0.46502	0.56605
**142**	C	C142	0.38788	0.30948	0.35869	**308**	Ni	Ni308	0.40115	0.50816	0.50341
**143**	C	C143	0.40274	0.28606	0.40047	**309**	Ni	Ni309	0.44244	0.43525	0.51242
**144**	C	C144	0.32581	0.34553	0.40227	**310**	Ni	Ni310	0.46083	0.50181	0.55702
**145**	C	C145	0.34098	0.37148	0.32218	**311**	Ni	Ni311	0.51363	0.44396	0.55098
**146**	C	C146	0.41905	0.31488	0.32081	**312**	Ni	Ni312	0.50838	0.50189	0.49595
**147**	C	C147	0.41452	0.35356	0.29209	**313**	Ni	Ni313	0.56582	0.44367	0.49307
**148**	C	C148	0.29715	0.60662	0.3589	**314**	Ni	Ni314	0.56647	0.49987	0.55531
**149**	C	C149	0.27862	0.56209	0.35898	**315**	Ni	Ni315	0.61867	0.43994	0.55087
**150**	C	C150	0.24845	0.54934	0.39683	**316**	Ni	Ni316	0.62079	0.50323	0.49478
**151**	C	C151	0.29775	0.63274	0.40129	**317**	Ni	Ni317	0.6673	0.44224	0.4901
**152**	C	C152	0.32506	0.62011	0.3211	**318**	Ni	Ni318	0.34648	0.56312	0.52086
**153**	C	C153	0.29493	0.52873	0.328	**319**	Ni	Ni319	0.40734	0.55928	0.56909
**154**	C	C154	0.32765	0.54119	0.29226	**320**	Ni	Ni320	0.39969	0.62497	0.51359
**155**	C	C155	0.47489	0.26646	0.3619	**321**	Ni	Ni321	0.4533	0.56577	0.50415
**156**	C	C156	0.52281	0.26513	0.36263	**322**	Ni	Ni322	0.46589	0.62048	0.56407
**157**	C	C157	0.54865	0.26792	0.40329	**323**	Ni	Ni323	0.51248	0.5583	0.55099
**158**	C	C158	0.44894	0.26881	0.40206	**324**	Ni	Ni324	0.51259	0.61967	0.50028
**159**	C	C159	0.46105	0.29088	0.32276	**325**	Ni	Ni325	0.6752	0.56057	0.48448
**160**	C	C160	0.5386	0.28619	0.32225	**326**	Ni	Ni326	0.46283	0.68101	0.50923
**161**	C	C161	0.5001	0.30138	0.29748	**327**	Ni	Ni327	0.56127	0.68027	0.50559
**162**	C	C162	0.61093	0.30601	0.35959	**328**	Ni	Ni328	0.45723	0.43807	0.60308
**163**	C	C163	0.64982	0.3358	0.36038	**329**	Ni	Ni329	0.51333	0.491	0.62366
**164**	C	C164	0.67331	0.34538	0.40284	**330**	Ni	Ni330	0.56468	0.42972	0.60828
**165**	C	C165	0.5956	0.28412	0.40173	**331**	Ni	Ni331	0.47144	0.55718	0.61503
**166**	C	C166	0.58087	0.30877	0.3197	**332**	Ni	Ni332	0.55631	0.55669	0.61042

Table S15. Model structure information of Cu/OC.

Fracttional coordinates for Cu/OC in the DFT calculation											
Lattice parameters											
a	b	c	alpha	beta	gamma						
14.7599	14.7599	15.0000	90.0000	90.0000	90.0000						
Structure parameters											
NO.			x	y	z	***NO.***			x	y	z
**1**	C	C001	0.00919	0.99987	0.18601	**62**	C	C062	0.2849	0.88398	0.21503
**2**	C	C002	0.12022	0.05556	0.19267	**63**	C	C063	0.33773	0.82481	0.22571
**3**	C	C003	0.17548	0.99927	0.19877	**64**	C	C064	0.44962	0.87052	0.21686
**4**	C	C004	0.2864	0.05431	0.20663	**65**	C	C065	0.5099	0.81215	0.2068
**5**	C	C005	0.34087	0.9964	0.21304	**66**	C	C066	0.6183	0.8836	0.19431
**6**	C	C006	0.45195	0.04941	0.21702	**67**	C	C067	0.67924	0.83556	0.18503
**7**	C	C007	0.50493	0.98766	0.21439	**68**	C	C068	0.78819	0.88967	0.18122
**8**	C	C008	0.61576	0.04771	0.20641	**69**	C	C069	0.84265	0.83339	0.1846
**9**	C	C009	0.67246	0.9948	0.19486	**70**	C	C070	0.95368	0.8885	0.18602
**10**	C	C010	0.78385	0.05182	0.18734	**71**	O	O001	0.77381	0.32808	0.39134
**11**	C	C011	0.84259	0.00003	0.18162	**72**	O	O002	0.38405	0.68486	0.33293
**12**	C	C012	0.95337	0.05531	0.18317	**73**	Cu	Cu001	0.31579	0.32768	0.51701
**13**	C	C013	0.00783	0.16632	0.18584	**74**	Cu	Cu002	0.32032	0.25266	0.87984
**14**	C	C014	0.11897	0.22262	0.19198	**75**	Cu	Cu003	0.36505	0.28959	0.72085
**15**	C	C015	0.175	0.16704	0.19555	**76**	Cu	Cu004	0.33277	0.43378	0.64825
**16**	C	C016	0.28567	0.22364	0.20323	**77**	Cu	Cu005	0.45132	0.29325	0.45797
**17**	C	C017	0.34167	0.16717	0.20938	**78**	Cu	Cu006	0.45972	0.22393	0.83571
**18**	C	C018	0.45195	0.224	0.22089	**79**	Cu	Cu007	0.52136	0.27509	0.68869
**19**	C	C019	0.50856	0.16549	0.2213	**80**	Cu	Cu008	0.47402	0.38525	0.59735
**20**	C	C020	0.61837	0.21413	0.22619	**81**	Cu	Cu009	0.68534	0.33664	0.3046
**21**	C	C021	0.67182	0.15997	0.20989	**82**	Cu	Cu010	0.60962	0.20751	0.80063
**22**	C	C022	0.78222	0.21604	0.19784	**83**	Cu	Cu011	0.66324	0.24172	0.64799
**23**	C	C023	0.83843	0.16287	0.18921	**84**	Cu	Cu012	0.60677	0.33733	0.54428
**24**	C	C024	0.9499	0.21945	0.1863	**85**	Cu	Cu013	0.32186	0.4925	0.49081
**25**	C	C025	0.00312	0.33005	0.18942	**86**	Cu	Cu014	0.34023	0.41057	0.81216
**26**	C	C026	0.1137	0.38623	0.19605	**87**	Cu	Cu015	0.50817	0.51796	0.7175
**27**	C	C027	0.1721	0.33361	0.19857	**88**	Cu	Cu016	0.35133	0.60925	0.61341
**28**	C	C028	0.28339	0.39227	0.20626	**89**	Cu	Cu017	0.46536	0.46132	0.43046
**29**	C	C029	0.33853	0.3358	0.20485	**90**	Cu	Cu018	0.49441	0.40003	0.8465
**30**	C	C030	0.44849	0.39502	0.21064	**91**	Cu	Cu019	0.6421	0.47096	0.67712
**31**	C	C031	0.50311	0.33748	0.2255	**92**	Cu	Cu020	0.49499	0.57237	0.55896
**32**	C	C032	0.61027	0.39893	0.25077	**93**	Cu	Cu021	0.61307	0.43464	0.38272
**33**	C	C033	0.78232	0.38313	0.20277	**94**	Cu	Cu022	0.64629	0.3843	0.81889
**34**	C	C034	0.83525	0.32763	0.19568	**95**	Cu	Cu023	0.78082	0.4306	0.61184

**35**	C	C035	0.94635	0.38447	0.19148	**96**	Cu	Cu024	0.63279	0.51613	0.52395
**36**	C	C036	0.9997	0.49521	0.19606	**97**	Cu	Cu025	0.34788	0.67467	0.46813
**37**	C	C037	0.10978	0.55067	0.20555	**98**	Cu	Cu026	0.37991	0.57805	0.76484
**38**	C	C038	0.16557	0.49596	0.20836	**99**	Cu	Cu027	0.53563	0.70665	0.6794
**39**	C	C039	0.27287	0.55001	0.23118	**100**	Cu	Cu028	0.47548	0.79344	0.57877
**40**	C	C040	0.33609	0.50377	0.21893	**101**	Cu	Cu029	0.46672	0.62578	0.39306
**41**	C	C041	0.4483	0.57109	0.21127	**102**	Cu	Cu030	0.50741	0.57074	0.87103
**42**	C	C042	0.50218	0.50953	0.20719	**103**	Cu	Cu031	0.66665	0.65786	0.63501
**43**	C	C043	0.61285	0.55862	0.19966	**104**	Cu	Cu032	0.61445	0.75723	0.53168
**44**	C	C044	0.6675	0.50274	0.21517	**105**	Cu	Cu033	0.62724	0.62998	0.41171
**45**	C	C045	0.77836	0.55326	0.20345	**106**	Cu	Cu034	0.66427	0.55743	0.82397
**46**	C	C046	0.83123	0.49361	0.19977	**107**	Cu	Cu035	0.80578	0.60939	0.59437
**47**	C	C047	0.94409	0.55046	0.19553	**108**	Cu	Cu036	0.77608	0.74882	0.50392
**48**	C	C048	0.00215	0.66253	0.19551	**109**	Cu	Cu037	0.72136	0.27095	0.50222
**49**	C	C049	0.11361	0.71851	0.20245	**110**	Cu	Cu038	0.74809	0.18098	0.7465
**50**	C	C050	0.16617	0.66113	0.21228	**111**	Cu	Cu039	0.77394	0.44607	0.44863
**51**	C	C051	0.27379	0.71422	0.23521	**112**	Cu	Cu040	0.78575	0.35871	0.76082
**52**	C	C052	0.31414	0.65152	0.26835	**113**	Cu	Cu041	0.78435	0.60821	0.42964
**53**	C	C053	0.50903	0.69023	0.20291	**114**	Cu	Cu042	0.80887	0.53333	0.74882
**54**	C	C054	0.61697	0.72615	0.18886	**115**	Cu	Cu043	0.40073	0.8608	0.47798
**55**	C	C055	0.67002	0.66813	0.18913	**116**	Cu	Cu044	0.40117	0.74896	0.72657
**56**	C	C056	0.78269	0.72179	0.19051	**117**	Cu	Cu045	0.5336	0.82419	0.42464
**57**	C	C057	0.83693	0.66394	0.19408	**118**	Cu	Cu046	0.51465	0.73673	0.83707
**58**	C	C058	0.94899	0.71991	0.1922	**119**	Cu	Cu047	0.68928	0.81749	0.39023
**59**	C	C059	0.00766	0.83156	0.19118	**120**	Cu	Cu048	0.66105	0.71545	0.7903
**60**	C	C060	0.11909	0.88718	0.19811	**121**	Cu	Cu049	0.79509	0.74738	0.33691
**61**	C	C061	0.17282	0.83011	0.20601	**122**	Cu	Cu050	0.8067	0.69535	0.73491

3. References

[1] B. Ravel, M. Newville, ATHENA, ARTEMIS, HEPHAESTUS: data analysis for X-ray absorption spectroscopy using IFEFFIT, J. Synchrotron Radiat., 12(2005) 537-541, https://doi.org/10.1107/S0909049505012719.
[2] S. I. Zabinsky, J. J. Rehr, A. Ankudinov, R. C. Albers, M. J. Eller, Multiple-scattering calculations of x-ray-absorption spectra, Phys. Rev. B., 52(1995) 2995-3009, https://doi.org/10.1103/PhysRevB.52.2995.
[3] J. P. Perdew, K. Burke, M. Ernzerhof, Generalized Gradient Approximation Made Simple, Phys. Rev. Lett., 78(1997) 1396, https://doi.org/10.1103/PhysRevLett.77.3865.
[4] G. Kresse, J. Furthmuller, Efficient Iterative Schemes for ab Initio Total-energy Calculations Using a Plane-wave Basis Set, Phys. Rev. B, 54(1996) 11169-11186, https://doi.org/10.1103/PhysRevB.54.11169.
[5] G. Kresse, D. Joubert, From ultrasoft pseudopotentials to the projector augmented-wave method, Phys. Rev. B., 59(1999) 1758, https://doi.org/10.1103/PhysRevB.59.1758.
[6] S. Grimme, J. Antony, S. Ehrlich, S. Krieg, A consistent and accurate ab initio parametrization of density functional dispersion correction (DFT-D) for the 94 elements H-Pu, J. Chem. Phys. 132(2010) 154104, https://doi.org/10.1063/1.3382344.
[7] H. J. Monkhorst, J. D. Pack, Special points for Brillouin-zone integrations, Phys. Rev. B., 13(1976) 5188-5192, https://doi.org/10.1103/PhysRevB.13.5188.
[8] G. Gao, J. Remon, Z. Jiang, L. Yao, C. Hu, Selective hydrogenation of furfural to furfuryl alcohol in water under mild conditions over a hydrotalcite-derived Pt-based catalyst, Appl. Catal. B, 309 (2022) 121260, https://doi.org/10.1016/j.apcatb.2022.121260.
[9] X. Zhang, R. Shen, X. Guo, X. Yan, Y. Chen, J. Hu, W. Lang. Bimetallic Ag-Cu nanoparticles anchored on polypropylene (PP) nonwoven fabrics: Superb catalytic efficiency and stability in 4-nitrophenol reduction, Chem. Eng. J., 408 (2021) 128018, https://doi.org/10.1016/j.cej.2020.128018.
[10] B. Feng, R. Guo, Q. Cai, Y. Song, N. Li, Y. Fu, D. L Chen, J. Zhang, W. Zhu, F. Zhang, Construction of isolated Ni sites on nitrogen-doped hollow carbon spheres with $Ni-N_3$ configuration for enhanced reduction of nitroarenes, Nano Res., 15 (2022) 6001-6009, https://doi.org/10.1007/s12274-022-4290-x.
[11] W. C. Cheong, W. Yang, J. Zhang, Y. Li, D. Zhao, S. Liu, K. Wu, Q. Liu, C.o Zhang, D. Wang, Q. Peng, C. Chen, Y. Li, Isolated Iron Single-Atomic Site-Catalyzed Chemoselective Transfer Hydrogenation of Nitroarenes to Arylamines, ACS Appl. Mater. Interfaces, 11 (2019) 33819-33824, https://doi.org/10.1021/acsami.9b09125.
[12] S. Kim, E. Kim, B. M. Kim, Fe_3O_4 Nanoparticles: A Conveniently Reusable Catalyst for the Reduction of Nitroarenes Using Hydrazine Hydrate, Chem. Asian J., 6 (2011) 1861-4728, https://doi.org/10.1002/asia.201100311.
[13] C. Jiang, Z. Shang, X. Liang, Chemoselective Transfer Hydrogenation of Nitroarenes Catalyzed by Highly Dispersed, Supported Nickel Nanoparticles, ACS Catal., 5 (2015) 4814-4818, https://doi.org/10.1021/acscatal.5b00969.
[14] S. Chen, L. L. Ling, S. F. Jiang, H. Jiang, Selective hydrogenation of nitroarenes under mild conditions by the optimization of active sites in a well defined Co@NC catalyst, Green Chem., 22 (2020) 5730-5741, https://doi.org/10.1039/D0GC01835J.

[15] Z. Zhao, H. Yang, Yu Lia, X. Guo, Cobalt-modified molybdenum carbide as an efficient catalyst for chemoselective reduction of aromatic nitro compounds, Green Chem., 16 (2014) 1274-1281, https://doi.org/10.1039/C3GC42049C.
[16] M. L. Gao, L. Li, Z. X. Sun, J. R. N. Li, H. L. Jiang, Facet Engineering of a Metal-Organic Framework Support Modulates the Microenvironment of Palladium Nanoparticles for Selective Hydrogenation, Angew. Chem. Int. Ed., 61 (2022) 1433-7851, https://doi.org/10.1002/anie.202211216.
[17] W. Zhang, W. Wu, Y. Long, F. Wang, J. Ma, Co-Ag alloy protected by nitrogen doped carbon as highly efficient and chemoselective catalysts for the hydrogenation of halogenated nitrobenzenes, J. Colloid Interface Sci., 522 (2018) 217-227, https://doi.org/10.1016/j.jcis.2018.03.059.
[18] J. Liu, J. Li, R. Meng, P. Jian, L. Wang, Silver nanoparticles-decorated-Co_3O_4 porous sheets as efficient catalysts for the liquid-phase hydrogenation reduction of p-Nitrophenol, J. Colloid Interface Sci., 551 (2019) 261-269, https://doi.org/10.1016/j.jcis.2019.05.018.
[19] F. Chen, X. Yan, X. Hu, R. Feng, T. Li, Xiaobing Li, G. Zhao, Enhanced catalytic reduction of p-nitrophenol and azo dyes on copper hexacyanoferrate nanospheres decorated copper foams, J ENVIRON MANAGE, 314 (2022) 115075, https://doi.org/10.1016/j.jenvman.2022.115075.
[20] X. Sun, P. He, Z. Gao, Y. Liao, S. Weng, Z. Zhao, H. Song, Z. Zhao, Multi-crystalline N-doped Cu/CuO/C foam catalyst derived from alkaline N-coordinated HKUST-1/CMC for enhanced 4-nitrophenol reductionx, J. Colloid Interface Sci., 553 (2019) 1-13, https://doi.org/10.1016/j.jcis.2019.06.004.
[21] M. Zhang, X. Su, L. Ma, A. Khan, L. Wang, J. Wang, A. S. Maloletnev, C. Yang, Promotion effects of halloysite nanotubes on catalytic activity of CoO nanoparticles toward reduction of 4-nitrophenol and organic dyes, J. Hazard., 403 (2021) 123870, https://doi.org/10.1016/j.jhazmat.2020.123870.
[22] X. Zhang, N. Wang, L. Geng, J. Fu, H.Hu, D. Zhang, B. Zhu, J. Carozza, H. Han, Facile synthesis of ultrafine cobalt oxides embedded into N-doped carbon with superior activity in hydrogenation of 4-nitrophenol, J. Colloid Interface Sci., 512 (2018) 844-852, https://doi.org/10.1016/j.jcis.2017.11.005.
[23] F. Chen, X. Yan, X. Hu, R. Feng, T. Li, X. Li, G. Zhao, Enhanced catalytic reduction of p-nitrophenol and azo dyes on copper hexacyanoferrate nanospheres decorated copper foams, J ENVIRON MANAGE, 314 (2022) 115075, https://doi.org/10.1016/j.jenvman.2022.115075.
[24] C. S. Chen, T. C. Chen, K. L. Chiu, H. C. Wu, C. W. Pao, C. L. Chen, H. C. Hsu, H. M. Kao, Silver particles deposited onto magnetic carbon nanofibers as highly active catalysts for 4-nitrophenol reduction, Appl. Catal. B., 315 (2022) 121596, https://doi.org/10.1016/j.apcatb.2022.121596.
[25] A. Zhou, J. L. hui, W.Q. Xu, Preparation of Ag/ZrGP nanocomposites with enhanced catalytic activity for catalytic reduction of 4-nitrophenol, Appl. Surf. Sci., 506 (2020) 144570, https://doi.org/10.1016/j.apsusc.2019.144570.
[26] M. Fan, W. Wang, Y. Zhu, X. Sun, F. Zhang, Z. Dong, Palladium clusters confined in triazinyl-functionalized COFs with enhanced catalytic activity, Appl. Catal. B., 257 (2019) 117942, https://doi.org/10.1016/j.apcatb.2019.117942.
[27] K. Gu, X. Pan, W. Wang, J. Ma, Y. Sun, H. Yang, H. Shen, Z. Huang, H. Liu, In Situ Growth of Pd Nanosheets on g-C_3N_4 Nanosheets with Well-Contacted Interface and Enhanced Catalytic Performance for 4-Nitrophenol Reduction, Small, 14 (2018) 1613-6810, https://doi.org/10.1002/smll.201801812.
[28] F. Subhan, S. Aslam, Z. Yan, M. Yaseen, K. A. Khan, Palladium nanoparticles decorated on ZSM-5 derived micro-/mesostructures (MMZ) for nitrophenol reduction and MB degradation in water, J. Environ. Chem. Eng., 9 (2021) 105002, https://doi.org/10.1016/j.jece.2020.105002.

[29] J. Kou, W. Wang, J. Fang, F. Li, H. Zhao, J. Li, H. Zhu, B.Li, Z. Dong, Precisely controlled Pd nanoclusters confined in porous organic cages for size-dependent catalytic hydrogenation, Appl. Catal. B., 315 (2022) 121487, https://doi.org/10.1016/j.apcatb.2022.121487.
[30] M. Tian, X. Xu, D. Dong, Palladium nanoparticles dispersed on the hollow aluminosilicate microsphere@hierarchical γ-AlOOH as an excellent catalyst for the hydrogenation of nitroarenes under ambient conditions, Appl. Surf. Sci., 390 (2016) 100-106, https://doi.org/10.1016/j.apsusc.2016.08.073
[31] R. Ding, Q. Chen, Q. Luo, L. Zhou, Y. Wang, Y. Zhang, G. Fan, Salt template-assisted in situ construction of Ru nanoclusters and porous carbon: excellent catalysts toward hydrogen evolution, ammonia-borane hydrolysis, and 4-nitrophenol reduction, Green Chem., 22 (2020) 835-842,https://doi.org/10.1039/C9GC03986D.
[32] D. Gao, X. Zhang, X. Dai, Y. Qin, A. Duan, Y. Yu, H. Zhuo, H. Zhao, P. Zhang, Y. Jiang, J. Li, Z. Zhao, Morphology-selective synthesis of active and durable gold catalysts with high catalytic performance in the reduction of 4-nitrophenol, Nano Res., 9 (2016) 3099-3115, https://doi.org/10.1007/s12274-016-1193-8.
[33] X. Yang, H. Jin, Y. Yao, S. Lin, Construction of 3D Ordered Honeycomb Films with Controllable Pores as Efficient Catalytic Supports, Adv. Funct. Mater., 32 (2022) 2202298, https://doi.org/10.1002/adfm.202202298.
[34] T. B. Nguyen, C. P. Huang, R. Doong, Enhanced catalytic reduction of nitrophenols by sodium borohydride over highly recyclable Au@graphitic carbon nitride nanocomposites, Appl. Catal. B., 240 (2019) 337-347, https://doi.org/10.1016/j.apcatb.2018.08.035.
[35] Y. Xu, X. Shi, Rui HuaRui, Z. jin, Y. Z. Tong, L. zheng, Z. Lu, Remarkably catalytic activity in reduction of 4-nitrophenol and methylene blue by FeO@COF supported noble metal nanoparticles, Appl. Catal. B., 260 (2020) 118142, https://doi.org/10.1016/j.apcatb.2019.118142.
[36] P. Ilgin, O. Ozay, H. Ozay, A novel hydrogel containing thioether group as selective support material for preparation of gold nanoparticles: Synthesis and catalytic applications, Appl. Catal. B., 9 (2019) 415-423, https://doi.org/10.1016/j.apcatb.2018.09.066.
[37] J. J. Musci, M. Montaña, A. B. Merlo, R. A. A. Cecilia, E. C. D. Lick, M. L. Casella, Supported ruthenium catalysts for the aqueous-phase selective hydrogenation of furfural to furfuryl alcohol, Catal. Today, 394-396 (2022) 81-93, https://doi.org/10.1016/j.cattod.2021.12.011.
[38] M. G. Dohade, P. L. Dhepe, Efficient hydrogenation of concentrated aqueous furfural solutions into furfuryl alcohol under ambient conditions in presence of PtCo bimetallic catalyst, Green Chem., 19 (2017) 1144-1154, https://doi.org/10.1039/C6GC03143A.
[39] K. Yang, Y. Li , R. Wang, Q. Li, B. Huang, X. Guo, Z. Zhu, T. Su, H. Lü, Synthesis of Dual-Active-Sites Ni-NiIn catalysts for selective hydrogenation of furfural to furfuryl alcohol, Fuel, 325 (2022) 124898, https://doi.org/10.1016/j.fuel.2022.124898.
[40] Y. Tang, M. Qiu, J. Yang, F. Shen, X. Wang, X. Qi, One-pot self-assembly synthesis of Ni-doped ordered mesoporous carbon for quantitative hydrogenation of furfural to furfuryl alcohol, Green Chem., 23 (2021) 1861-1870, https://doi.org/10.1039/D0GC04029K.
[41] L. Luo, F. Yuan, F. Zaer, Y. Zhu, Catalytic hydrogenation of furfural to furfuryl alcohol on hydrotalcite-derived $Cu_xNi_{3-x}AlO_y$ mixed-metal oxides, J. Catal., 404 (2021) 420-429, https://doi.org/10.1016/j.jcat.2021.10.009.
[42] P. Cao, L. Lin, H. Qi, R. Chen, Z. Wu, N. Li, T. Zhang, W. Luo, Zeolite-Encapsulated Cu Nanoparticles for the Selective Hydrogenation of Furfural to Furfuryl Alcohol, ACS Catal., 11 (2021) 10246-10256, https://doi.org/10.1021/acscatal.1c02658.
[43] J. Yu, Y. Yang, L. Chen, Z. L. Liu, X. Y. jing, H. X. Zhang, M. Wei, NiBi intermetallic compounds catalyst toward selective hydrogenation of unsaturated aldehydes, Appl. Catal. B., 277 (2020) 119273, https://doi.org/10.1016/j.apcatb.2020.119273.

[44] J. Li, M. Zahid, W. Sun, X. Tian, Y. Zhu, Synthesis of Pt supported on mesoporous g-C_3N_4 modified by ammonium chloride and its efficiently selective hydrogenation of furfural to furfuryl alcohol, Appl. Surf. Sci., 528 (2020) 146983, https://doi.org/10.1016/j.apsusc.2020.146983.
[45] Y. Long, S. Song, J. Li, L. Wu, Q. Wang, Y. Liu, R. Jin, H. Zhang, Pt/CeO_2@MOF Core@Shell Nanoreactor for Selective Hydrogenation of Furfural via the Channel Screening Effect, ACS Catal., 8 (2018) 8506-8512, https://doi.org/10.1021/acscatal.8b01851.
[46] X. Wang, M. Qiu, R. L. Smith. Jr, J. Yang, Ferromagnetic lignin-derived ordered mesoporous carbon for catalytic hydrogenation of furfural to furfuryl alcohol, ACS Sustainable Chem. Eng., 8 (2020) 18157-18166, https://doi.org/10.1021/acssuschemeng.0c06533.
[47] K. E. Salnikov, V. G. Matveeva, Y. V. Larichev, A. V. Bykov, G. N. Demidenko, I. P. Shkileva, M. G. Sulman, The liquid phase catalytic hydrogenation of furfural to furfuryl alcohol, Catal. Today, 329 (2019) 142-148, https://doi.org/10.1016/j.cattod.2018.12.036.
[48] F. Lan, H. Zhang, C. Zhao, Y. Shu, Q. Guan, W. Li, Copper Clusters Encapsulated in Carbonaceous Mesoporous Silica Nanospheres for the Valorization of Biomass-Derived Molecules, ACS Catal., 12 (2022) 5711-5725, https://doi.org/10.1021/acscatal.2c01270.
[49] B. Seemala, C. M. Cai, C. E. Wyman, P. Christopher, Support Induced Control of Surface Composition in Cu-Ni/TiO_2 Catalysts Enables High Yield Co-Conversion of HMF and Furfural to Methylated Furans, ACS Sustainable Chem. Eng., 7 (2017) 4070-4082, https://doi.org/10.1021/acscatal.7b01095.
[50] H. Ishikawa, M. Sheng, A. Nakata, K. Nakajima, S. Yamazoe, J. Yamasaki, S. Yamaguchi, T. Mizugaki, T. Mitsudome, Air-stable and reusable cobalt phosphide nanoalloy catalyst for selective hydrogenation of furfural derivatives, ACS Catal., 11 (2021) 750-757, https://doi.org/10.1021/acscatal.0c03300.
[51] C. Wang, Y. Liu, Z. Cui, X. Yu, X. Zhang, Y. Li, Q. Zhang, L. Chen, L. Ma, In Situ Synthesis of Cu Nanoparticles on Carbon for Highly Selective Hydrogenation of Furfural to Furfuryl Alcohol by Using Pomelo Peel as the Carbon Source, ACS Sustainable Chem. Eng., 8 (2020) 12944-12955, https://doi.org/10.1021/acssuschemeng.0c03505.
[52] P. Jia, X. Lan, X. Li, T. Wang, Highly Active and Selective NiFe/SiO_2 Bimetallic Catalyst with Optimized Solvent Effect for the Liquid-Phase Hydrogenation of Furfural to Furfuryl Alcohol, ACS Sustainable Chem. Eng., 6 (2018) 13287-13295, https://doi.org/10.1021/acssuschemeng.8b02876.
[53] Y. Fan, S. Li, Y. Wang, C. Zhuang, X. Liu, G. Zhu, X. Zou, Tuning the synthesis of polymetallic-doped ZIF derived materials for efficient hydrogenation of furfural to furfuryl alcohol, Nanoscale, 12 (2020) 18296-18304, https://doi.org/10.1039/D0NR04098C.
